# Designing New Indene-Fullerene Derivatives as Electron-Transporting Materials for Flexible Perovskite Solar Cells

**DOI:** 10.1021/acs.jpcc.1c07189

**Published:** 2021-12-03

**Authors:** Lukasz Przypis, Taimoor Ahmad, Kasjan Misztal, Damian Honisz, Eros Radicchi, Edoardo Mosconi, Wojciech Domagala, Filippo De Angelis, Konrad Wojciechowski

**Affiliations:** † Saule Research Institute, Wroclaw Technology Park, 11 Dunska Street, Sigma Building, 54-130 Wrocław, Poland; ‡ Department of Organic Chemistry, Bioorganic Chemistry and Biotechnology, Silesian University of Technology, Bolesława Krzywoustego 4, 44-100 Gliwice, Poland; § Saule Technologies Ltd., Wroclaw Technology Park, 11 Dunska Street, Sigma Building, 54-130 Wrocław, Poland; ∥ Department of Electronics Engineering, University of Rome “Tor Vergata”, Via del Politecnico 1, 00133 Rome, Italy; ⊥ Department of Physical Chemistry and Technology of Polymers, Silesian University of Technology, Marcina Strzody 9, 44-100 Gliwice, Poland; # Computational Laboratory for Hybrid/Organic Photovoltaics (CLHYO), Istituto CNR di Scienze e Tecnologie Chimiche “Giulio Natta” (CNR-SCITEC), Via Elce di Sotto 8, 06123 Perugia, Italy; ¶ Department of Chemistry, Biology and Biotechnology, University of Perugia, Via Elce di Sotto 8, 06123 Perugia, Italy; ∇ CompuNet, Istituto Italiano di Tecnologia, Via Morego 30, 16163 Genova, Italy; ○ Department of Mechanical Engineering, College of Engineering, Prince Mohammad Bin Fahd University, P.O. Box 1664, 31952 Al Khobar, Kingdom of Saudi Arabia

## Abstract

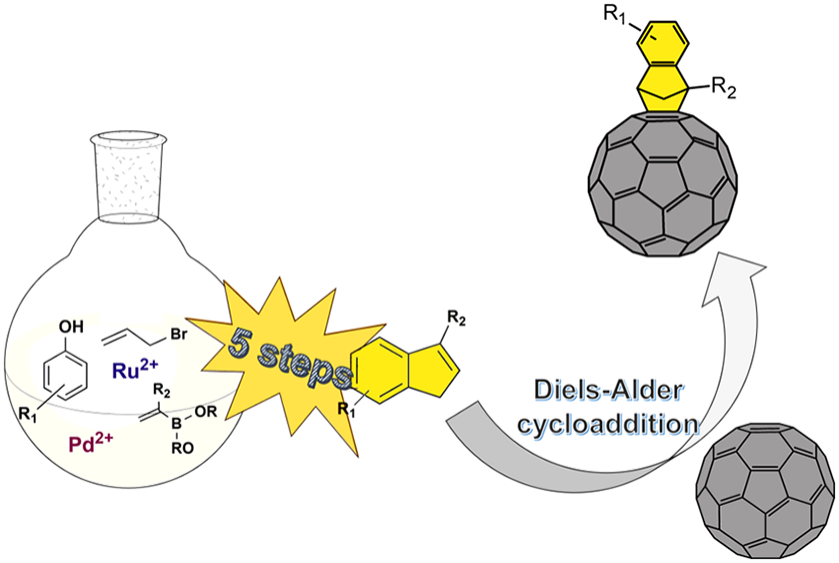

The synthesis and characterization of a family of indene-C_60_ adducts obtained *via* Diels–Alder cycloaddition [4 + 2] are reported. The new C_60_ derivatives include indenes with a variety of functional groups. These adducts show lowest unoccupied molecular orbital energy levels to be at the right position to consider these compounds as electron-transporting materials for planar heterojunction perovskite solar cells. Selected derivatives were applied into inverted (p–i–n configuration) perovskite device architectures, fabricated on flexible polymer substrates, with large active areas (1 cm^2^). The highest power conversion efficiency, reaching 13.61%, was obtained for the 6′-acetamido-1′,4′-dihydro-naphtho[2′,3′:1,2][5,6]fullerene-C_60_ (**NHAc-ICMA**). Spectroscopic characterization was applied to visualize possible passivation effects of the perovskite’s surface induced by these adducts.

## Introduction

**Figure 1:**
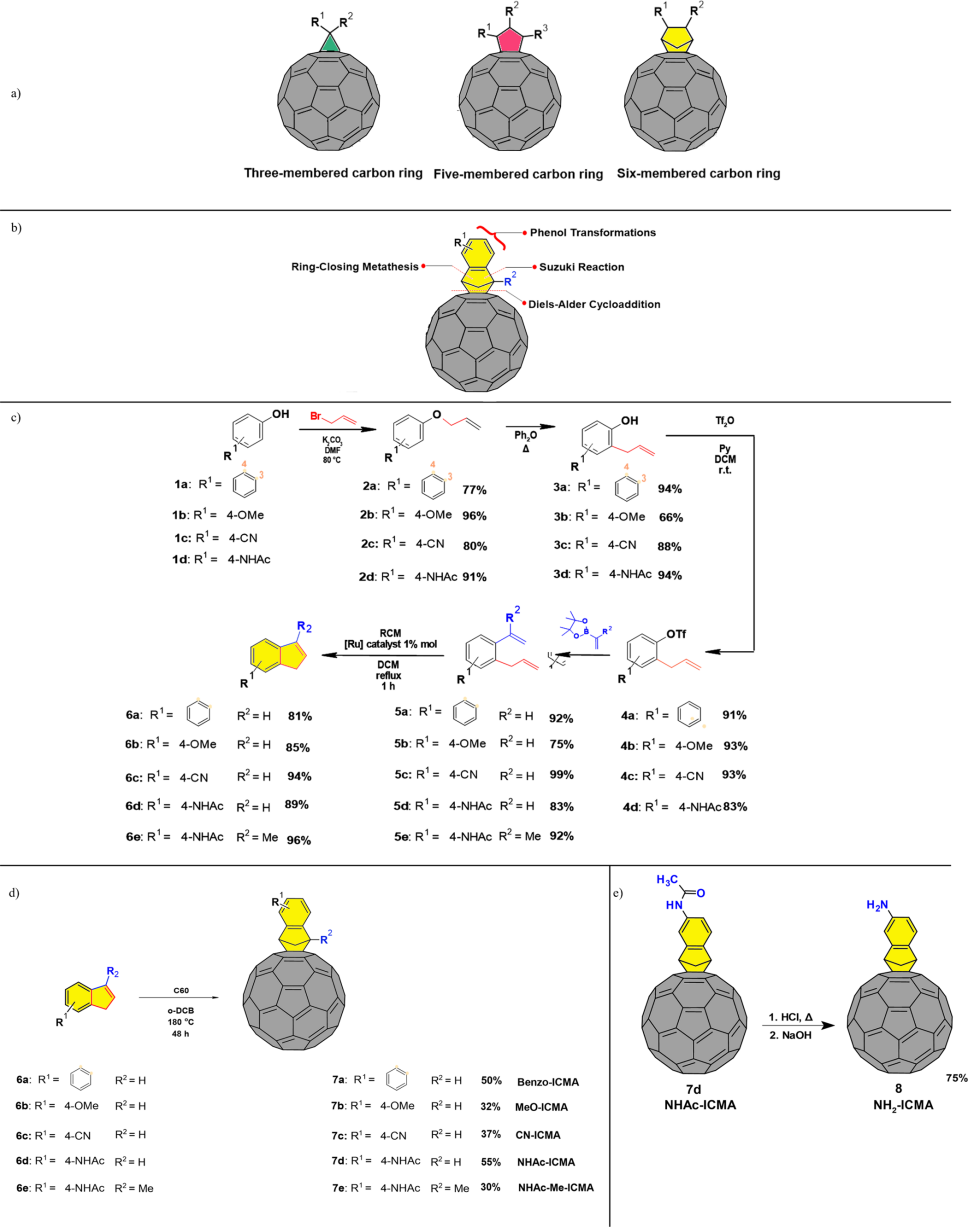
Depiction of a general strategy for obtaining substituted indene-fullerene adducts, (a) possible geometric hydrocarbon motifs appended to a 5, 6 ring junction of C_60_, (b) retrosynthetic breakdown of an indene-fullerene adduct, (c) reaction pathway to obtain substituted indenes, (d) Diels–Alder [4 + 2] cycloaddition of substituted indenes with C_60_, and (e) synthesis of a free-amine indene-fullerene derivative.

Since milligram-scale syntheses of fullerene (C_60_) got into gear in the early 1990s,^1−5^ many research groups provided evidence of their remarkable structural,^6,7^ magnetic,^8^ superconducting,^9^ electrochemical,^10^ photophysical,^11^ and biological properties.^12,13^ Currently, fullerenes are being utilized in a wide range of applications, including photovoltaics, light-emitting devices, modern antiviral therapies, or space exploration.^14−18^ In particular, fullerenes and a wide range of their derivatives have been successfully used as electron transport materials (ETMs), at first in organic solar cells, and more recently in perovskite solar cells (PSCs).^19−21^ This family of compounds is characterized by good electron-accepting properties (effective electron extraction from a photoabsorber), low-temperature processing, and suitable energy levels, enabling the role of electron-selective contact in these photovoltaic technologies. In PSCs, C_60_ and its derivatives, such as phenyl-C_61_-butyric acid methyl ester (**PCBM**), were predominantly used in a p–i–n device configuration (so called, “inverted” architecture), yielding high power conversion efficiencies (PCEs).^22−25^ The applicability of the pristine fullerene molecules is limited by solubility constraints in the most common solvents.^26^ Modifications of their chemical structure are required to solve this drawback. There are two general approaches toward the fullerene functionalization—covalent and non-covalent interactions.^27−29^ The covalent-based approach overall gives more options for a chemical modification. It can be realized by taking advantage of the electrophilic nature of the C_60_ or using conjugated π-electrons (particularly the [5,6] double bonds),^30^ which enables the molecule to undergo Diels–Alder reactions ([4 + 2] cycloadditions), acting as a dienophile.^31−37^ These two well-known synthetic pathways provided readily accessible, often quite sophisticated, C_60_-based building blocks for numerous application fields. The fullerene adducts can be categorized in classes depending on the number of carbon atoms of the substituent, as schematically reported in Figure 1a. The full carbon ring of C_60_ shows good performance in PSCs because of high electrochemical stability of its negatively charged reduction products.^38,39^ The three-membered carbon ring constitutes one of the most investigated types of a fullerene appendage. The C_60_ fullerenes with a five-membered carbon ring, namely, indano-C_60_, are still relatively unexplored.^40^ The indene-C_60_ adducts were synthesized by the Diels–Alder cycloaddition.^31,37^ High yields can be obtained for this methodology, but the scope of molecules which can be synthesized so far has been limited by availability of appropriate indene derivative precursors. The first indenyl derivative, the fullerene-indene-C_60_ bisadduct (ICBA), was reported in 2010.^41^ It was designed and synthesized as an alternative to **PCBM**, for use in polymer solar cells.^41^ In 2013, Chen was the first to use ICBA as an electron-transporting material (ETM) in a planar heterojunction p–i–n PSC architecture. Despite the promising results obtained for these indene–fullerene adducts, the topic was not further explored due to the aforementioned limitations. Herein, we report a methodology to obtain indene-fullerene adducts based on simple and cheap substrates. We synthesized a group of indene derivatives, equipped with functional groups of different electronic natures, electron-releasing (*e.g.*, −OMe and −NH_2_) and electron-withdrawing moieties (*e.g.*, −CN). We used phenol derivatives as starting materials and completed the process in five steps, with the final yield of 50–60% (Figure 1b–e).^42^ To the best of our knowledge, this is the first demonstration of this method in the synthesis of indene-fullerene derivatives. Subsequently, the newly made indenes were employed in the synthesis of several indene-fullerene C_60_ derivatives. We characterized basic photophysical and electrochemical properties of these compounds as a preliminary assessment of their device implementation potential. Lastly, we incorporated selected fullerene derivatives to the inverted PSC architecture as an ETL and compared the effect of different fullerene modifying groups.

## Results and Discussion

We synthesized a series of substituted indenes in a five-step process, which we schematically present in Figure 1c. The starting material in the reaction sequence was allyl bromide and an appropriate phenol derivative: 2-naphthol, 4-methoxyphenol, 4-cyanphenol, or 4-acetamidephenol. The substituted indene precursors, **6a–6e**, obtained were subsequently transformed by a ring-closing metathesis to afford an excellent yield, as shown in Figure 1b. Full experimental data and spectroscopic characterizations are provided in the Supporting Information, Section 1.3 and 1.4.3.

**Figure 2:**
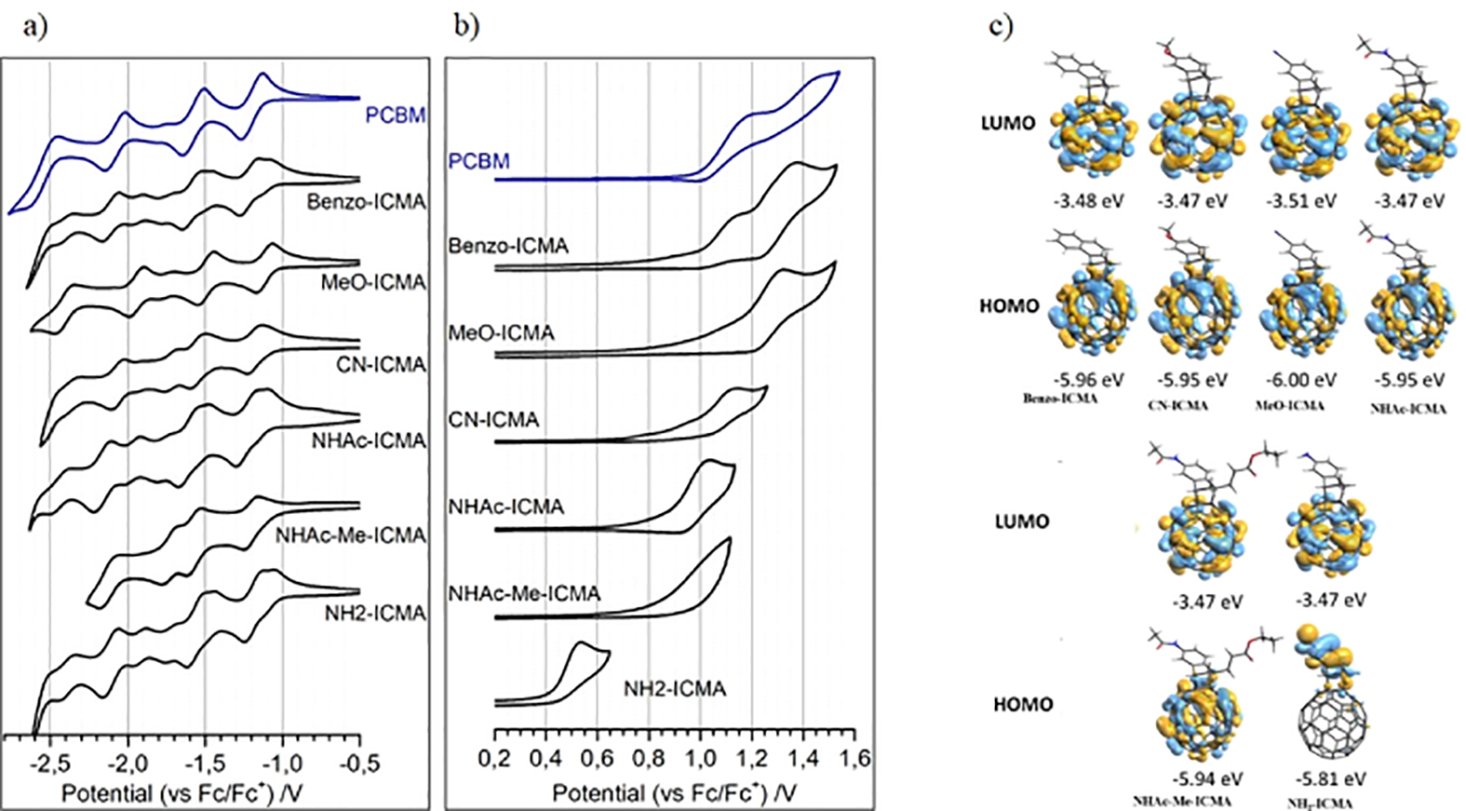
Cyclic voltammograms of (a) reduction and (b) oxidation processes of fullerene compounds, recorded in a solution of 0.05 M tetrabutylammonium hexafluorophosphate in *o*-DCB; (c) HOMO and LUMO energies and shapes of the investigated substituted indene-fullerene adducts.

Finally, indene-fullerene adducts were obtained in a Diels–Alder reaction. First, at the reflux temperature, indene is undergoing [1,2]-hydrogen shift, yielding isoindene, which subsequently reacts in the Diels–Alder [4 + 2] cycloaddition (Figure 1d).^43^ The final products, **Benzo-ICMA**, **MeO-ICMA**, **CN-ICMA**, **NHAc-ICMA**, and **NHAc-Me-ICMA**, were synthesized by carrying out this process in *ortho*-dichlorobenzene (*o*-DCB), at 180 °C for 48 h. All the final adducts were purified by column chromatography in toluene/ethyl acetate, except for the **MeO-ICMA**. This product is metastable and decomposed to the indene precursor and C_60._ For the amine-substituted indene, in the early steps of the proposed synthesis route, the 4-aminophenol can be transformed to undesired byproducts. Therefore, a fullerene derivative with the amino group had to be synthesized from a fullerene acetamide derivative, Figure 1e. There are many examples of such post-modification of functionalized fullerenes in the literature.^44−46^ We obtained this derivative reaching a high yield of 75%. The highest occupied molecular orbital (HOMO) and the lowest unoccupied molecular orbital (LUMO) levels of fullerene derivatives are of great importance for considering their use as electron acceptors and hole-blocking materials in PSCs.^47,48^ We derived experimental values of these energy levels by cyclic voltammetry (CV) measurements (Figure 2a–c), which are presented in Table 1; the methodology is further elaborated in the Supporting Information, Section 1.2.1. The CV measurements of all the considered fullerene derivatives showed successive reversible reduction steps, typical of fullerene electrochemistry (Figure 2a). The functionalization of the fullerene core with the indene moiety shifts the onset of the first reduction potential to more negative values when compared to the pristine C_60_,^49^ indicating an electron-releasing effect of the indene moiety (see Figures S4–S10). The cathodic CV trace of **PCBM** features four reversible redox steps, corresponding to successive single-electron charging of the fullerene’s π-conjugated structure. By comparing the CV traces of the investigated compounds with **PCBM**, it is possible to discriminate redox peaks of the fullerene core from other signatures, characteristic for each indene-fullerene derivatives.

**Table 1:** Electrochemically and Theoretically Derived Electronic Properties of the Fullerene Derivative Compounds

compound	*E*_red_^onset^ [V]	*E*_ox_^onset^ [V]	EA^a^^,^^b^ [eV]	IP^a^^,^^b^ [eV]	*E*_g_^b^ [eV]	LUMO^c^ [eV]	HOMO^c^ [eV]	H–L gap^c^ [eV]
**PCBM**	–1.13	1.00	3.97 (3.59)	6.10 (5.89)	2.13 (2.30)	–3.50	–6.00	2.50
**Benzo-ICMA**	–1.05	0.98	4.05 (3.57)	6.08 (5.85)	2.03 (2.28)	–3.48	–5.96	2.48
**MeO-ICMA**	–1.05	1.10	4.05 (3.56)	6.20 (5.84)	2.15 (2.28)	–3.47	–5.95	2.48
**CN-ICMA**	–1.05	0.94	4.05 (3.60)	6.04 (5.90)	1.99 (2.30)	–3.51	–6.00	2.49
**NHAc-ICMA**	–1.08	0.87	4.02 (3.57)	5.97 (5.84)	1.95 (2.27)	–3.47	–5.95	2.48
**NHAc-Me-ICMA**	–1.04	0.82	4.06 (3.56)	5.92 (5.83)	1.86 (2.27)	–3.47	–5.94	2.47
**NH**_**2**_**-ICMA**	–1.02	0.43	4.08 (3.56)	5.53 (5.53)	1.45 (1.97)	–3.47	–5.81	2.34

a EA and IP values for the ferrocene/ferrocenium (Fc/Fc^+^) redox couple (5.10 eV above the vacuum level) as an internal standard.

b *E*_g_ is calculated from the difference between the EA and IP from experimental data.

c Calculated by DFT at the B3LYP/6-311++G** level of theory with *o*-DCB as an implicit solvent. Theoretical values are given in parentheses, when reported along with experimental data.

In the anodic branch, all the investigated fullerenes oxidize irreversibly (Figure 2b), displaying onset potentials ranging from 0.43 V (for the **NH**_**2**_**-ICMA**) up to 1.10 V (for the **MeO-ICMA**) (see Table 1, Figures S7 and S8). This variation in the oxidation propensity, however, does not correlate with the electron-withdrawing or electron-releasing character of the indene moiety’s functional groups. Except for the **MeO-ICMA**, all the other investigated compounds oxidize at lower potentials than **PCBM**, indicating stronger electron communication of the indene pendant with the fullerene host than of the phenyl ring in **PCBM**. Interestingly, this communication facilitates electron abstraction (oxidation) from the fullerene-indene derivatives when compared to **PCBM**, which may be puzzling, since an analogous effect was observed for the process of electron introduction (reduction). This might point to a through-space interaction of the π-electrons of the fullerene and π-electrons of the indene units, sterically locked in position and inclined at a smaller angle above the fullerene core than the rotationally unrestricted phenyl pendant of **PCBM** (see Figure S11, Supporting Information, Section 1.4.1). The comparable oxidation onset potentials recorded for **NHAc-ICMA** and **NHAc-Me-ICMA** indicate a marginal inductive electronic effect of the functionalization of the ternary (>CH−) or quaternary (>C<) carbon bridge atoms, which interface the fullerene and phenylene units. Considering the ferrocene/ferrocenium (Fc/Fc^+^) redox couple (5.10 eV below the vacuum level) as the internal standard, we determined experimentally the electron affinity (EA) and first ionization potential (IP). All the values, including those for the **PCBM** molecule, are listed in Table 1. To gain insights into the electronic properties of the fullerene derivates, we also performed density functional theory (DFT) calculations to evaluate the EA, IP, the electrochemical band gap, and the shape of the HOMO and LUMO orbitals. From the Koopmans theorem and approximation,^50^ we compared the EA and IP with the calculated HOMO and LUMO values; the data and additional discussion about applied methodology are provided in Figures S12 and S13, Supporting Information, Sections 1.2.8 and 1.4.2. The CV measurements are in line with the theoretical predictions of the delocalization of the LUMOs in the studied set of molecules (see Figures 2c and S3). Additionally, in Table 1, we summarize the calculated HOMO values, which display good agreement with the measured IP values. On the other hand, an overestimation of the EA is found when compared to the LUMO values, and this consequentially leads to overestimation of the calculated HOMO–LUMO (H–L) band gap. All the investigated adducts show similar IP values, with the exception of the **NH**_**2**_**-ICMA**. The reason is that the HOMO level of this structure is destabilized and almost completely localized at the appendage, that is, on the aniline fragment of the indene moiety. The peculiar electronic properties of the **NH**_**2**_**-ICMA** are also reflected in its lower IP value taken from CV measurements, compared to the other compounds. This suggests the higher oxidation tendency of this compound. The calculated electrochemical energy gaps are almost the same for all the studied compounds; the only exception is the **NH**_**2**_**-ICMA** molecule, which displays a lower band gap, in line with the experimental findings.

**Figure 3:**
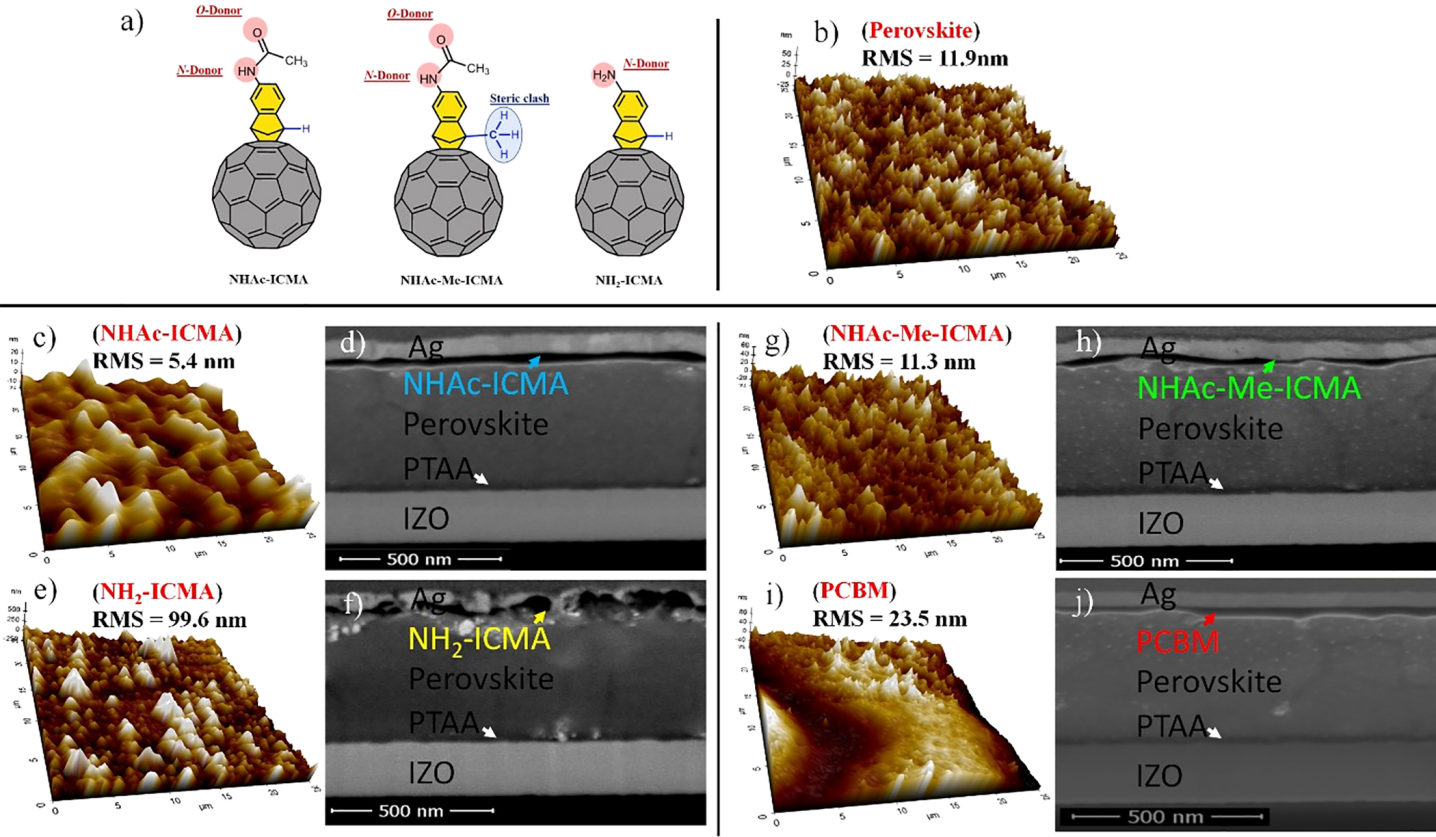
(a) Schematic structures of the indene-fullerene adducts applied in PSCs, (b) AFM 3D surface topography images (scanning range: 25 × 25 μm^2^) of the perovskite layer; and (c,e,g,i) perovskite coated with indene-fullerene films (**NHAc-ICMA**, **NH**_**2**_**-ICMA**, **NHAc-Me-ICMA**, and **PCBM**). (d,f,h,j) Cross-sectional FIB-SEM images of the perovskite layer coated with different fullerenes prepared on flexible substrates.

Based on the electronic properties, we selected the following compounds for further evaluation as potential ETMs in PSCs: the amide derivatives **NHAc-ICMA** and **NHAc-Me-ICMA** and the amine derivative **NH**_**2**_**-ICMA** (Figure 3a).^48^

Within this set of fullerene derivatives, we wanted to explore a potential passivation effect of the electronic defects present at the perovskite thin-film surface. It was shown that the most effective passivating agents are simultaneously inactivating both negatively and positively charged defects, exhibiting a zwitterionic effect.^51,52^ The fullerene moiety can act as a Lewis acid and passivate negatively charged defects (*e.g.*, undercoordinated I ions and Pb–I anti-sites).^53^ The amine bearing **–NH**_**2**_ tail and the amide group **–NHAc** are Lewis bases and play a significant role in passivating positively charged defects (*e.g.*, undercoordinated Pb^2+^ and Pb^2+^ interstitials).^54^ To investigate these effects, we first characterized electrical properties of the selected fullerene thin films. We coated layers of these materials on poly(ethylene naphthalate)/indium tin oxide (ITO) substrates, with a pre-patterned narrow trench in the ITO layer. We extracted conductivity values from the current–voltage curves (shown in Figure S2, Supporting Information). We obtained 3.20 × 10^–5^, 1.84 × 10^–5^, and 3.22 × 10^–5^ S cm^–1^ for **NHAc-ICMA**, **NH**_**2**_**-ICMA**, and **NHAc-Me-ICMA**, respectively. For the reference **PCBM**, we recorded a comparable value of 2.49 × 10^–5^ S cm^–1^. These results are consistent with the **PCBM** conductivity values reported in the literature.^55^ Next, we derived electron mobility values of the indene-fullerene adducts from the space charge-limited current measurements of electron-only devices, applying the Mott–Gurney law.^56^ We used the following device architecture: ITO/TiO_*X*_/ETL (varied fullerene derivatives)/TiO_*X*_/Ag (experimental details are provided in the Supporting Information). We extracted 1.50 × 10^–3^, 1.21 × 10^–3^, 2.24 × 10^–3^, and 3.40 × 10^–3^ cm^–2^ V^–1^ s^–1^ for **NHAc-ICMA**, **NH**_**2**_**-ICMA**, **NHAc-Me-ICMA**, and **PCBM**, respectively. In the literature, values reported for the **PCBM** films are typically in the range of 10^–3^ cm^–2^ V^–1^ s^–1^.^57−60^ The group of new indene-fullerene adducts shows slightly worse electron mobilities, which could influence transport characteristics in these films.

We also investigated the layer formation ability of the indene-fullerene derivatives. We fabricated thin films of different fullerene samples by spin coating relevant solutions on top of perovskite films (glass/perovskite substrates). As a perovskite material, we used the composition of mixed cations and mixed halides, Cs_0.04_(MA_0.17_FA_0.83_)_0.96_Pb(I_0.83_Br_0.17_)_3_, which was deposited using a solvent engineering strategy, following the method reported in our previous work.^24^ The layer morphologies were characterized by atomic force microscopy (AFM), and we derived the root-mean-square (RMS) roughness for each sample; for characterization details, see the Supporting Information, Section 1.2.3. The 3D surface topography and cross-sectional focused ion beam scanning electron microscopy (FIB-SEM) images are shown in Figure 3b–j. The bare perovskite layer and the perovskite/**NHAc-Me-ICMA** samples show an RMS of 11.9 and 11.3 nm, respectively. The other fullerene derivatives, **NHAc-ICMA**, **NH**_**2**_**-ICMA**, and **PCBM**, display RMS values of 5.4, 99.6, and 23.5 nm, respectively. Additionally, we note that **NHAc-ICMA** and **NHAc-Me-ICMA** demonstrated significantly higher solubility in *o*-DCB compared to **NH**_**2**_**-ICMA**. It is also evident from the FIB-SEM cross-sectional images that **NHAc-ICMA** displays more conformal and uniform coverage over the perovskite surface than the samples with **NH**_**2**_**-ICMA**, **NHAc-Me-ICAM**, and **PCBM**. More characterization details are provided in the Supporting Information, Section 1.2.4.

**Figure 4:**
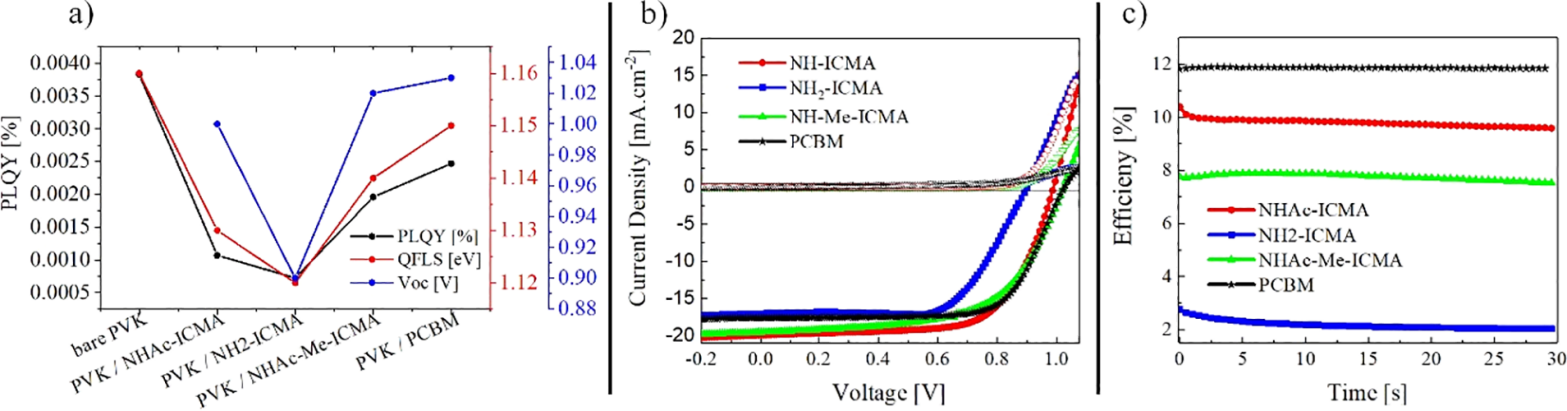
(a) Summary of the PLQY and calculated QFLS values determined from the spectroscopic measurements. We also include the implied open-circuit voltages determined for the solar cells representing the different fullerenes, (b) current density–voltage characteristics (light and dark) of the best PSCs with different fullerene adducts, and (c) stabilized power output (SPO) measurement of the same devices.

To probe the possible perovskite surface passivation effect by the selected indene-fullerene derivatives, we performed absolute photoluminescence measurements (photoluminescence quantum yield, PLQY) of these samples; experimental details are described in the Supporting Information, Section 1.2.5. We applied perovskite thin films, which were deposited directly on a glass substrate, followed by coating different ETMs on top (**PCBM** and newly developed derivatives: **NH**_**2**_**-ICMA**, **NHAc-ICMA**, and **NHAc-Me-ICMA**). In this way, we could compare the amount of non-radiative recombination losses originating at the perovskite/ETL contact. The results are presented in Figure 4a. The bare perovskite layer shows the highest PL intensity. The addition of a fullerene layer partially quenches the signal, primarily due to the increased non-radiative recombination at the perovskite/ETM interface.

In Figure 4a, we provide the summary of the PLQY results, together with the calculated quasi-Fermi level splitting (QFLS) values (corresponding to attainable *V*_OC_ values under given illumination conditions). It is evident that all the studied ETMs, when coated on top of the perovskite layer, decrease its QFLS. The bare perovskite film displayed a PLQY of ∼0.00383% (measured at 0.05 Sun), which corresponds to the QFLS value of 1.16 eV. Compared to the bare perovskite film, the addition of a fullerene layer causes a significant decrease in the photoluminescence efficiency and gives rise to non-radiative recombination losses. The QFLS obtained for **NHAc-ICMA**, **NH**_**2**_**-ICMA**, **NHAc-Me-ICMA**, and **PCBM** samples is 1.13, 1.12, 1.14, and 1.15 eV, respectively. From this, we can infer that in the set of different indene fullerenes, the **NHAc-Me-ICMA** compound displays the lowest amount of interfacial recombination losses. These losses were even lower for the **PCBM** reference sample, with a QFLS of 1.15 eV. We also performed time-resolved photoluminescence (TRPL) decay measurements for the same set of samples (Supporting Information 1.2.6). The curves are shown in Figure S1, Supporting Information. The TRPL signal is affected by charge extraction, charge trapping, and recombination processes.^61^ Due to low-intensity (5 mW/cm^2^) and high-energy photons (405 nm) of the excitation beam (laser goes through the fullerene side), the initial decay is likely to be driven primarily by charge trapping and electron extraction.^61,62^ All the ETLs show comparable decays, with the **PCBM** displaying the fastest and **NH**_**2**_**-ICMA** displaying the slowest early time (first 50 ns) quenching. In view of the highest QFLS for the **PCBM**, this indicates superior electron extraction in this sample. Structural variations in the fullerene derivatives can strongly influence the electronic contact with the perovskite layer, even when the respective energy levels are unchanged. Size effects of the bulky functional groups can also influence intermolecular interactions of the neighboring carbon cages, which in turn could reflect in the reduction of electron mobility values, leading to electron extraction difficulties.^63^

In order to elucidate the relationship between the molecular structure of the indene-fullerene derivatives and their operation as ETMs in PSCs, we fabricated photovoltaic devices of p–i–n configuration; for experimental details and characterization techniques, see the Supporting Information, Sections 1.1 and 1.2.2.^34^ In Table 2, we report average values of the photovoltaic parameters extracted from the current density–voltage (*J*–*V*) characterization measurements; the parameters for the champion devices are given in brackets. The respective *J*–*V* curves of the best devices for each fullerene derivative are shown in Figure 4b. We also measured the spectral response of the representative devices for all the ETM variations. The external quantum efficiency spectra with integrated current density values (similar to the values obtained from the *J*–*V* measurements) are shown in Figure S2, Supporting Information.

**Table 2:** Photovoltaic Parameters Extracted from the Current–Voltage Characterization Measurements of the PSCs Fabricated with Different Fullerene Adducts

fullerene	PCE^avg±SD^ [%]	FF^avg±SD^ [%]	*V*_OC_^avg±SD^ [V]	*J*_SC_^avg±SD^ [mA cm^–2^]	SPO [%]
**NHAc-ICMA**	(13.61) 10.71 ± 2.27	(68.14) 60.07 ± 5.55	(1.00) 0.95 ± 0.04	(20.07) 18.59 ± 0.87	9.97
**NH**_**2**_**-ICMA**	(10.12) 6.99 ± 1.86	(66.10) 53.92 ± 7.08	(0.90) 0.88 ± 0.10	(17.03) 14.58 ± 1.59	2.75
**NHAc-Me-ICMA**	(11.98) 7.64 ± 2.51	(60.01) 47 ± 7.83	(1.02) 0.94 ± 0.06	(19.54) 16.80 ± 2.07	7.75
**PCBM**	(13.07) 9.27 ± 2.27	(72.15) 58.0 ± 8.60	(1.03) 0.97 ± 0.05	(17.63) 16.02 ± 1.56	11.86

**Figure 5:**
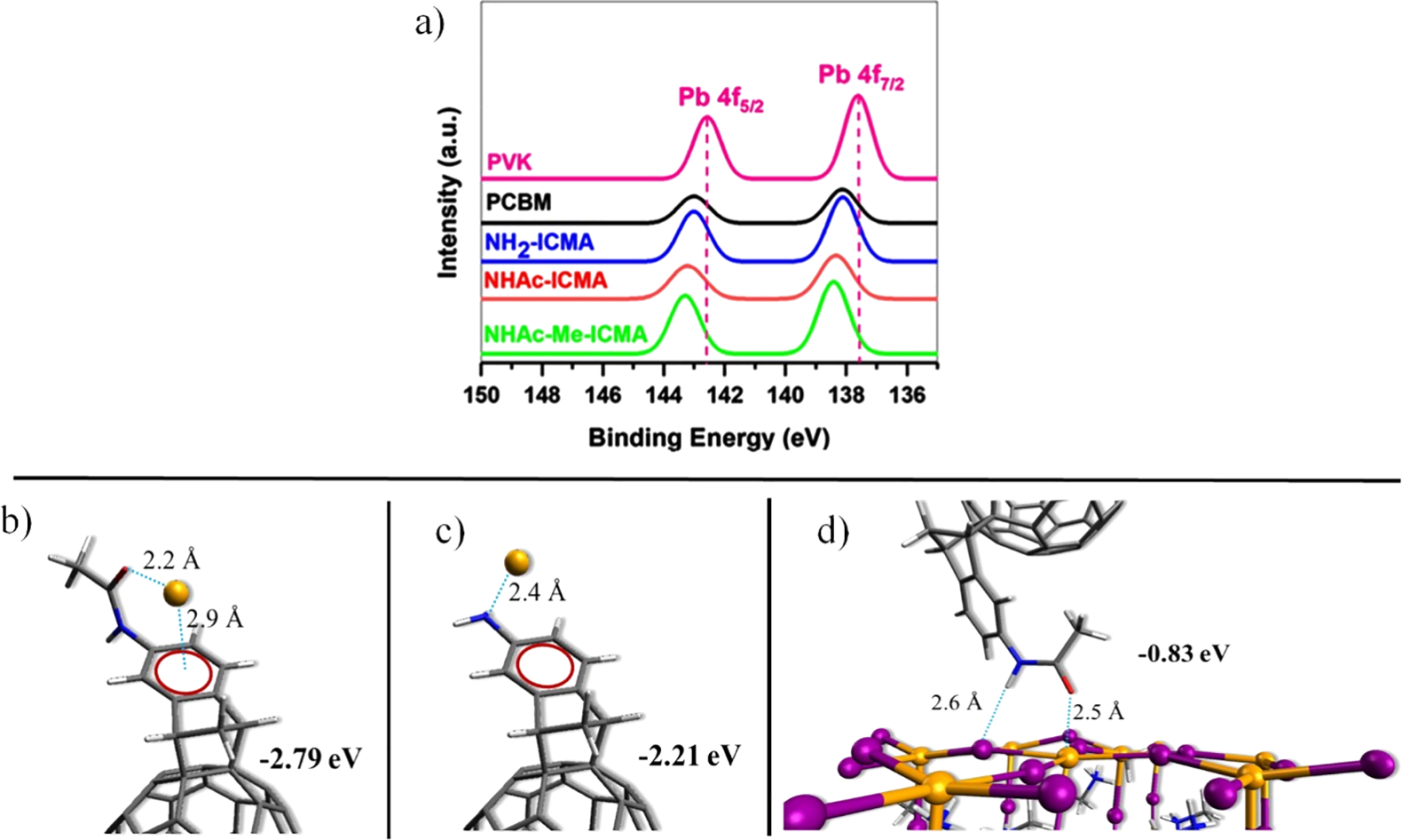
(a) XPS measurements for Pb orbitals. The input X-ray source was Al Kα (1486 eV). Models for the interaction of Pb^2+^ with (b) **NHAc-ICMA**, (c) **NH**_**2**_**-ICMA**, and (d) possible mechanism of passivation of undercoordinated Pb^2+^ by **NHAc-ICMA**. Interaction energy (in eV) and atomic distances (in Å) are reported.

The trend in *V*_OC_ values (Table 2) between different fullerenes is in good agreement with the variations in QFLS values (Figure 4a–c), supporting the previous statement that within the set of indene-fullerene derivatives, the **NHAc-Me-ICMA** cells display reduced recombination at the perovskite/ETL interface. This also points toward the possible passivation effect of the amine group embedded in the **NHAc-Me-ICMA** molecule. To further investigate possible chemical interactions between different functional groups in the used fullerene derivatives and perovskite’s surface, we applied X-ray photoelectron spectroscopy (XPS). The XPS data are shown in Figure 5a. It is evident that the binding energy of the Pb 4f core level of a bare perovskite film shifts toward higher values upon deposition of a thin layer of one of the fullerene derivatives. It implies the presence of a more negative charge around the Pb^2+^ ions. Upon the formation of the perovskite/ICMA contact, electron-donating moieties in the fullerene derivatives (amine or amide groups) could form a dative bond with uncoordinated Pb^2+^ ions.^64^ The largest shift has been observed for the **NHAc-Me-ICMA**, which contains both oxygen and nitrogen donors. The reference **PCBM** displayed a smaller shift, as the coordinating ability of the oxygen atom in its ester group is weaker. The interaction of Pb^2+^ with **NHAc-ICMA** and **NH**_**2**_**-ICMA** adducts was further evaluated by additional DFT calculations (see the Supporting Information, Section 1.5, for the detailed methodology). The interaction energy, obtained by subtracting the energy of the bare Pb^2+^ ion and the energy of the adduct from the total energy of the interacting system, yielded values of −2.79 and −2.21 eV for the **NHAc-ICMA** and **NH**_**2**_**-ICMA** adducts, respectively (see Figure 5b,c). This implies that the amide group can exhibit stronger coordination to Pb^2+^ than the amine moieties, with a possibility of an enhanced passivation effect.^51,52^ The evaluation of the interaction energy between the **NHAc-ICMA** adduct and the perovskite surface resulted in −0.83 eV, thus suggesting a stabilizing interaction between the two materials, as we schematically show in Figure 5d. Therefore, since the electronic properties and the LUMO energies are expected to be very similar for the two adducts (see data in Table 1), we hypothesize that the higher *V*_OC_ values of the **NHAc-ICMA** adduct are related to its ability to bind with the undercoordinated Pb^2+^ and consequentially passivate surface trap states. A stronger steric clash caused by the additional methyl group is the main difference between the **NHAc-ICMA** and **NHAc-Me-ICMA** compounds, which can induce differences in the layer packing and ensuing passivation effects.

Within the cells incorporating the newly synthesized fullerene derivatives, the **NHAc-ICMA** sample delivered the highest PCE, reaching up to 13.61%. The PCE enhancement upon **NHAc-ICMA** incorporation predominantly originated from the increase in *J*_SC_ and FF, yielding 20.07 mA cm^–2^ and 68.14%, respectively. The improved *J*_SC_ for the **NHAc-ICMA**-based cells could originate from the improved ETL morphology and more conformal capping of the perovskite surface, as evidenced by the cross-sectional SEM images in Figure 3d. In the case of the reference device employing solution-processed **PCBM**, we measured a PCE of 13.07% and *J*_SC_ of 17.63 mA cm^–2^. Additionally, we observed that the PSCs with the **NH**_**2**_**-ICMA** molecule delivered a relatively low *J*_SC_. This effect could be attributed to the sub-optimal morphology of this ETL, displaying large aggregates and non-complete perovskite coverage (see Figure 3f). We also note lower solubility of the **NH**_**2**_**-ICMA** compound in the *o*-DCB, which could result in a higher aggregation tendency and, in turn, less-uniform film morphologies.^24^ This is also evidenced by AFM images (high surface roughness, as shown in Figure 3). A smooth and uniform ETL morphology is needed to provide an intimate contact with the perovskite film, which, in turn, leads to effective charge extraction and high current densities.

We also performed SPO measurements under continuous illumination. The SPO efficiency decreased over time for the devices with the indene fullerenes, as shown in Figure 4c and Table 2. Additionally, for these cells, we recorded larger differences between *JV*-derived and SPO-derived PCEs than for the reference, **PCBM**-based samples. This could be related to the non-optimal electron extraction efficacy (space charge region forming at the interface at lower electric fields), which can be influenced by lower electron mobilities in the newly synthesized ETMs.^65^ More detailed photophysical characterization of the interface between the given ETM and perovskite is needed for thorough understanding of the origin of these effects.

## Conclusions

In summary, we have developed a series of new fullerene derivatives and have presented the synthesis of the indene-fullerene adducts, and carried it out by appending different indene derivatives to the fullerene C_60_ through the Diels–Alder cycloaddition process. This synthetic methodology provides a novel approach to obtain a wide spectrum of indene-fullerene adducts, which are inaccessible by previous, conventional pathways. We also performed computational simulations and CV measurements, the results of which indicate that groups with a stronger electron donation effect in the indene structure are the key motif in the indene-fullerene adduct toward its application as an ETL. Based on these results, we fabricated flexible PSCs incorporating indene-fullerene derivatives with the amine and amide groups as ETLs. We tested the **NH**_**2**_**-ICMA** compound and its derivatives, the **NHAc-ICMA** and **NHAc-Me-ICMA**, applied to devices with large active areas of 1 cm^2^. We have also provided an insight into the characteristics of the perovskite/ETL interface *via* spectroscopic methods. Notably, the **NHAc-ICMA** and **NHAc-Me-ICMA** derivatives resulted in a decrease in non-radiative recombination losses when compared with the **NH**_**2**_**-ICMA** compound. The **NHAc-ICMA**-based devices showed the best photovoltaic performance, 13.61% of PCE. We believe that further optimization of the device-processing protocol could result in further improvements of the cells performance values.

## Supporting Information Available

Full experimental data and spectroscopic characterizations (PDF)
