# Supplementary material for: Designing New Indene-Fullerene Derivatives as Electron-Transporting Materials for Flexible Perovskite Solar Cells
Source: J Phys Chem C Nanomater Interfaces. 2021 Dec 3;125(49):27344–53. doi: 10.1021/acs.jpcc.1c07189 (PMC8802170; doi:10.1021/acs.jpcc.1c07189)
Supplement: Supplementary file 1 — jp1c07189_si_001.pdf [file jp1c07189_si_001.pdf]

Supporting Information

Designing New Indene-Fullerene Derivatives as Electron-Transporting Materials for Flexible Perovskite Solar Cells

Lukasz Przypis,†*[1,2] Taimoor Ahmad,†[3,4] Kasjan Misztal,[1] Damian Honisz,[5] Eros Radicchi,[6,7] Edoardo Mosconi,[6] Wojciech Domagala,[5] Filippo De Angelis,[6,7,8,9] Konrad Wojciechowski*[1,3]

[1] Saule Research Institute, Wroclaw Technology Park, 11 Dunska street, Sigma Building, 54-130 Wrocław, Poland
[2] Silesian University of Technology, Department of Organic Chemistry, Bioorganic Chemistry and Biotechnology, Bolesława Krzywoustego 4, 44-100 Gliwice, Poland
[3] Saule Technologies Ltd., Wroclaw Technology Park, 11 Dunska street, Sigma Building, 54-130 Wrocław, Poland
[4] University of Rome "Tor Vergata", Department of Electronics Engineering, Via del Politecnico 1, 00133 Rome, Italy
[5] Faculty of Chemistry, Silesian University of Technology, Marcina Strzody 9, Gliwice 44-100, Poland
[6] Computational Laboratory for Hybrid/Organic Photovoltaics (CLHYO), Istituto CNR di Scienze e Tecnologie Chimiche "Giulio Natta" (CNR-SCITEC), Via Elce di Sotto 8, Perugia 06123, Italy
[7] Department of Chemistry, Biology and Biotechnology, University of Perugia, Via Elce di Sotto 8, Perugia 06123, Italy
[8] CompuNet, Istituto Italiano di Tecnologia, Via Morego 30, Genova 16163, Italy
[9] Department of Mechanical Engineering, College of Engineering, Prince Mohammad Bin Fahd University, P.O. Box 1664 Al Khobar 31952, Kingdom of Saudi Arabia
†These authors made equal contributions to the work
* Corresponding author
E-mail addresses: **Lukasz.Przypis@sauleresearch.com; Konrad.Wojciechowski@sauletech.com**

1. EXPERIMENTAL SECTION

1.1. Materials and device fabrication

Commercially available chemicals were used without further purification. Grubbs II catalyst and nitro-Hoveyda−Grubbs catalyst were purchased from commercial suppliers. The boronic acid pinacol esters were bought from a chemical supplier or synthesized from the corresponding bromides based on literature reports. Polyethylene terephthalate (PET) flexible substrates coated with indium doped zinc oxide (IZO, sheet resistance of 15 Ω/□) was purchased from Eastman chemical company. Lead iodide (PbI_2 99.999%), lead bromide ($PbBr_2$ 98%), cesium iodide (CsI 98%), N,N-dimethylformamide (DMF), dimethyl sulfoxide (DMSO), toluene, ethyl acetate were precured from Sigma-Aldrich. Formamidinium iodide (FAI) was purchased from Ajay North America, Methylammonium bromide (MABr) was synthesised in house. Poly[bis(4-phenyl)(2,4,6-trimethylphenyl)aminePoly(triarylamine) (PTAA) was purchase from Ossila. All materials were used as received without further purification.

Planar heterojunction flexible perovskite solar cells were fabricated with the following inverted architecture: PET/IZO/PTAA/$Cs_{0.04}(MA_{0.17}FA_{0.83})_{0.96}Pb(I_{0.83}Br_{0.17})_3$/indene-fullerene derivatives/BCP/Ag. PET/IZO (15 Ω/□) substrates were patterned by dipping one side in an HCl solution. The etched substrates were sonicated in deionized water and isopropanol for 6 minutes, followed by nitrogen drying, and 30 seconds of oxygen plasma treatment. A PTAA solution was prepared by dissolving 2 mg of PTAA powder in 1 mL of Toluene and deposited via spin-coating at 5000rpm for 30 seconds, followed by 10 minutes of annealing at 100°C. Subsequently, the samples were transferred into a nitrogen-filled glovebox for the perovskite layer deposition. The perovskite precursor solution composed of mixed cations and halides $Cs_{0.04}(MA_{0.17}FA_{0.83})_{0.96}Pb(I_{0.83}Br_{0.17})_3)$, was prepared according to the procedure reported in our previous report.[1] The perovskite layer was deposited by two-step spinning program (650 rpm for 2 seconds, 4500 rpm for 2 seconds and 4500 rpm for 34 seconds). Solvent quenching was performed with an anhydrous ethyl acetate (150 μL) dispensing on the sample at the 7th second before the end of the spinning sequence. Then, the sample was kept at rest for one minute before transferring to the hotplate for the two-step annealing process, 1 minute at 50 °C and 60

minutes at 100 °C. For the deposition of the electron transporting layers via spin coating, we used indene-fullerenes derivatives. Indene-fullerene solutions (**NHAc-ICMA, NHAc-Me-ICMA, NH_2-ICMA**) were processed from a ortho-dichlorobenzene solution with the concentration of 10 mg/ml, spinning conditions at 2500 rpm for 30 seconds, followed by 15 minutes annealing at 60°C. Finally, 5 nm of BCP buffer layer and 100 nm of Ag electrode were deposited by thermal evaporation, through a shadow mask. The electron mobility from indene fullerenes was investigated using space-charge limited current (SCLC) techniques and an electron-only device configuration of Glass/ITO/TiOx/ETL (different)/TiOx/Ag. We derived the electron mobility by the employing the Mott-Gurney Law.[7]

1.2. Characterization Techniques

1.2.1. Cyclic Voltammetry

The experiments were carried out in a glass cell with a three-electrode setup: a platinum wire as a working electrode, a platinum coil as a counter electrode, and a silver wire as a pseudo-reference electrode. All samples were dissolved (0.5 mM concentration for 7e, 1 mM for the remaining samples) in 0.05 M tetrabutylammonium hexafluorophosphate (Bu_4NPF_6) (TCI Europe) electrolyte solution in 1,2-dichlorobenzene (*o*-DCB) (Acros Organics, 98+%, Extra Dry). All potentials were calibrated relative to ferrocene redox couple (Fc/Fc^+) internal standard. Experiments were performed using Metrohm Autolab PGSTAT 100N potentiostat.

1.2.2. Current voltage measurements

Current density–voltage and stabilized power output characterization, we used an AAA-rated solar simulator (Abet Technologies, sun 2000), calibrated against an RR-208-KG5 silicon reference cell (Abet Technologies). Solar simulation was connected to a Keithley 2461 source measure unit (SMU). For the J-V measurement the solar cells were masked to 1 cm^2 and the scanning rate was set to 500 mV s^{-1}. The stabilized power conversion efficiency (SPO) was measured at the maximum power point voltage for a duration of 30 seconds.

1.2.3. Atomic force microscopy

The AFM characterization was performed by using a Park Systems AFM microscope, Model XE7 in non-contact mode and scanning over a range of 25 µm by 25 µm at a resolution of 128x128 data points. We determined the surface roughness over the scanning area as the root mean-squared roughness. For each sample we took measurements at three different points: for **NHAc-ICMA** sample, we measured the root mean square roughness (RMS) values of 5.4nm, 9.2 nm, 9.7 nm, and 15.6nm. **NH_2-ICMA** samples showed the RMS values of 99.6nm, 82.4nm, 143.7nm and 571.9nm. RMS measurements for **NHAc-Me-ICMA** sample are 11.3nm, 12.5nm and 19.3nm. For the reference **PCBM** sample we measured RMS values of 23.5nm, 26.4nm, 30.0nm, and 36.1nm.

1.2.4. Scanning Electron Microscopy

Cross-section images were obtained by employing focus ion beam scanning electron microscope (FEI Helios 600), with an accelerating voltage of 2 kV. The samples were prepared by depositing carbon and platinum films on top of a sample.

1.2.5. Photoluminescence quantum yield (PLQY)

For the PLQY measurements, the perovskite films were deposited directly on pre-cleaned plain glass substrates. Subsequently, different electron transporting materials were deposited on top of perovskite layer. The measuring setup was based on FS5 Spectrofluorometer (Edinburgh Instruments), equipped with an integrating sphere. An excitation wavelength of 405 nm was used with the intensity of the order of 0.05 sun (5 mW·cm^2). This means that the PLQY and QFLS values are lower than those expected at 1 sun. The PL signal is collected in the spectral range of 720-820 nm.

From the experimentally determined PLQY values, we calculated the quasi-Fermi level splitting (QFLS), using Equation 1:[2]

$$QFLS = QFLS_{rad} + kTln(PLQY) \quad (1)$$

where $QFLS_{rad}$ is the quasi-Fermi level splitting in the radiative limit. This approximation of the QFLS can be related to the achievable upper limit of the open-circuit voltage (V_{OC}) of a respective solar cell.[3–5] Moreover, the QFLS measurements of different layer configurations allowed us to disentangle the influence of the non-radiative recombination in the bulk perovskite film from the losses at the perovskite/ETL interface.[6]

We would like to point out that the PLQY measurements were carried out at a significantly lower light intensity (excitation wavelength: 405 nm, intensity: 5 mW/cm^2) than the current density–voltage J-V characteristics (100 mW/cm^2), due to limitations of the experimental setup (low power of the laser diodes used in the PLQY setup).

1.2.6 Time resolved photoluminescence (TRPL)

The time-resolved photoluminescence (TRPL) spectra were obtained on FS5 Spectrofluorometer (Edinburgh Instruments), equipped with 405ns laser with intensity (5 mW/cm^2). The samples were excited with laser beam through the fullerene side.

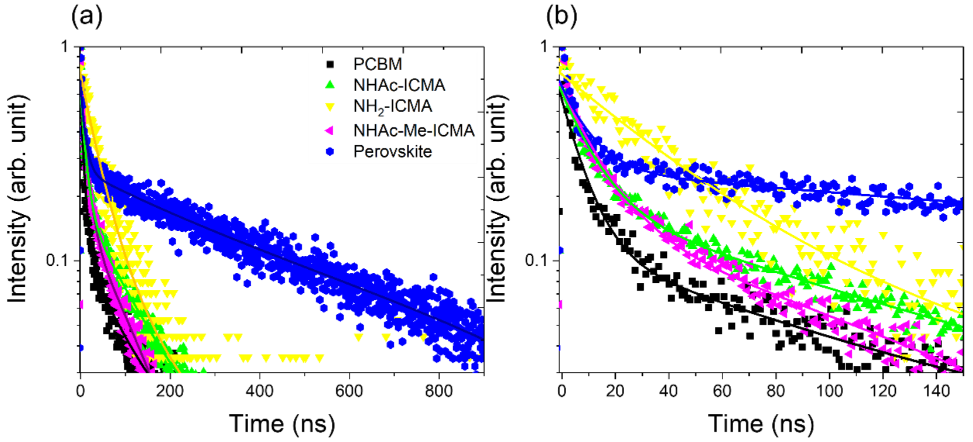

Figure S1. *(a) TRPL spectra with an excitation intensity of bare perovskite layer, and different ETL deposited on top of perovskite, while (b) showing the differences in the early decay times.*

1.2.6.2. EQE measurements

The external quantum efficiency (EQE) was measured using Bentham PVE300 photovoltaic characterization system and the control software BenWin+.

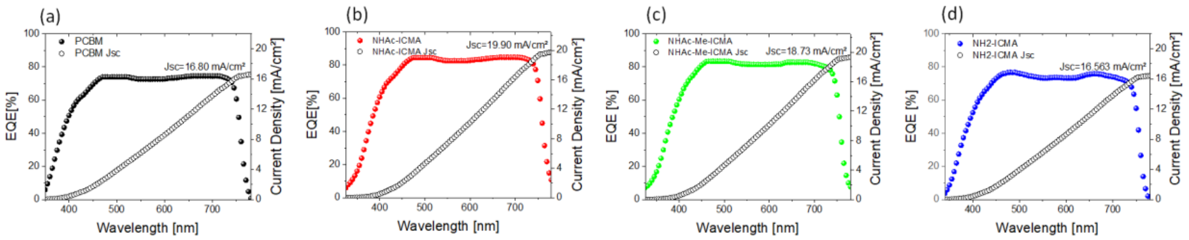

Figure S2. *EQE spectra of NHAc-ICMA, NHAc-Me-ICMA, NH_2-ICMA and PCBM thin films*

1.2.7 Conductivity measurement

The ETL conductivity was measured between the two ITO stripes, which were formed by laser ablating a100 μm wide trench. The length of each electrode finger is 5 mm.

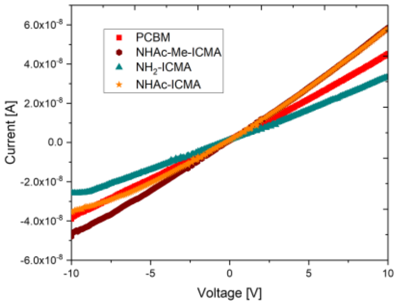

Figure S3. *Current-voltage (J-V) characteristics of NHAc-ICMA, NHAc-Me-ICMA, NH_2-ICMA and PCBM thin films*

1.2.8 Theoretical methodology

Density Functional Theory (DFT) simulations were carried out with the Gaussian 09 software,[8] employing the B3LYP exchange-correlation functional and ortho-dichlorobenzene (*o*-DCB) as the implicit solvent within the polarizable continuum model (PCM).[9–11]

For the estimation of molecular orbitals, we optimized adducts geometries with 6-31G* basis sets, which we then used to run single point calculations with high quality 6-311++G** basis sets. The same approach was adopted for the evaluation of electron affinity (EA) and first ionization potential (IP). On the other hand, for the simulation of the Pb^{2+} - adducts interaction we recurred to the 6-31G* (for C, H, N, O) and LANL2DZ (for Pb) basis sets, together with the LANL2 pseudopotential (for Pb). In this last case, we also employed Grimme van der Waals correction (DFT-D3) to take into account dispersion interactions.[12]

1.3. SYNTHESIS OF INDENE-FULLERENE ADDUCTS

All reactions were carried out under an argon atmosphere in oven-dried glassware with magnetic stirring. Analytical thin-layer chromatography (TLC) was performed on silica gel 60 TLC plates. The spots were visualized with a UV light or by staining with $KMnO_4$ or anisaldehyde solution. Flash column chromatography was performed using silica gel 60 (particle size 0.040–0.063 mm), typically using an n-hexane/ethyl acetate eluent system and toluene/ethyl acetate in case of fullerene derivatives. A usual workup of the reaction mixture consists of extraction with ether or ethyl acetate, washing with water, brine, drying over Na_2SO_4, and then concentration under reduced pressure on a rotary evaporator unless specified. Reported yields are based upon isolation following purification by silica gel column chromatography; isolated materials were judged to be homogeneous based upon TLC and NMR. NMR spectra were measured at room temperature on a Bruker 400 MHz spectrometer or Bruker AV II 600 MHz. NMR spectra were calibrated to the solvent residual signals of $CDCl_3$. ^1H NMR spectra were recorded at 400 MHz. Data are reported as follows: chemical shift, multiplicity (s: singlet, d: doublet, t: triplet, q: quartet, qui: quintuplet, m: multiplet), coupling constant (J in Hz), and integration. ^{13}C NMR spectra were recorded at 100 or 150 MHz using broadband proton decoupling, and chemicals shifts are reported in ppm using residual solvent peaks as reference. High resolution mass spectra were recorded on an MS (ESI) spectra LCMS-IT TOF Shimadzu or SYNAPT G2-S HDMS (Waters). Low resolution mass spectra were recorded at Thermo Scientific ISQ 7000.

1.3.1. General procedure for synthesis of allyloxy derivative 2a-2d

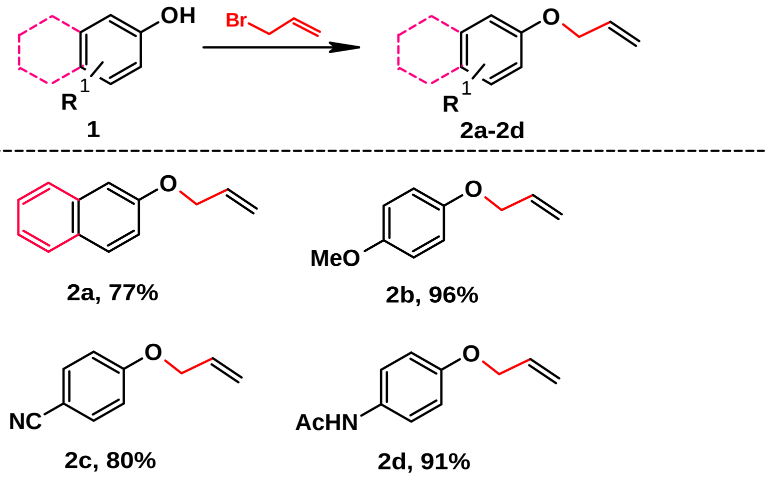

Phenol (1 equiv) was dissolved in dry DMF (c=0.3 M) at room temperature under an argon atmosphere. Anhydrous potassium carbonate (2.5 equiv) was added and mixture was stirred for 10 min. Then allyl bromide (1.5 equiv) was added at once and reaction was stirred overnight at 80 °C. When all starting reagent was consumed reaction was diluted with AcOEt and washed three times with saturated aqueous solution of NH_4Cl, once with brine, dried with $MgSO_4$, filtrated and concentrated.

2-(allyloxy)naphthalene (2a)

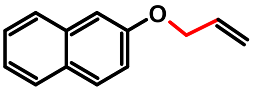

66 mmol scale; brown solid, 9.33 g, 77%; **^{1}H NMR (400 MHz, $CDCl_3$)** δ 7.75 (dt, J = 8.7, 4.2 Hz, 3H), 7.45 (ddd, J = 8.2, 7.0, 1.2 Hz, 1H), 7.35 (ddd, J = 8.1, 7.0, 1.1 Hz, 1H), 7.22 – 7.14 (m, 2H), 6.22 – 6.04 (m, 1H), 5.49 (dq, J = 17.3, 1.6 Hz, 1H), 5.38 – 5.30 (m, 1H), 4.67 (ddd, J = 5.3, 2.2, 0.8 Hz, 2H).

^{13}C NMR (101 MHz, $CDCl_3$) δ 156.61, 134.60, 133.28, 129.52, 129.11, 127.75, 126.86, 126.45, 123.75, 119.07, 117.90, 107.08, 68.91.

1-(allyloxy)-4-methoxybenzene (2b)

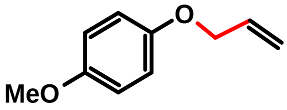

33 mmol scale; yellow liquid, 5.22 g, 96%; **^{1}H NMR (400 MHz, $CDCl_3$)** δ 6.95 – 6.79 (m, 4H), 6.08 (ddt, *J* = 17.2, 10.6, 5.3 Hz, 1H), 5.43 (ddd, *J* = 17.3, 3.2, 1.6 Hz, 1H), 5.30 (ddd, *J* = 10.5, 2.8, 1.4 Hz, 1H), 4.52 (dt, *J* = 5.3, 1.5 Hz, 2H), 3.80 (s, 3H).

^{13}C NMR (101 MHz, $CDCl_3$) δ 153.29, 151.01, 131.97, 130.93, 118.42, 106.05, 70.05, 56.12.

4-(allyloxy)benzonitrile (2c)

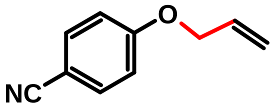

66 mmol scale; yellow solid, 8.38 g, 80%; **^{1}H NMR (400 MHz, $CDCl_3$)** δ 7.61 – 7.51 (m, 2H), 6.99 – 6.89 (m, 2H), 6.09 – 5.92 (m, 1H), 5.36 (ddd, *J* = 13.9, 11.9, 1.4 Hz, 2H), 4.57 (dt, *J* = 5.3, 1.5 Hz, 2H).

^{13}C NMR (101 MHz, $CDCl_3$) δ 161.91, 134.06, 134.05, 132.14, 119.28, 118.59, 115.53, 115.53, 104.16, 69.08.

1-allyloxy-4-acetamidophenyl (2d)

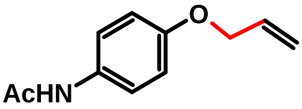

33 mmol scale; brown solid (5.75 g, 91%); **^{1}H NMR (400 MHz, $CDCl_3$)** δ 8.01 (s, 1H), 7.45 (s, 1H), 7.42 – 7.34 (m, 2H), 6.88 – 6.81 (m, 2H), 6.13 – 5.95 (m, 1H), 5.39 (dd, J = 17.3, 1.6 Hz, 1H), 5.27 (dd, J = 10.5, 1.4 Hz, 1H), 4.50 (dt, J = 5.3, 1.5 Hz, 2H), 2.95 (d, J = 0.4 Hz, 2H), 2.88 (d, J = 0.6 Hz, 2H), 2.13 (d, J = 3.6 Hz, 3H).

^{13}C NMR (101 MHz, $CDCl_3$) δ 168.38, 155.50, 133.32, 131.23, 121.95, 117.78, 115.11, 77.44, 77.12, 76.80, 69.16, 24.41.

1.3.2. General procedure for synthesis of *orto*-allylphenol derivatives 3a-3d

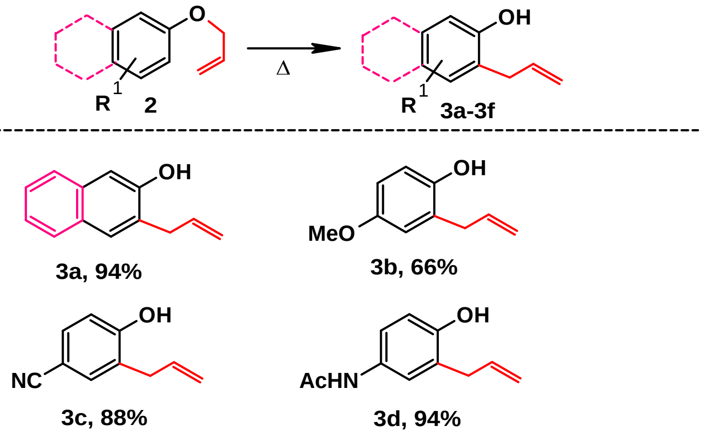

Allyloxy (1 equiv) derivative was placed in a flask containing diphenyl ether (1 equiv) and was stirred for 6 h at 190 °C. After this time reaction mixture was cooled to room temperature, taken into 1M HCl and extracted three times with diethyl ether (3x50 mL), then pH was adjusted to basic (pH 8-9) and extracted again three times with diethyl ether (3x50 mL). Drying with $MgSO_4$ and evaporation of organic phase afforded phenol.

1-allylnaphthalen-2-ol (3a)

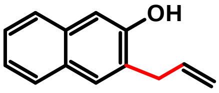

46 mmol scale; orange solid, 8.00 g, 94%; **^{1}H NMR (400 MHz, $CDCl_3$)** δ 7.91 (d, J = 8.6 Hz, 1H), 7.79 (d, J = 8.1 Hz, 1H), 7.68 (d, J = 8.8 Hz, 1H), 7.49 (ddd, J = 8.4, 6.8, 1.3 Hz, 1H), 7.37 – 7.31 (m, 1H), 7.11 (d, J = 8.8 Hz, 1H), 6.18 – 5.99 (m, 1H), 5.18 – 5.02 (m, 3H), 3.84 (dt, J = 5.8, 1.7 Hz, 2H).

^{13}C NMR (101 MHz, $CDCl_3$) δ 151.32, 135.85, 133.34, 129.53, 128.67, 128.43, 126.59, 123.26, 123.10, 118.04, 116.90, 116.05, 31.06.

2-allyl-4-methoxyphenol (3b)

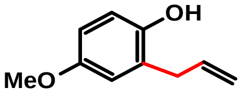

18 mmol scale; yellow solid, 2.0 g, 66%; **^{1}H NMR (400 MHz, $CDCl_3$)** δ 6.82 – 6.60 (m, 3H), 6.14 – 5.86 (m, 1H), 5.23 – 5.06 (m, 2H), 4.60 (s, 1H), 3.76 (d, *J* = 1.0 Hz, 3H), 3.38 (d, *J* = 6.3 Hz, 2H).

^{13}C NMR (101 MHz, $CDCl_3$) δ 153.67, 147.99, 136.24, 126.75, 116.46 (2C), 115.99, 112.63, 55.79, 35.15.

3-allyl-4-hydroxybenzonitrile (3c)

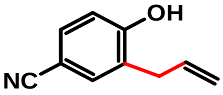

25 mmol scale; yellow solid, 3.50 g, 88%; **^{1}H NMR (400 MHz, $CDCl_3$)** δ 7.48 – 7.38 (m, 2H), 6.94 – 6.81 (m, 1H), 6.30 (s, 1H), 5.97 (ddt, *J* = 16.6, 10.1, 6.5 Hz, 1H), 5.18 (ddq, *J* = 18.7, 17.1, 1.5 Hz, 2H), 3.41 (d, *J* = 6.5 Hz, 2H).

^{13}C NMR (101 MHz, $CDCl_3$) δ 158.41, 134.97, 134.51, 132.41, 127.42, 119.43, 117.63, 116.45, 103.55, 34.29.

2-allyl-4-acetamidophenol (3d)

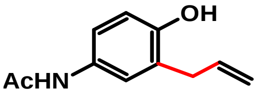

30 mmol scale; brown solid, 5.4 g, 94%; **^{1}H NMR (400 MHz, acetone-d_6)** δ 8.93 (s, 1H), 8.22 (s, 1H), 7.33 (dd, *J* = 8.6, 2.6 Hz, 1H), 7.27 (s, 1H), 6.73 (d, *J* = 8.6 Hz, 1H), 5.95 (ddt, *J* = 16.8, 10.1, 6.7 Hz, 1H), 5.08 – 4.92 (m, 2H), 3.30 (d, *J* = 6.7 Hz, 2H), 2.00 (s, 3H).

^{13}C NMR (101 MHz, acetone-d_6) δ 205.52, 167.48, 150.84, 137.02, 131.79, 126.31, 121.29, 118.56, 114.65, 114.62, 23.13.

1.3.3. General procedure for synthesis of triflates 4a-4d

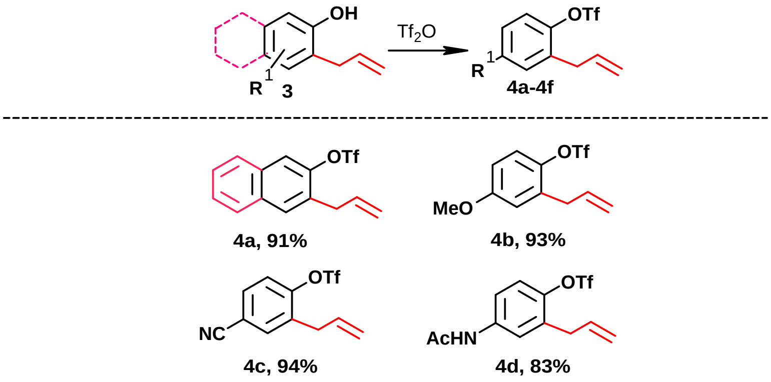

Reactions were carried on an 8.76 mmol scale unless otherwise noted. The substituted phenol (12 mmol, 1 equiv) was solubilized in a mixture of 7 mL of pyridine and 7 mL of dry dichloromethane at 0 °C under an argon atmosphere. To the resulting mixture was dropwise added neat trifluoromethanesulfonyl anhydride (13.23 mmol, 1.1 equiv). The reaction mixture was stirred and allowed to reach room temperature overnight. After this time, the solvents were removed by rotary evaporation and the remaining residue was taken up in ethyl acetate (100 mL) and extracted with 5%

HCl (3 × 50 mL), followed by sodium bicarbonate (2 × 50 mL), brine (100 mL), and drying over $MgSO_4$. Residue was concentrated in vacuum and purified with column chromatography to obtain the corresponding triflate.

1-allylnaphthalen-2-yl trifluoromethanesulfonate (4a)

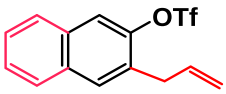

7 mmol scale; brown solid, 2.01 g, 91%; **^{1}H NMR (400 MHz, $CDCl_3$)** δ 8.06 (dd, *J* = 8.4, 0.8 Hz, 1H), 7.88 (dd, *J* = 8.3, 1.1 Hz, 1H), 7.81 (d, *J* = 9.1 Hz, 1H), 7.64 – 7.51 (m, 2H), 7.39 (d, *J* = 9.1 Hz, 1H), 6.01 (ddt, *J* = 17.1, 10.2, 5.8 Hz, 1H), 5.11 (dq, *J* = 10.2, 1.6 Hz, 1H), 5.02 (dq, *J* = 17.1, 1.8 Hz, 1H), 3.93 (dt, *J* = 5.8, 1.8 Hz, 2H).

^{13}C NMR (101 MHz, $CDCl_3$) δ 157.32, 145.28, 134.52, 132.79, 129.81, 129.32, 128.81, 127.49, 126.81, 125.06, 119.47, 118.97, 117.10, 30.27.

2-allyl-4-methoxyphenyl trifluoromethanesulfonate (4b)

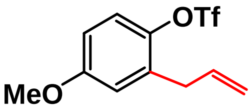

12 mmol scale; colorless liquid, 3.37 g, 93%; **^{1}H NMR (400 MHz, $CDCl_3$)** δ 7.16 (d, *J* = 8.9 Hz, 1H), 6.78 (dt, *J* = 9.0, 3.1 Hz, 2H), 5.89 (ddt, *J* = 16.8, 10.2, 6.6 Hz, 1H), 5.14 (ddq, *J* = 17.0, 12.9, 1.4 Hz, 2H), 3.79 (s, 3H), 3.43 (d, *J* = 6.6 Hz, 2H).

^{13}C NMR (101 MHz, $CDCl_3$) δ 159.01, 141.42, 134.52, 122.38, 120.27, 117.74, 117.08, 116.24, 112.92, 55.72, 34.30.

2-allyl-4-cyanophenyl trifluoromethanesulfonate (4c)

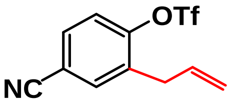

21 mmol scale; orange liquid, 5.76 g, 93%; **^{1}H NMR (400 MHz, $CDCl_3$)** δ 7.68 – 7.55 (m, 2H), 7.40 (d, *J* = 8.5 Hz, 1H), 5.87 (ddt, *J* = 16.8, 10.1, 6.6 Hz, 1H), 5.25 (dq, *J* = 10.1, 1.3 Hz, 1H), 5.16 (dq, *J* = 17.0, 1.5 Hz, 1H), 3.49 (d, *J* = 6.6 Hz, 2H).

^{13}C NMR (101 MHz, $CDCl_3$) δ 150.39, 135.38, 134.98, 133.00, 132.17, 122.60, 122.60, 119.20, 117.36, 112.86, 33.68.

4-Acetamido-2-allylphenyl trifluoromethanesulfonate (4d)

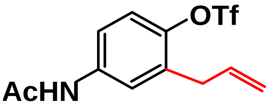

(on a scale 12 mmol): orange crystals, 3.22 g, 83%; **^{1}H NMR (400 MHz, CDCl3)** δ 7.58 – 7.44 (m, 2H), 7.35 (s, 2H), 7.20 (d, J = 8.9 Hz, 1H), 5.89 (ddt, J = 16.8, 10.2, 6.6 Hz, 1H), 5.21 – 5.07 (m, 2H), 3.44 (d, J = 6.6 Hz, 2H), 2.17 (s, 3H).

^{13}C NMR (101 MHz, CDCl3) δ 168.32, 143.61, 137.82, 134.24, 133.79, 121.96, 121.90, 119.02, 117.80, 34.04, 30.89, 24.53.

1.3.4. General Procedure for Suzuki Coupling 5a-5e

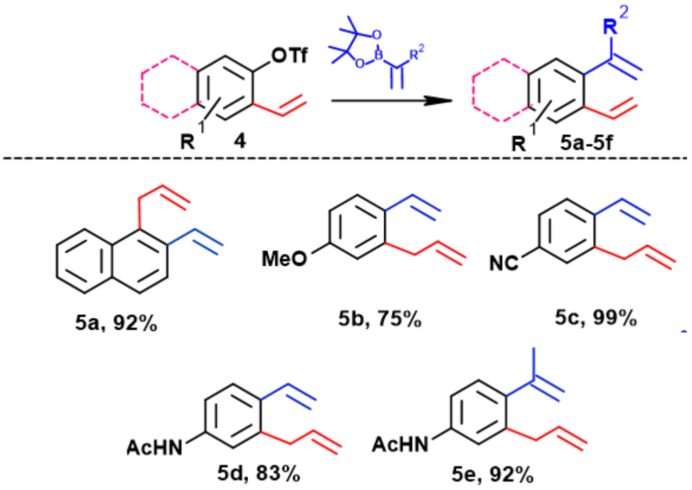

Reactions were carried on a 1 mmol scale unless otherwise noted. A Schlenk flask was charged with triflate (1 mmol), vinyl boronic ester (1.3 mmol), and K_2CO_3 (3 mmol). Then, 10 mL of a 4:1 1,4-dioxane:water mixture was added and the flask was deoxygenated by freeze−pump−thaw cycle three times. [Pd(PPh3)4] (0.10 mmol) was added, and the sealed flask was stirred at 120°C for 18 h (overnight), resulting in usually orange, transparent suspension. After that time, the flask was cooled to RT and all volatiles were removed under reduced pressure. The oily residue was solubilized in AcOEt and filtered through a Celite pad. Volatiles were concentrated and separated with column chromatography to obtain diene.

1-allyl-2-vinylnaphthalene (5a)

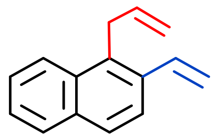

0.25 mmol scale, orange solid, 92%[1;] **^{1}H NMR (600 MHz, CDCl$_3$)** δ 8.04 (d, *J* = 8.5 Hz, 1H), 7.82 (dd, *J*= 6.0, 3.3 Hz, 1H), 7.70 (dd, *J* = 24.4, 8.6 Hz, 2H), 7.47 (dddd, *J* = 20.6, 7.9, 6.8, 1.3 Hz, 2H), 7.20 (dd, *J* = 17.4, 11.0 Hz, 1H), 6.08 (ddt, *J* = 17.1, 10.2, 5.6 Hz, 1H), 5.78 (dd, *J* = 17.4, 1.2 Hz, 1H), 5.43 (dd, *J* = 11.0, 1.2 Hz, 1H), 5.06 (dq, *J* = 10.2, 1.7 Hz, 1H), 4.93 (dq, *J* = 17.1, 1.8 Hz, 1H), 3.93 (dt, *J* = 5.5, 1.8 Hz, 2H).

^{13}C NMR (150 MHz, CDCl$_3$) δ 136.15, 135.22, 133.53, 132.51, 129.84, 128.61, 127.13, 126.34, 125.55, 124.61, 124.07, 118.99, 116.52, 116.03, 32.24.

EI-MS *m/z*: $[M]^+$ Calcd for $C_{15}H_{14}$ 194.28; Found: 194.1

2-allyl-4-methoxy-1-vinylbenzene (5b)

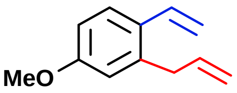

0.30 mmol scale, orange solid, 75%; **^{1}H NMR (600 MHz, CDCl$_3$)** δ 7.49 (d, *J* = 8.6 Hz, 1H), 6.92 (dd, *J* = 17.4, 10.9 Hz, 1H), 6.83 – 6.67 (m, 2H), 6.06 – 5.88 (m, 1H), 5.57 (dd, *J* = 17.4, 1.4 Hz, 1H), 5.21 (dd, *J* = 10.9, 1.4 Hz, 1H), 5.07 (ddq, *J* = 45.0, 17.1, 1.6 Hz, 2H), 3.84 (s, 3H), 3.52 – 3.36 (m, 2H).

^{13}C NMR (151 MHz, CDCl$_3$) δ 159.34, 138.60, 136.61, 133.94, 129.51, 126.89, 116.01, 114.90, 113.54, 112.11, 55.24, 37.61.

EI-MS *m/z*: $[M]^+$ Calcd for $C_{12}H_{14}O$ 174.24; Found: 174.1

3-allyl-4-vinylbenzonitrile (5c)

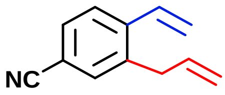

0.25 mmol scale, orange oil, 99%; **^{1}H NMR (600 MHz, CDCl$_3$)** δ 7.56 (d, *J* = 8.1 Hz, 1H), 7.51 – 7.41 (m, 2H), 6.90 (ddd, *J* = 20.9, 17.4, 11.0 Hz, 1H), 5.99 – 5.83 (m, 1H), 5.74 (dd, *J* = 17.4, 0.9 Hz, 1H), 5.48 – 5.40 (m, 1H), 5.19 – 5.08 (m, 1H), 5.07 – 4.91 (m, 1H), 3.53 – 3.31 (m, 2H).

^{13}C NMR (150 MHz, CDCl$_3$) δ 141.50, 138.30, 135.29, 133.40, 130.29, 126.51, 119.02, 117.24, 114.97, 112.17, 111.31, 36.99.

EI-MS *m/z*: $[M]^+$ Calcd for $C_{12}H_{11}N$ 169.23; Found: 169.1

***N*-(3-allyl-4-vinylphenyl)acetamide (5d)**

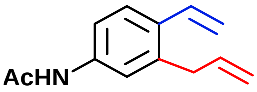

orange solid, 2.75 mmol scale, 458 mg, 83%; **^{1}H NMR (400 MHz, CDCl$_3$)** δ 7.77 (s, 1H), 7.30 (d, J = 8.1 Hz, 1H), 7.22 (dd, J = 8.0, 1.5 Hz, 1H), 6.82 (d, J = 5.5 Hz, 1H), 6.57 – 6.40 (m, 1H), 3.38 (s, 2H), 2.17 (s, 3H).

^{13}C NMR (101 MHz, CDCl$_3$) δ 168.28, 144.85, 141.50, 134.96, 133.95, 131.58, 120.93, 118.38, 116.44, 39.30, 29.78, 24.72.

EI-MS *m/z*: $[M]^+$ Calcd for $C_{13}H_{15}NO$ 201.27; Found: 201.1

***N*-(3-allyl-4-(prop-1-en-2-yl)phenyl)acetamide (5e)**

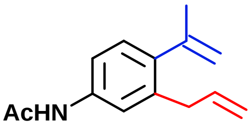

orange solid, 2.75 mmol scale, 544 mg, 92%; **^{1}H NMR (400 MHz, CDCl$_3$)** δ 7.39 (dd, J = 8.2, 2.2 Hz, 1H), 7.23 (d, J = 2.2 Hz, 1H), 7.10 (t, J = 9.0 Hz, 2H), 5.92 (ddt, J = 16.7, 10.2, 6.5 Hz, 1H), 5.20 (m, 1H), 5.03 (m, 2H), 4.83 (dd, J = 2.0, 0.9 Hz, 1H), 3.39 (dt, J = 6.4, 1.3 Hz, 2H), 2.16 (s, 3H), 2.00 (m, 3H).

^{13}C NMR (101 MHz, CDCl$_3$) δ 168.62, 144.90, 139.87, 137.77, 136.82, 132.20, 131.82, 128.73, 120.97, 117.95, 115.95, 75.13, 37.32, 24.94.

EI-MS *m/z*: $[M]^+$ Calcd for $C_{14}H_{17}NO$ 215.30; Found: 215.1

1.3.5. General Procedure for Ring-Closing Metathesis

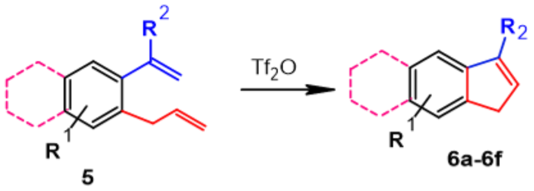

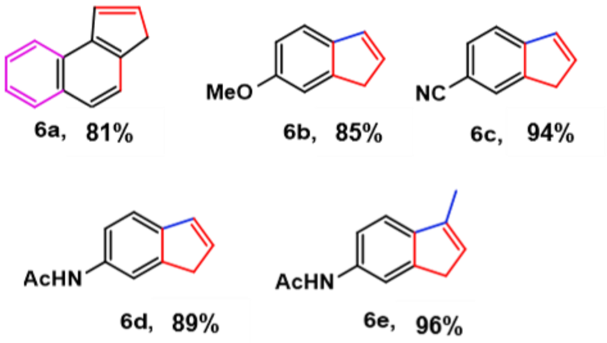

The diene (1 mmol unless otherwise noted) was dissolved in 5 mL of dry CH_2Cl_2, and nitro-Hoveyda−Grubbs catalyst (1 mol %) was added and refluxed for 1 h. Then, the reaction mixture was concentrated and purified by column chromatography.

3*H*-benz[e]indene (6a)

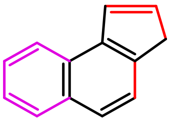

0.25 mmol scale, white solid, 81%; **^{1}H NMR (600 MHz, CDCl$_3$)** δ 7.97 (dd, *J* = 8.3, 1.0 Hz, 1H), 7.88 (d, *J* = 8.2 Hz, 1H), 7.78 (d, *J* = 8.3 Hz, 1H), 7.59 (d, *J* = 8.3 Hz, 1H), 7.49 (ddd, *J* = 8.2, 6.8, 1.3 Hz, 1H), 7.40 (ddd, *J* = 8.1, 6.8, 1.2 Hz, 1H), 7.05 – 6.98 (m, 1H), 6.67 (dt, *J* = 5.5, 1.8 Hz, 1H), 3.73 (t, *J* = 1.6 Hz, 2H).

^{13}C NMR (150 MHz, CDCl$_3$) δ 133.74, 132.73, 131.56, 129.82, 128.91, 127.18, 126.23, 124.56, 123.60, 123.30, 120.59, 118.97, 38.13.

EI-MS *m/z*: $[M]^+$ Calcd for $C_{13}H_{10}$ 166.22; Found: 166.1

6-methoxy-1H-indene (6b)

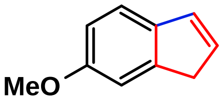

0.25 mmol scale, beige solid, 85%; **^{1}H NMR (400 MHz, CDCl$_3$)** δ 7.31 – 7.25 (m, 1H), 7.08 (dd, *J* = 1.6, 0.6 Hz, 1H), 6.87 – 6.79 (m, 2H), 6.42 (dt, *J* = 5.5, 2.0 Hz, 1H), 3.83 (s, 3H), 3.37 (bt, 2H).

^{13}C NMR (101 MHz, CDCl$_3$) δ 157.89, 145.65, 138.07, 132.04, 131.55, 121.18, 111.98, 110.32, 55.65, 39.23.

EI-MS *m/z*: $[M]^+$ Calcd for $C_{10}H_{10}O$ 146.19; Found: 146.2

1*H*-indene-6-carbonitrile (6c)

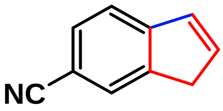

0.22 mmol scale, beige solid, 94%; **^{1}H NMR (600 MHz, CDCl$_3$)** δ 7.32 – 7.22 (m, 1H), 7.06 (d, J = 1.6 Hz, 1H), 6.86 – 6.76 (m, 2H), 6.40 (d, J = 5.6 Hz, 1H), 3.36 (s, 2H).

^{13}C NMR (150 MHz, CDCl$_3$) δ 13C NMR (101 MHz,) δ 145.65, 138.70, 136.60, 130.97, 128.67, 121.59, 121.17, 111.98, 110.32, 39.23.

EI-MS *m/z*: $[M]^+$ Calcd for $C_{10}H_7N$ 141.17; Found: 141.1

***N*-(1H-inden-6-yl)acetamide (6d)**

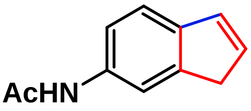

2.28 mmol scale, beige solid, 350 mg, 89%; **^{1}H NMR (400 MHz, CDCl$_3$)** δ 7.77 (s, 1H), 7.30 (d, *J* = 8.1 Hz, 1H), 7.22 (dd, *J* = 8.0, 1.5 Hz, 2H), 6.82 (d, *J* = 5.5 Hz, 1H), 6.57 – 6.40 (m, 1H), 3.38 (s, 2H), 2.17 (s, 3H).

^{13}C NMR (101 MHz, CDCl$_3$) δ 168.28, 144.85, 141.50, 134.96, 133.95, 131.58, 120.93, 118.38, 116.44, 39.30, 29.78.

EI-MS *m/z*: $[M]^+$ Calcd for $C_{11}H_{11}NO$ 173.22; Found: 173.3

***N*-(3-methyl-1H-inden-6-yl)acetamide (6e)**

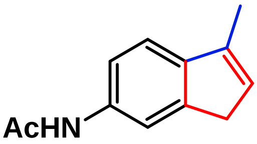

0.80 mmol scale, beige solid, 145 mg, 96%; **^{1}H NMR (400 MHz, CDCl$_3$)** δ 7.72 (s, 1H), 7.28 (dd, *J* = 8.1, 1.8 Hz, 1H), 7.23 (d, *J* = 8.1 Hz, 1H), 7.17 (s, 1H), 6.14 (d, *J* = 1.6 Hz, 1H), 3.38 – 3.22 (m, 2H), 2.19 (s, 3H), 2.13 (dd, *J* = 3.8, 2.1 Hz, 3H).
^{13}C NMR (101 MHz, CDCl$_3$) δ 168.41, 145.41, 142.85, 139.59, 134.95, 128.51, 118.88, 118.25, 116.38, 37.80, 24.68, 13.12.

EI-MS *m/z*: $[M]^+$ Calcd for $C_{12}H_{13}NO$ 187.24; Found: 187.1

1.3.6. General procedure for Diels-Alder coupling 7a-7e

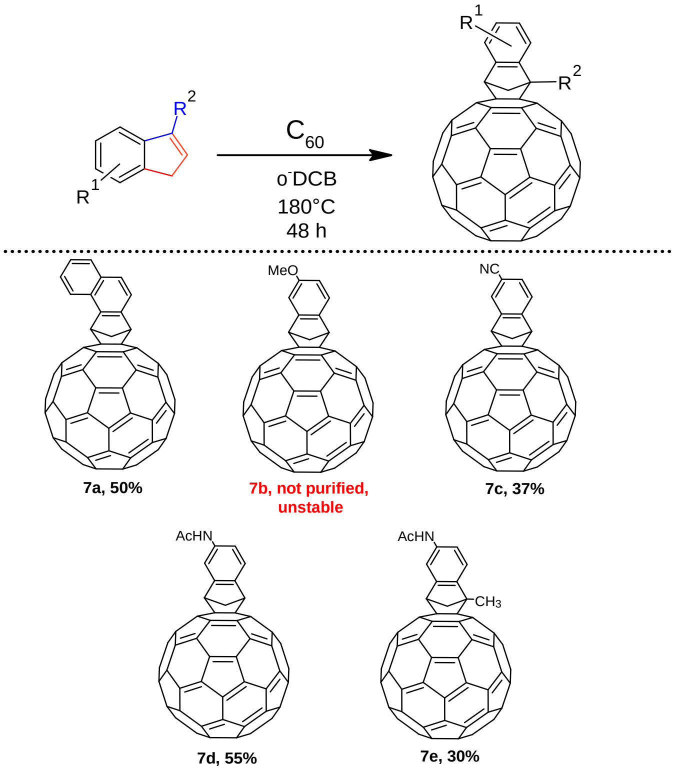

A mixture of C60 (1 equiv) and indene derivative (1 equiv) in o-dichlorobenzene (*o*DCB), concentration *ca.* 0,15 M, was heated to reflux (180 °C) under argon atmosphere for 48 h. The reaction mixture was cooled down and solvent was evaporated in vacuo. Column chromatography (silica gel; toluene, then gradient toluene:ethyl acetate from 10% to 100%) gave the crude product and unreacted fullerene (toluene fraction). The crude product was further purified on a second silica gel column to remove various side-products and a small amount of residual C60. The fractions containing the desired product were combined and concentrated in vacuo to give brown solid.

Benzo-ICMA–*6',7'-Benzeno-1',4'-dihydro-naphtho[2',3':1,2][5,6]fullerene-C_{60}* (7a)

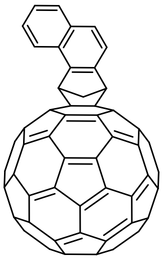

0.12 mmol scale, brown solid, 50%; **^{1}H NMR (600 MHz, $CDCl_3$)** δ 8.21 – 8.13 (m, 1H), 8.04 – 7.99 (m, 1H), 7.97 (dd, J = 8.2, 0.9 Hz, 1H), 7.84 (d, J = 8.2 Hz, 1H), 7.54 (ddd, J = 8.1, 6.9, 1.4 Hz, 2H), 5.55 (q, J = 1.7 Hz, 1H), 5.13 (q, J = 1.6 Hz, 1H), 3.98 (dt, J = 9.9, 1.5 Hz, 1H), 3.02 (dt, J = 9.9, 1.8 Hz, 1H); **^{13}C NMR (151 MHz, $CDCl_3$)** δ 156.65, 156.34, 154.86, 154.74, 147.21, 147.20, 146.36, 146.33, 146.17, 146.12, 146.10, 146.07, 146.02, 146.01, 145.90, 145.87, 145.55, 145.45, 145.42, 145.40, 145.39, 145.28, 145.27, 145.08, 145.07, 144.91, 144.65, 144.58, 144.38, 144.30, 144.08, 142.76, 142.67, 142.66, 142.55, 142.42, 142.36, 142.34, 142.29, 142.27, 142.09, 142.05, 142.04, 141.87, 141.84, 141.80, 141.78, 140.16, 140.13, 139.76, 139.69, 137.90, 137.50, 137.48, 136.97, 133.14, 130.56, 129.47, 129.18, 129.06, 128.25, 127.87, 127.73, 126.89, 125.46, 125.32, 123.88, 122.47, 59.13, 55.95, 31.95, 29.68, 29.39; **HRMS (ESI) *m/z*:** $[M+H]^+$ Calcd for $C_{73}H_{11}$ 887.0861; Found: 887.0856

MeO-ICMA–*6'-Methoxy-1',4'-dihydro-naphtho[2',3':1,2][5,6]fullerene-C$_{60}$* (7b)

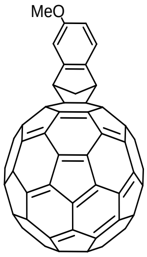

0.12 mmol scale, brown solid, **7b, not purified, unstable under purification.**

CN-ICMA –*6'-Carbonitrile-1',4'-dihydro-naphtho[2',3':1,2][5,6]fullerene-C$_{60}$* (7c)

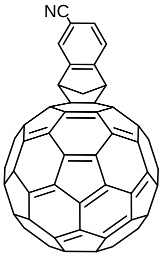

0.12 mmol scale, brown solid, 37%**^{1}H NMR (600 MHz, CDCl$_3$)** δ 7.42 – 7.35 (m, 2H), 7.11-7.02 (m, 1H), 5.59 (q, J = 1.9 Hz, 1H), 5.11 (q, J = 1.6 Hz, 1H), 3.98 (dt, J = 9.9, 1.5 Hz, 1H), 3.01 (dt, J = 10.0, 1.7 Hz, 1H); **^{13}C NMR (151 MHz, CDCl$_3$)** δ 155.39, 155.35, 154.02, 153.95, 151.35, 147.45, 147.25, 146.50, 146.39, 146.29, 146.21, 146,14, 146,12, 146,11, 145,91, 145.85, 145.63, 145.58, 145.52, 145.43, 145.36, 145.30, 145.20, 144.72, 144.54, 144.46, 143.20, 143.03, 142.82, 142.73, 142,70, 142,52, 142.35, 142,31, 142.25, 142.18, 142.10, 142.05, 141.95, 141.89, 140.34, 139.98, 139.91, 138.72, 137.98, 137.58, 137.42, 136.62, 132.18, 131.82, 130.99, 130.64, 129.14, 128.69, 128.33, 127.82, 127.51, 127.17, 125.40, 125.02, 124.44, 121.61, 119.40, 111.19, 74.79, 58.01, 57.55, 46.12, 29.80, 21.56; **HRMS (ESI) *m/z*:** [M+H]$^+$ Calcd for $C_{70}H_8N$ 862.0657; Found: 862.0648

NHAc-ICMA –*6'-Acetamido-1',4'-dihydro-naphtho[2',3':1,2][5,6]fullerene-C$_{60}$* (7d)

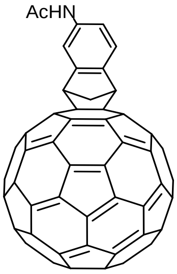

0.87 mmol scale, brown solid, 427 mg, 55%; **^{1}H NMR (600 MHz, CDCl$_3$)** δ 7.97 (d, J = 2.0 Hz, 1H), 7.60 (d, J = 8.0 Hz, 1H), 7.48 (dd, J = 8.0, 2.0 Hz, 1H), 7.36 (s, 1H), 4.95 (dd, J = 17.2, 1.7 Hz, 2H), 3.88 (dt, J = 10.2, 1.5 Hz, 1H), 2.94 (dt, J = 10.3, 1.7 Hz, 1H), 2.27 (s, 3H); **^{13}C NMR (151 MHz, CDCl$_3$)** δ 168.39, 156.97, 155.28, 154.51, 147.23, 147.21, 146.98, 146.89, 146.83, 146.56, 146.35, 146.18, 146.09, 146.03, 145.94, 145.63, 145.46, 145.42, 145.24, 145.19, 145.11, 145.09, 145.06, 145.04, 145.00, 144.96, 144.72, 144.61, 144.53, 144.39, 144.21, 144.09, 143.34, 142.86, 142.70, 142.66, 142.51, 142.39, 142.34, 142.33, 142.22, 142.14, 142.06, 141.95, 141.83, 141.76, 140.88, 140.16, 139.76, 139.56, 139.48, 137.55, 137.32, 137.10, 137.04, 132.49, 129.78, 129.03, 128.66, 128.47, 127.72, 124.38, 122.56, 118.51, 115.82, 65.42, 60.55, 57.19, 52.24, 29.73, 24.80,; **HRMS (ESI) *m/z*:** [M + Na]$^+$ Calcd for $C_{71}H_{11}NONa$ 916.0738; Found: 916.0728.

NHAc-Me-ICMA *–6'-Acetamido-1',4'-dihydro-3'-methyl-naphtho[2',3':1,2][5,6]fullerene-C_{60}* **(7e)**

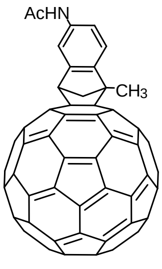

0.53 mmol scale, brown solid, 143 mg, 30%; **^{1}H NMR (600 MHz, $CDCl_3$)** δ 7.91 (m, 1H), 7.42 – 7.28 (m, 3H), 4.71 (m, 1H), 3.73 (d, *J* = 9.8 Hz, 1H), 2.78 (d, *J* = 10.2 Hz, 1H), 2.32 (s, 3H), 2.23 (s, 3H); **^{13}C NMR (151 MHz, $CDCl_3$)** δ 168.31, 157.14, 155.36, 154.31, 147.21, 147.18 , 146.98, 146.89, 146.83, 146.50, 146.32, 146.15, 146.09, 145.99, 145.92, 145.69, 145.46, 145.41, 145.32, 145.24, 145.11, 145.08, 145.05, 145.04, 144,97, 144,90, 144.70, 144.61, 144.58, 144.39, 144.25, 144.09, 143.12, 142.86, 142.69, 142.65, 142.54, 142.42, 142.36, 142.31, 142.22, 142.14, 142.06, 141.95, 141.83, 141.76, 140.88, 140.16, 139.76, 139.56, 139.48, 137.55, 137.32, 137.10, 136.98, 134.49, 129.78, 129.03, 128.60, 128.40, 127.70, 127.02, 122.56, 118.51, 115.85, 65.45, 60.57, 57.05, 52.00, 29.72, 24.82, 16.84; **HRMS (ESI) *m/z*: [M + Na]$^+$** Calcd for $C_{72}H_{13}NONa$ 930.0895; Found: 930.0868.

1.3.7. Fullerene adduct with free amine (8)

NH2-ICMA *–6'-Amino-1',4'-dihydro-3'-methyl-naphtho[2',3':1,2][5,6]fullerene-C_{60}* **(8)**

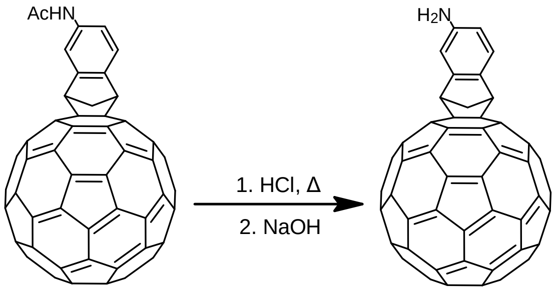

7d **8** 75%

NHAc-ICMA (50 mg, 0.06 mmol) was suspended in methanol and 1 mL of 12M hydrochloric acid was added. Reaction mixture was reacted in 60 °C for 2 hours. After that time reaction mixture was cooled down to room temperature and basified with 2M NaOH, then triple extracted with ethyl acetate (3 x 10 mL). After drying with the $NaSO_4$ residue was evaporated to dryness to afford 36 mg of brown solid (75%).

^{1}H NMR (600 MHz, $CDCl_3$) δ 7.94 (s, 1H), 7.63 – 7.12 (m, 4H), 4.86 – 4.83 (m, 2H), 3.83 – 3.78 (m, 1H), 2.88 – 2.82 (m, 1H); **^{13}C NMR (151 MHz, $CDCl_3$)** δ 156.21, 154.32, 154.19, 147.27, 147.17, 146.98, 146.93, 146.85, 146.84, 146.50, 146.33, 146.27, 146.14, 146.13, 146.11, 146.06, 146,01, 145.70, 145.54, 145.43, 145.28, 145.19, 145.11, 145.07, 145.05, 145.02, 145.00, 144.94, 144.72, 144.61, 144.63, 144.43, 144.33, 143.13, 142.88, 142.69, 142.65, 142.55, 142.42, 142.34, 142.31, 142.20, 142.11, 142.02, 141.95, 141.89, 141.79, 141.06, 140.19, 139.75, 137.57, 137.39, 137.30, 137.16, 136.98, 134.51, 133.15, 129.84, 129.05, 128.24, 125.31, 124.44, 118.53, 116.05, 58.49, 58.30, 57.68, 46.18, 29.71; **HRMS (ESI) *m/z*:** [M]$^+$ Calcd for $C_{69}H_9N$ 852.0813; Found: 852.0794.

1.4. ADDITIONAL DATA

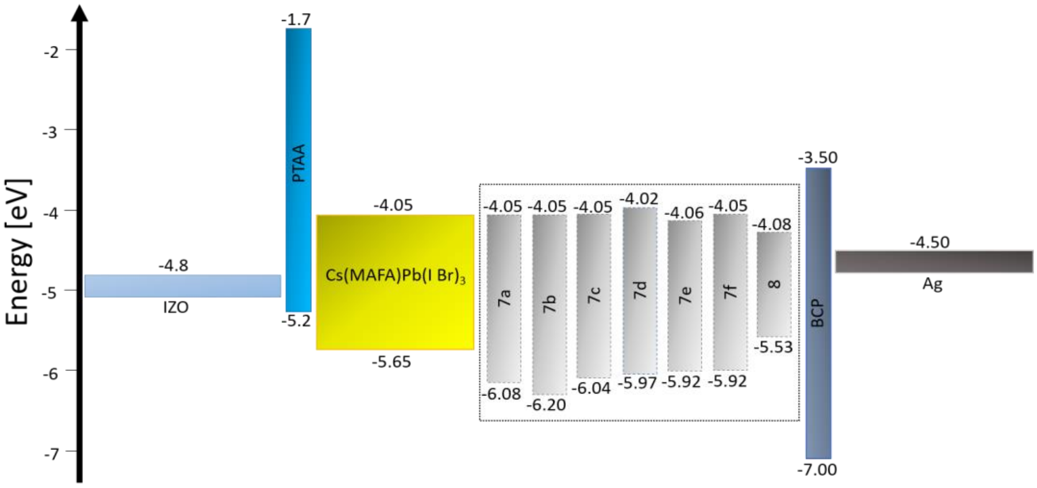

Figure S4. *Schematic illustration of the estimated HOMO and LUMO energy levels, determined from electrochemically derived first ionization potential and first electron affinity.*

1.4.1. CV Measurements

The highest occupied molecular orbital (HOMO) and the lowest unoccupied molecular orbital (LUMO) levels of fullerene derivatives are of great importance considering their use as electron acceptors and hole blocking materials in PSCs. We derived experimental values of these energy levels by cyclic voltammetry (CV) measurements and correlated them with high quality Density Functional Theory (DFT) calculations (see Supporting Information for the detailed methodology). The functionalization of the fullerene core with the indene moiety shifts the onset of the first reduction potential to more negative values when compared to the pristine C60, indicating an electron-releasing effect of the indene moiety (**Figure S5-S11**). Despite the functional groups vary significantly in terms of their electronic effects (electron-releasing / electron-withdrawing traits), when decorating the indene unit they induce negligible variation to the electrochemical parameters, as we are depicting in Table 1. This can be rationalized with the fact that indene moiety's phenylene ring is out of the π-conjugation, with the fullerene core on account of the two sp3 carbon (>CH-, or >C<) bridge linkers, which limits the electron communication between these two sub-units mainly to the inductive interactions. The CV measurements of all the considered fullerene derivatives showed successive reversible reduction steps, typical of a fullerene electrochemistry (Figure 2a). These observations are in line with the theoretical predictions of the delocalization of the LUMOs in the studied set of molecules. The cathodic CV trace of PCBM features four reversible redox steps, corresponding to successive, one electron charging of the fullerene's π-conjugated structure. Comparing CV traces of investigated compounds with that of PCBM, it is possible to discriminate redox peaks of the fullerene core from other additional signatures, characteristic for each ICMA. Those additional CV peaks could represent an independent reduction step of the indene moiety, as well as products of its subsequent chemical follow-up reactions. Smaller currents of these additional peaks suggest either lower concentration, or lower diffusion coefficients of the electrochemically active products. This may indicate dimerization of radical anion species localized at the indene moiety of fullerene derivatives. In the anodic branch, all the investigated fullerenes oxidize irreversibly, displaying onset potentials ranging from 0.43 V (for the NH_2-ICMA) up to 1.10 V (for the MeO-ICMA) (**Figure S9 and S8**). This variation in the oxidation propensity, however, does not correlate with the electron withdrawing or releasing character of the indene moiety's functional groups. Except for the MeO-ICMA, all other investigated compounds oxidize at lower potentials than the

PCBM, once again indicating stronger electron communication of the indene pendant with the fullerene host, than that of the phenyl ring in the PCBM. Interestingly, this communication facilitates electron abstraction (oxidation) from the ICMA derivatives when compared to the PCBM, which may be puzzling, since analogous effect was observed for the process of electron introduction (reduction). This might point to a through-space interaction of the π-electrons of the fullerene and π-electrons of the indene units, sterically locked in a position, and inclined at a smaller angle above the fullerene core than the rotationally unrestricted phenyl pendant of the PCBM (**Figure S12**).

PCBM

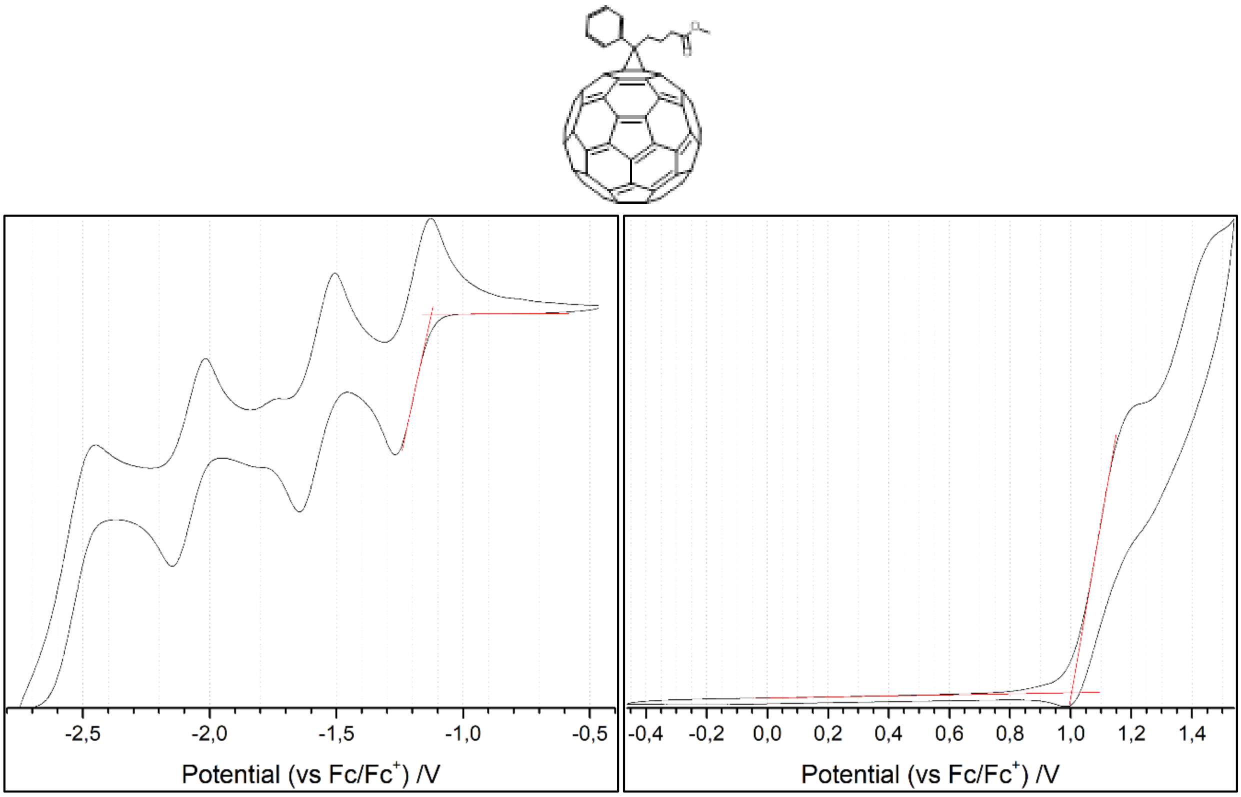

Figure S5. *Cyclic voltammograms of* ***a****) reduction, and* ***b****) oxidation processes of fullerene PCBM.*

Benzo-ICMA

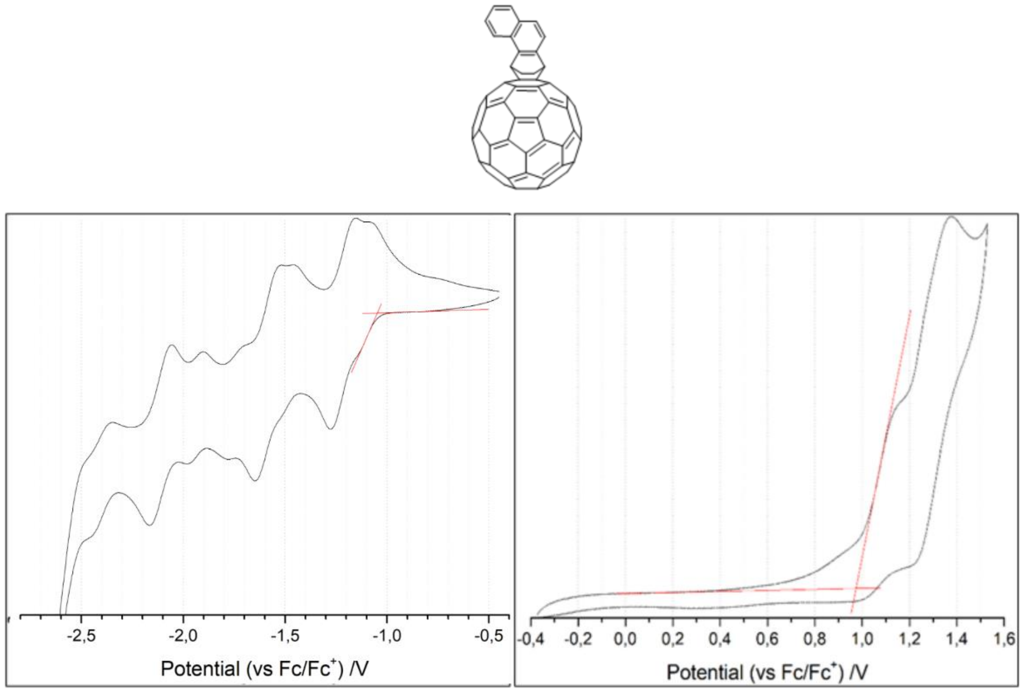

Figure S6. *Cyclic voltammograms of **a**) reduction, and **b**) oxidation processes of fullerene Benzo-ICMA*

CN-ICMA

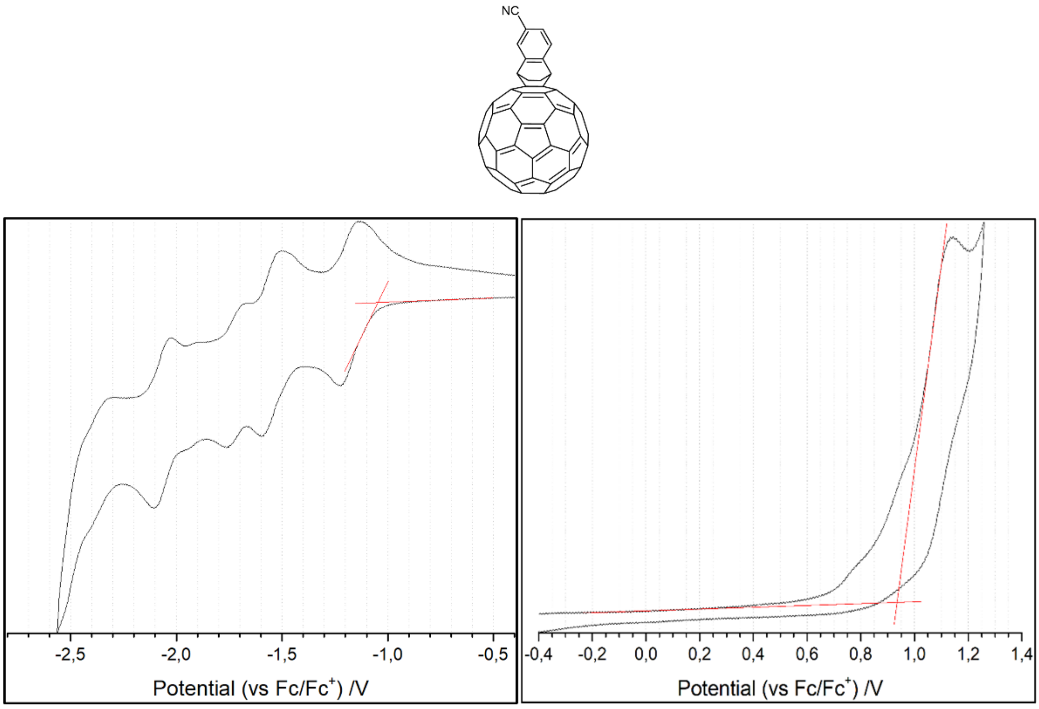

Figure S7. *Cyclic voltammograms of **a**) reduction, and **b**) oxidation processes of fullerene CN-ICMA*

MeO-ICMA

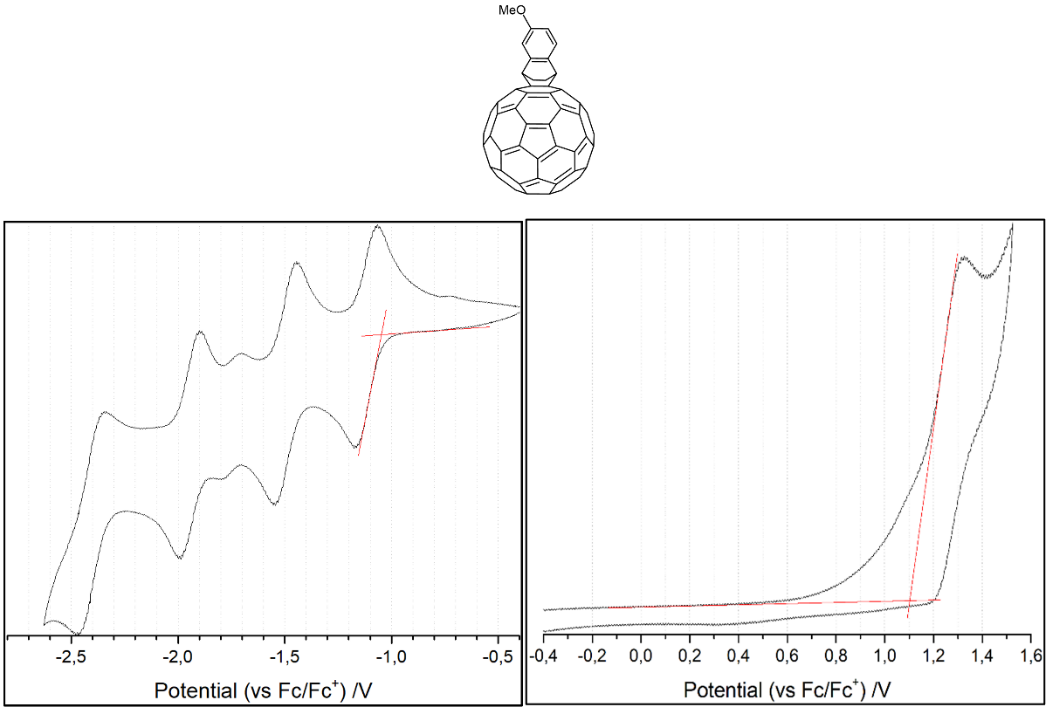

Figure S8. *Cyclic voltammograms of **a**) reduction, and **b**) oxidation processes of fullerene MeO-ICMA*

NH2-ICMA

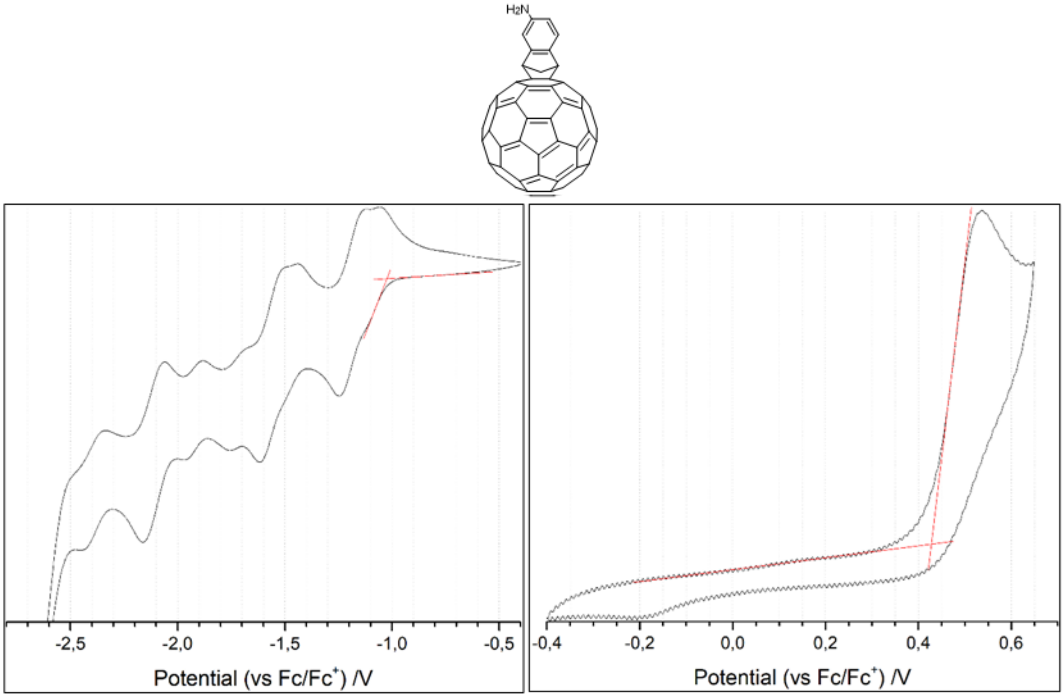

Figure S9. *Cyclic voltammograms of **a**) reduction, and **b**) oxidation processes of fullerene NH_2-ICMA*

NHAc-ICMA

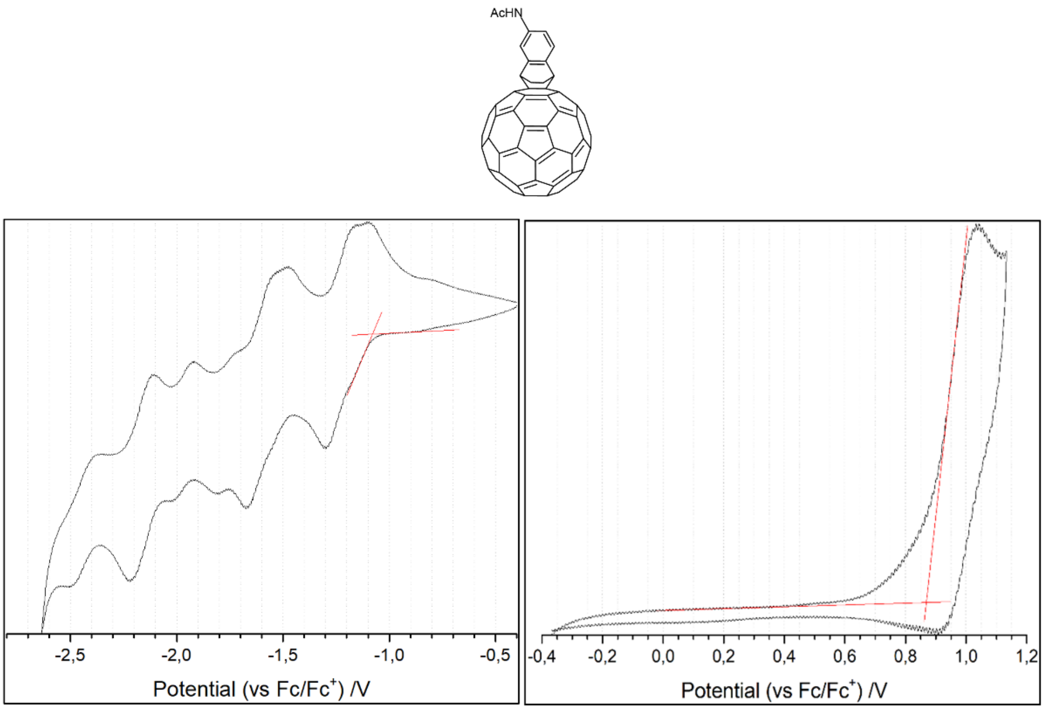

Figure S10. *Cyclic voltammograms of **a**) reduction, and **b**) oxidation processes of fullerene NHAc-ICMA*

NHAc-Me-ICMA

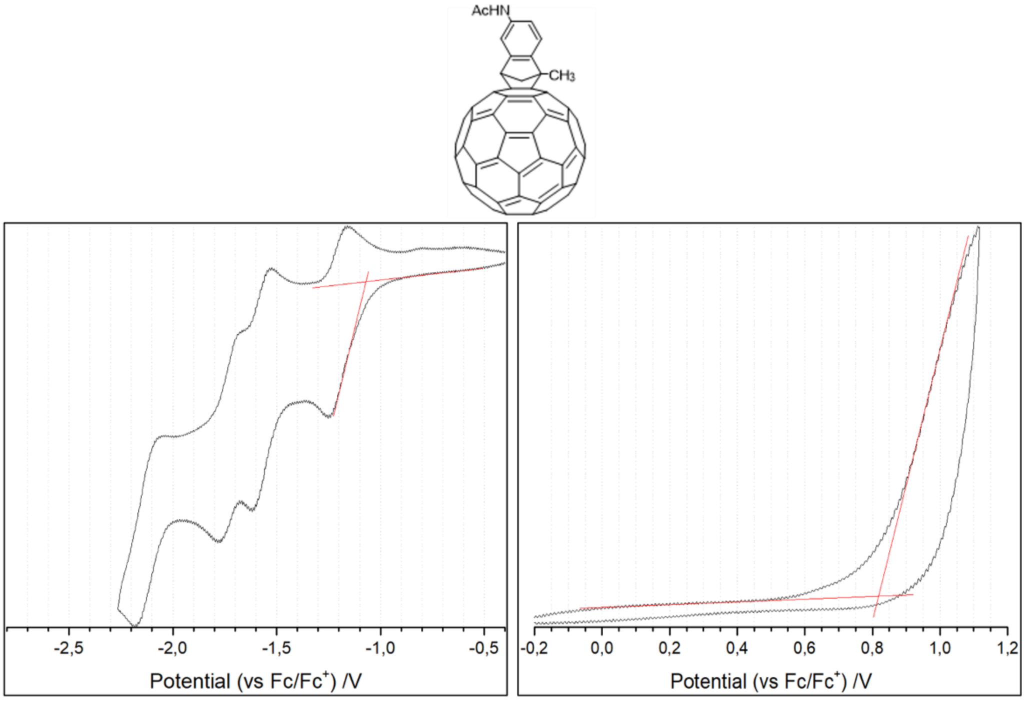

Figure S11. *Cyclic voltammograms of **a**) reduction, and **b**) oxidation processes of fullerene NHAc-Me-ICMA*

One also finds that the variation in the oxidation propensity of the ICMAs does not correlate with the electron withdrawing or releasing character of their indene moiety functional groups. When arranged in the order of increasing electron withdrawing effect of the modifying group, we should have NH_2-

ICMA with the amino group, followed by MeO-ICMA with the methoxy group, NHAc-ICMA, NH-Me-ICMA and NHAc-PE-ICMA with the acetamide group, then Benzo-ICMA with the additional benzene ring, and finally CN-ICMA with the cyanide group. On the other hand, the order of the increasing oxidation potentials doesn't follow the order of increasing electron withdrawing character. The high onset of the oxidation potential observed for the MeO-ICMA is quite surprising, as this molecule is equipped with the methoxy group, which should display strong electron-releasing functionality. Its CV trace features diffuse current signature preceding the oxidation peak (**Figure S5**), pointing to the possibility of some form of adsorption process taking place at the electrode surface, which contributes to oxidation overpotential of this molecule (**Figure S5**). The comparable oxidation onset potentials recorded for the NHAc-ICMA, NHAc-Me-ICMA and NHAc-PE-ICMA, indicate a marginal inductive electronic effect of the functionalization of the ternary (>CH-) or quaternary (>C<) carbon bridge atoms, which interfaces the fullerene and phenylene units. Considering the ferrocene/ferrocenium (Fc/Fc^+) redox couple (5.10 eV below the vacuum level) as the internal standard, we determined the electron affinity (EA) and first ionization potential (IP). All the values, including PCBM molecule, are listed in Table 1.

a) b)

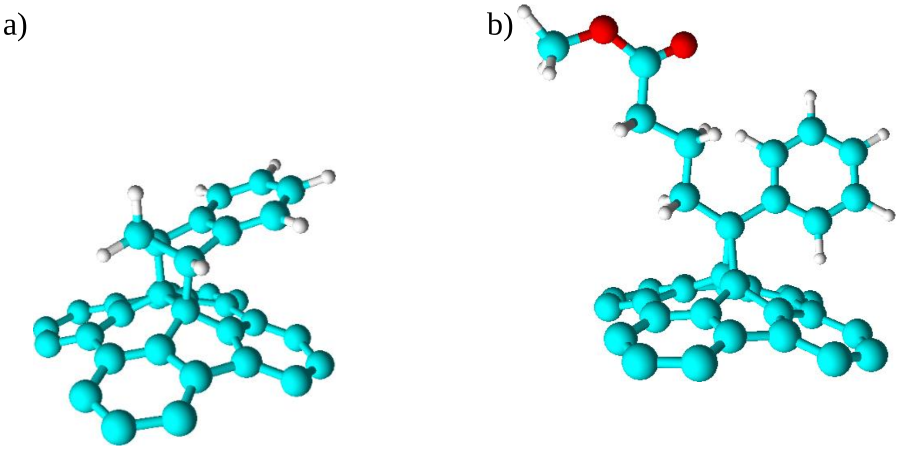

Figure S12. *Spatial arrangement of pendants in fullerene derivatives –* ***a****) sterically locked indene in ICMA, and* ***b****) rotationally unrestricted phenyl in PCBM.*

1.4.2. DFT Calculations

To gain insight in the electronic properties of the fullerene derivates, we also performed DFT calculation to evaluate the EA, IP, the electrochemical bandgap and the shape of the HOMO and LUMO orbitals. Concerning the electrochemical properties, there are two ways to estimate the oxidation/reduction potentials: (i) the vertical approximation due to the Koopmans theorem to the DFT, i.e., by taking the negative of the HOMO and LUMO single particle eigenvalues; and (ii) by moving forward to the simple single orbital energies and calculating the real oxidation and reduction Gibbs free energy differences, which are the quantities effectively corresponding to the measured adiabatic oxidation and reduction potentials, involving geometrical relaxation of the oxidized and reduced species. Both approaches have their merits, with Koopmans theorem offering a simple but approximate computational procedure, requiring only a calculation on the neutral species. The calculation of Gibbs free energies, on the other hand, is accurate but computationally more intensive. As a first approach, for the Koopmans theorem, we can approximate the EA and IP to the calculated HOMO and LUMO values. As we can see in Table 1, for the calculated HOMO values we have a nice agreement with respect to the measured IP. On the other hand, an overestimation of the EA is found when we compared to the LUMO values, and this consequentially leads to and overestimation of the calculated H-L bandgap. This is expected because

the Koopmans approximation does not take into account the reduction/oxidation energies, and related structural relaxations. To refine the theoretical results, we performed a step forward from the Koopmans approximation by evaluating the oxidation (reduction) energy in solution as differences from the optimized neutral fullerene derivates and the optimized cation (anion). As we can see by simulating the EA and IP we are increasing the agreement with the experimental measurements, even if the theoretical electrochemical bandgaps are still slightly overestimated.

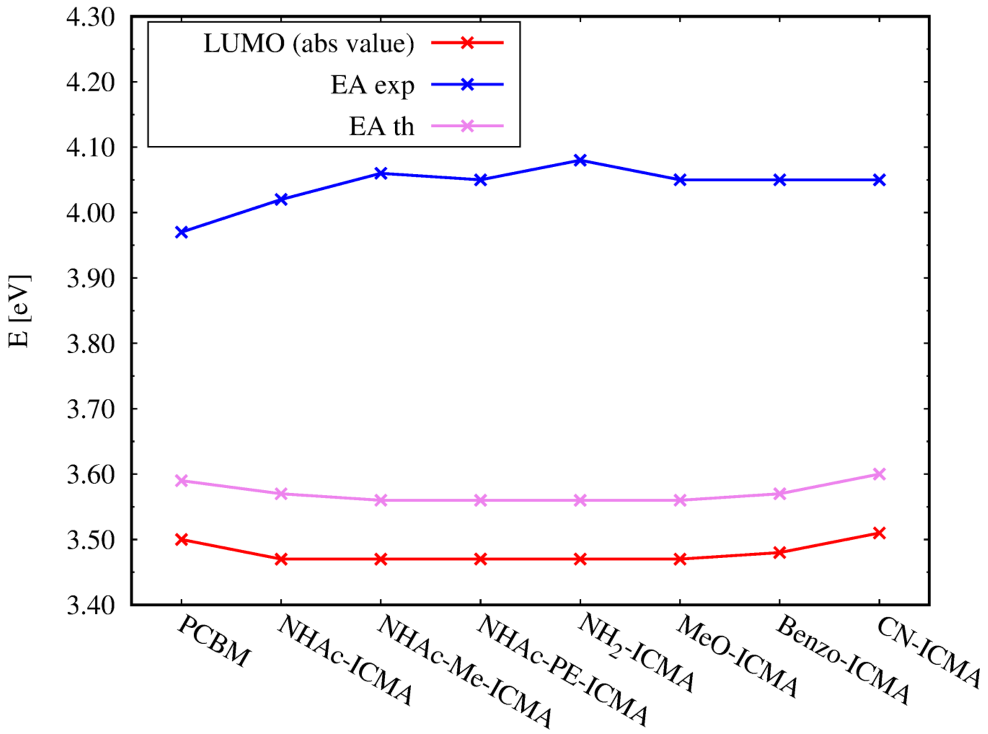

Figure S13. *Comparison between experimental and theoretical electron affinity (EA) and theoretical absolute values of LUMO energies (in eV).*

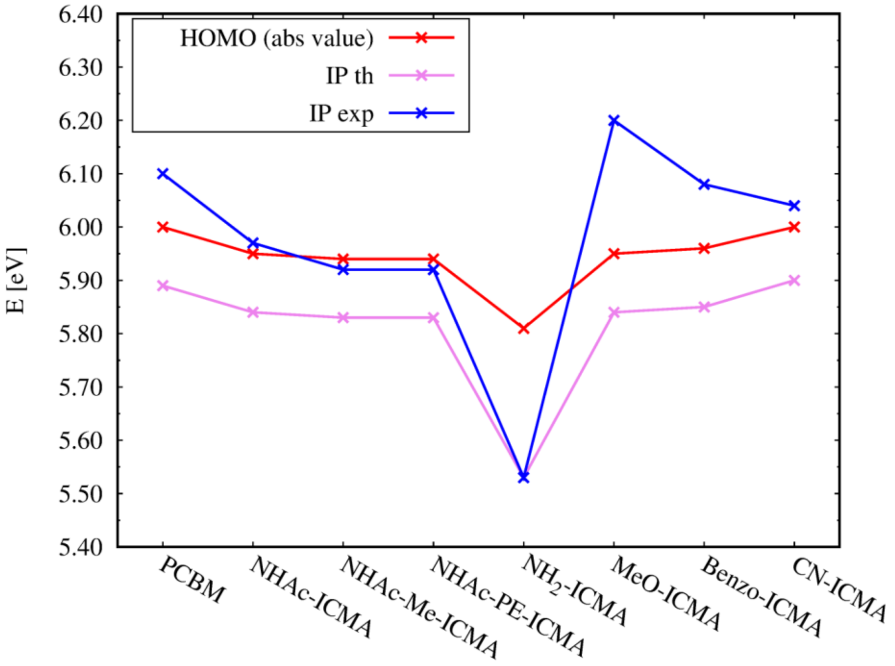

Figure S14. *Comparison between experimental and theoretical first ionization potential (IP) and theoretical absolute values of HOMO energies (in eV).*

1.4.3. NMR and MS spectra

NMR (1a-5e) and EI-MS Spectra (4a-5e) for precursors

2a

^{1}H NMR

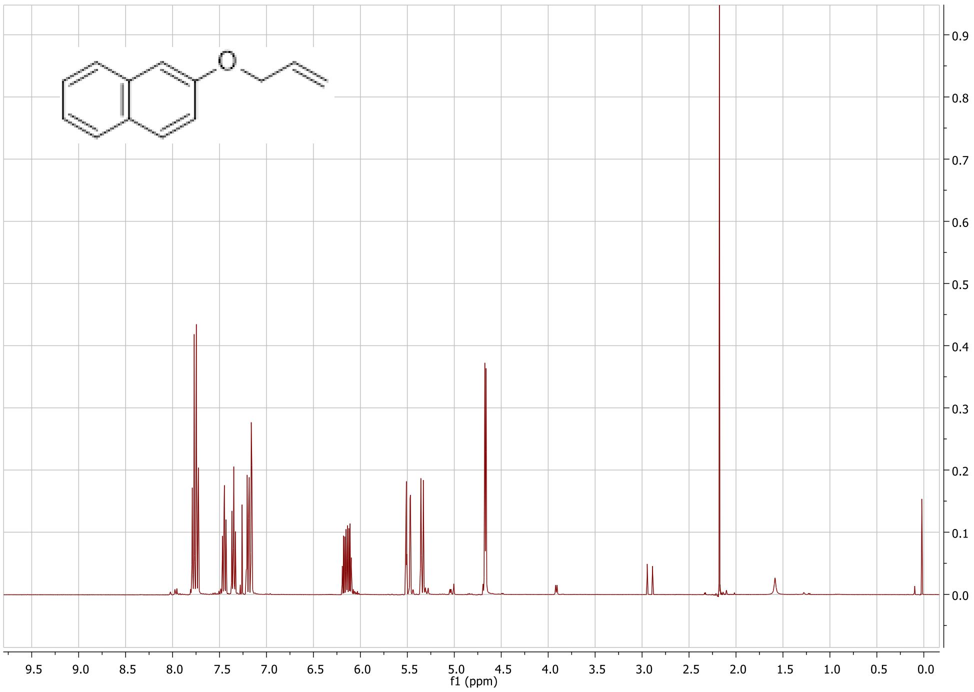

^{13}C NMR

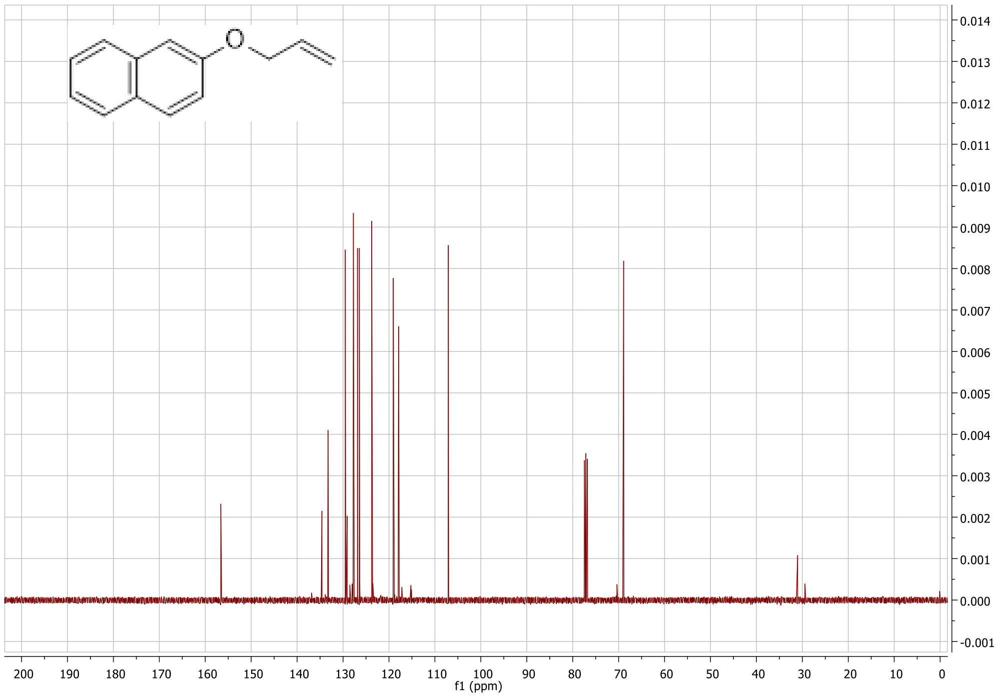

2b

^{1}H NMR

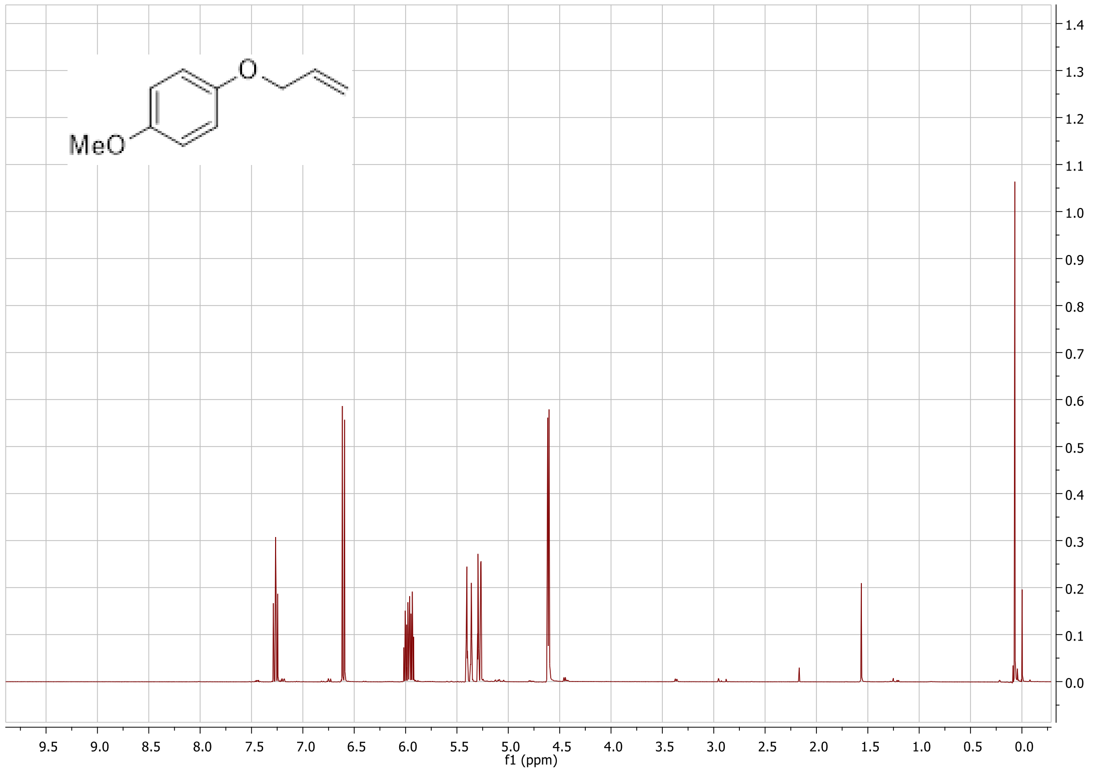

^{13}C NMR

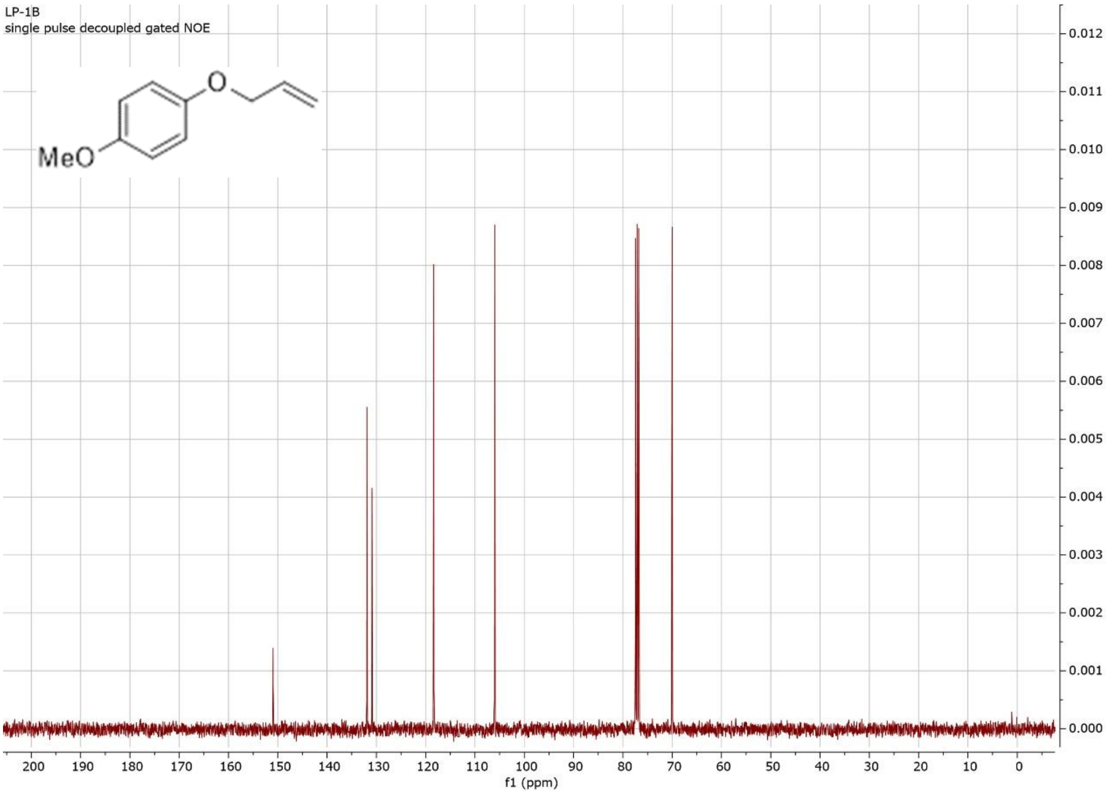

LP-1B
single pulse decoupled gated NOE
MeO
O
0.012
0.011
0.010
0.009
0.008
0.007
0.006
0.005
0.004
0.003
0.002
0.001
0.000
200 190 180 170 160 150 140 130 120 110 100 90 80 70 60 50 40 30 20 10 0
f1 (ppm)

2c

^{1}H NMR

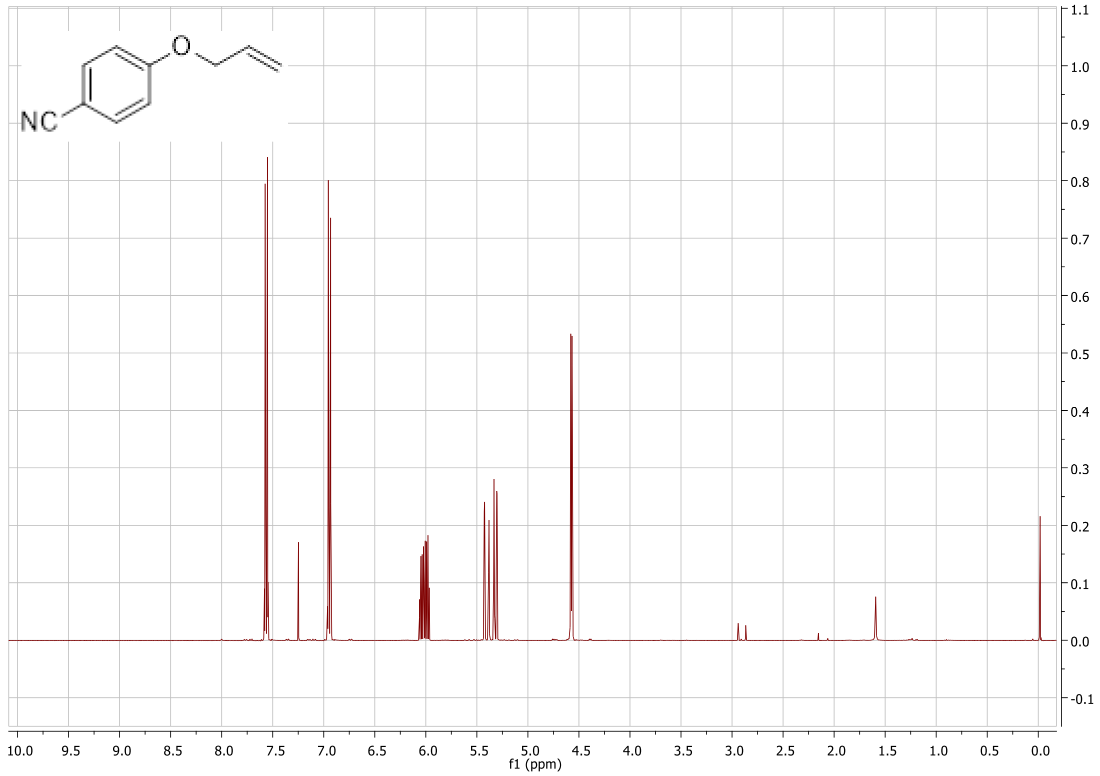

^{13}C NMR

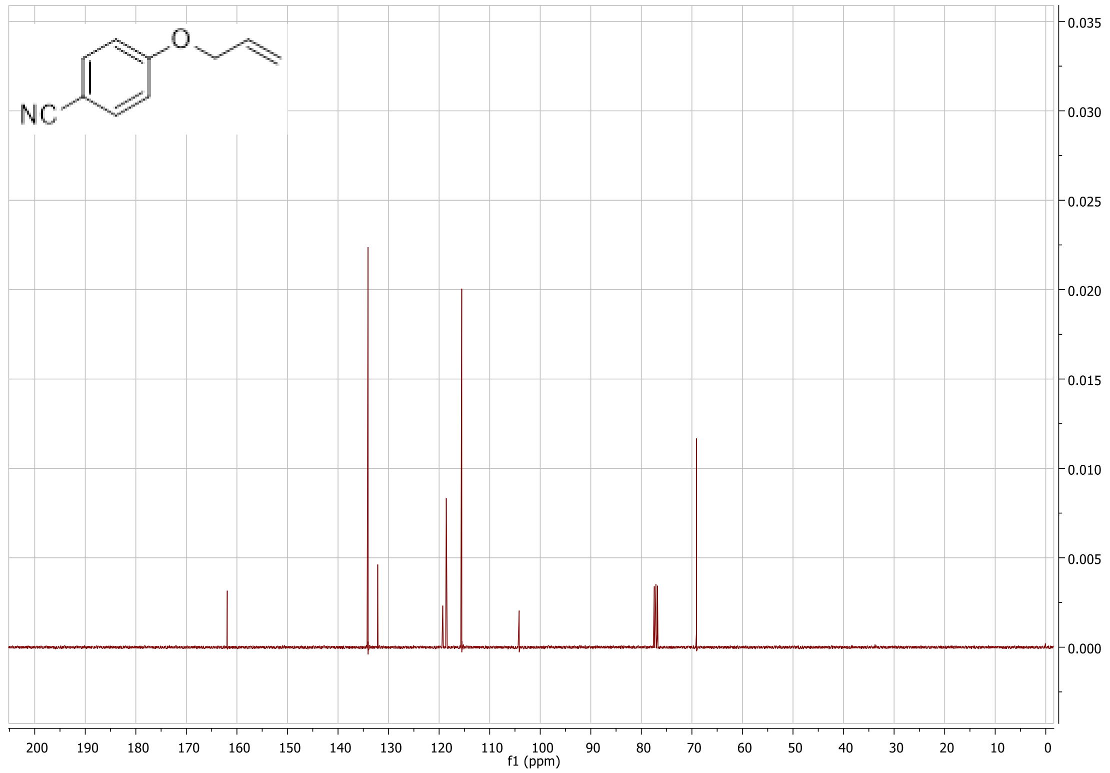

2d

^{1}H NMR

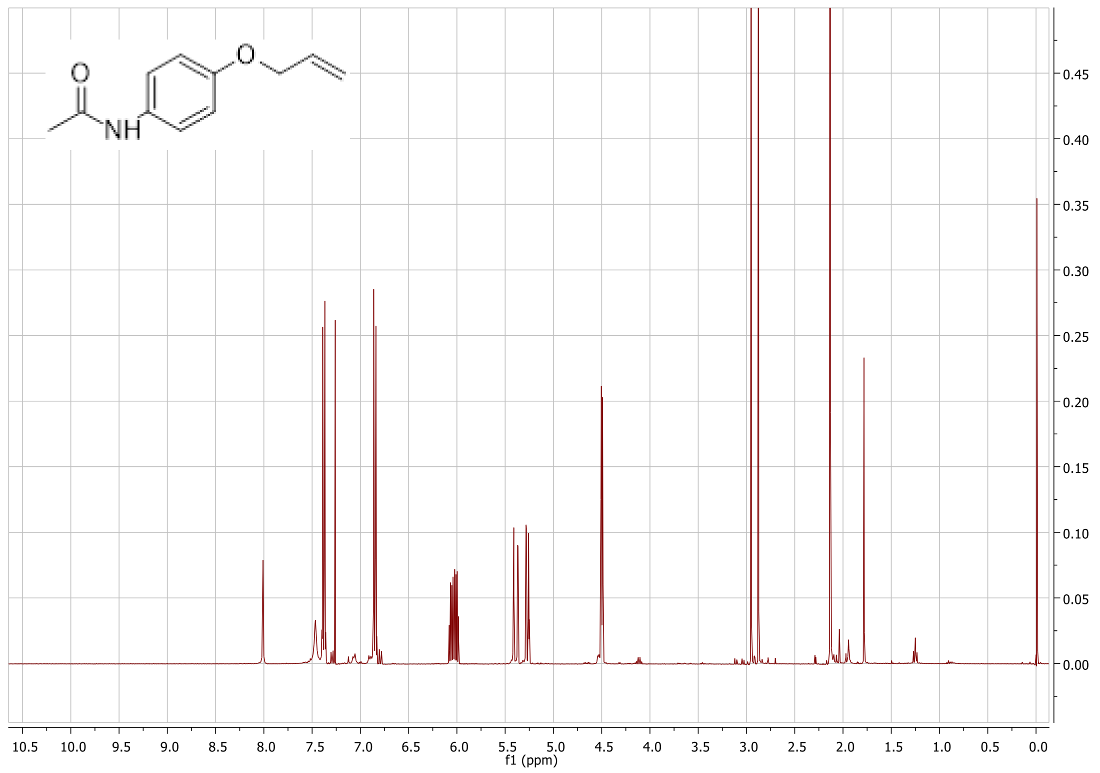

^{13}C NMR

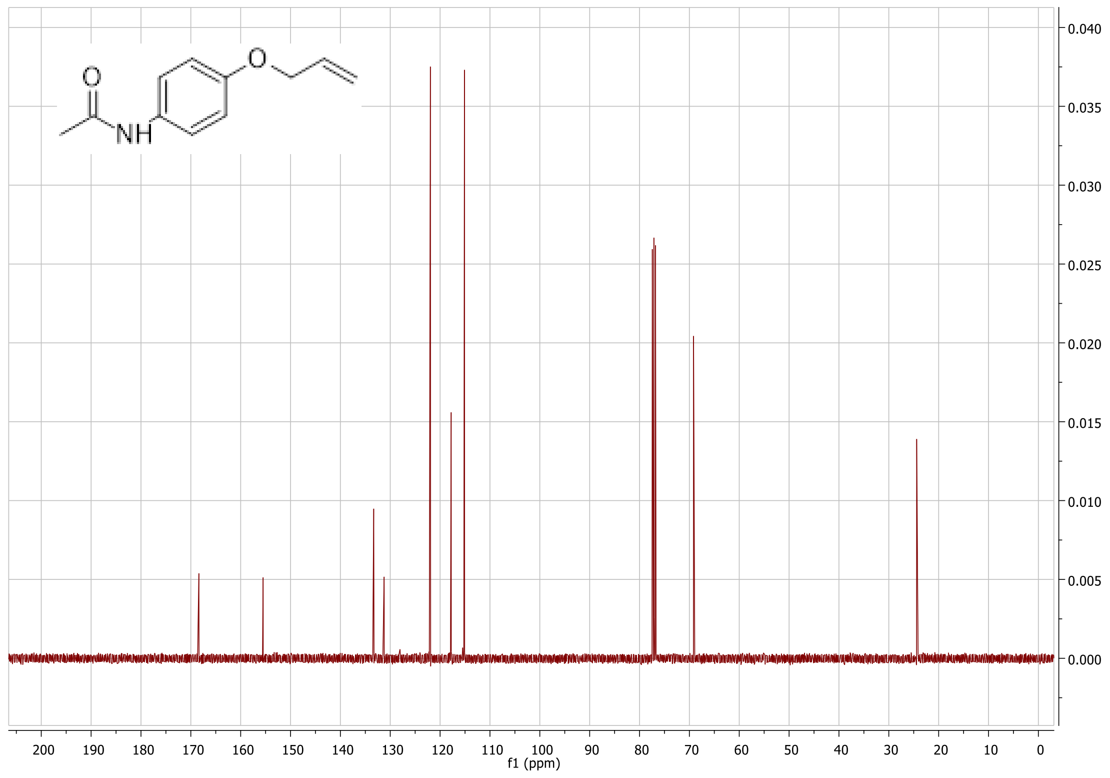

3a

^{1}H NMR

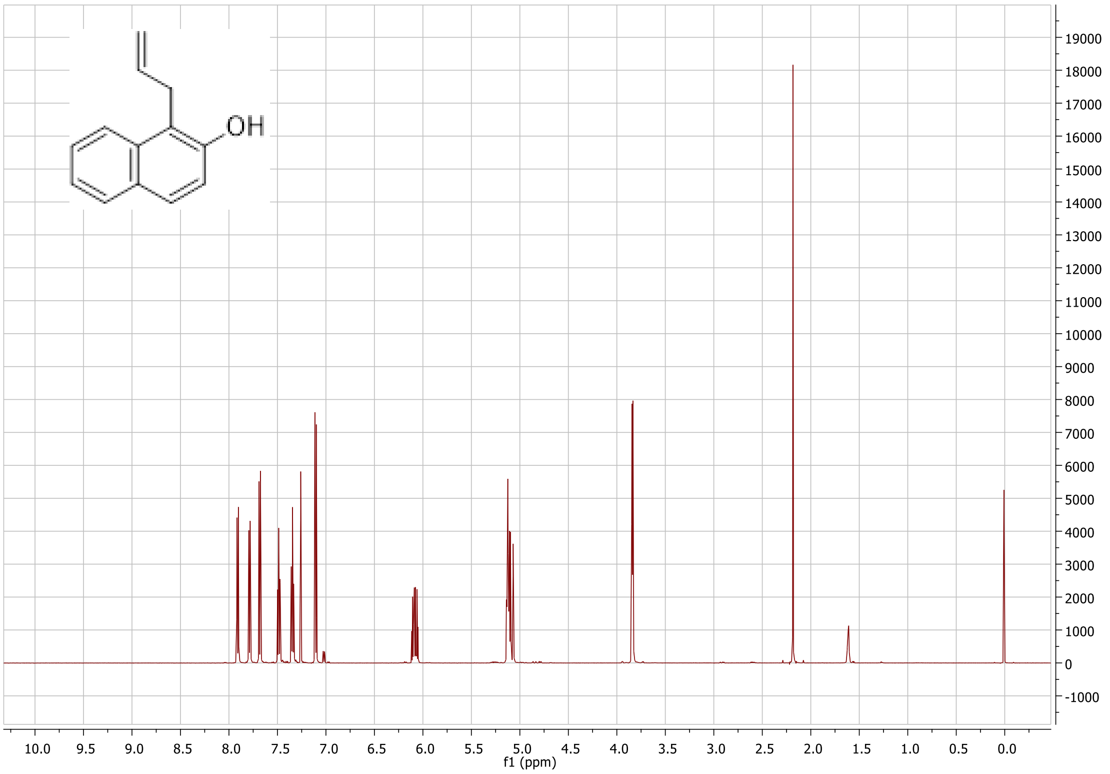

^{13}C NMR

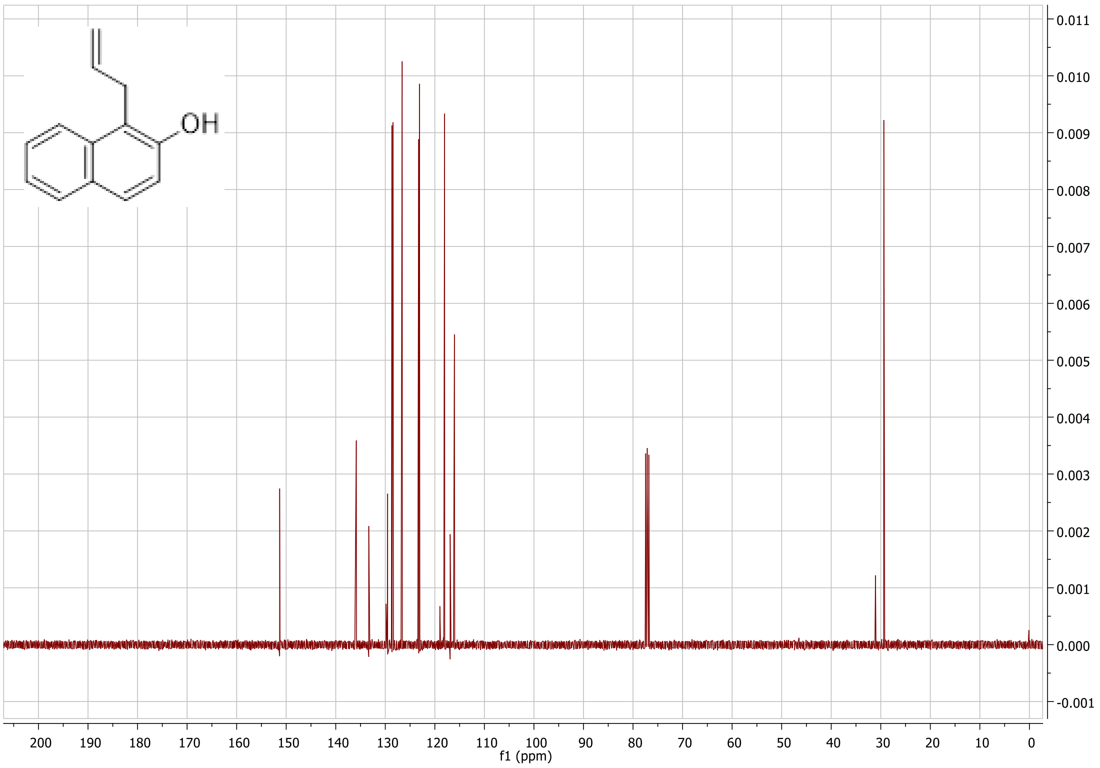

3b

^{1}H NMR

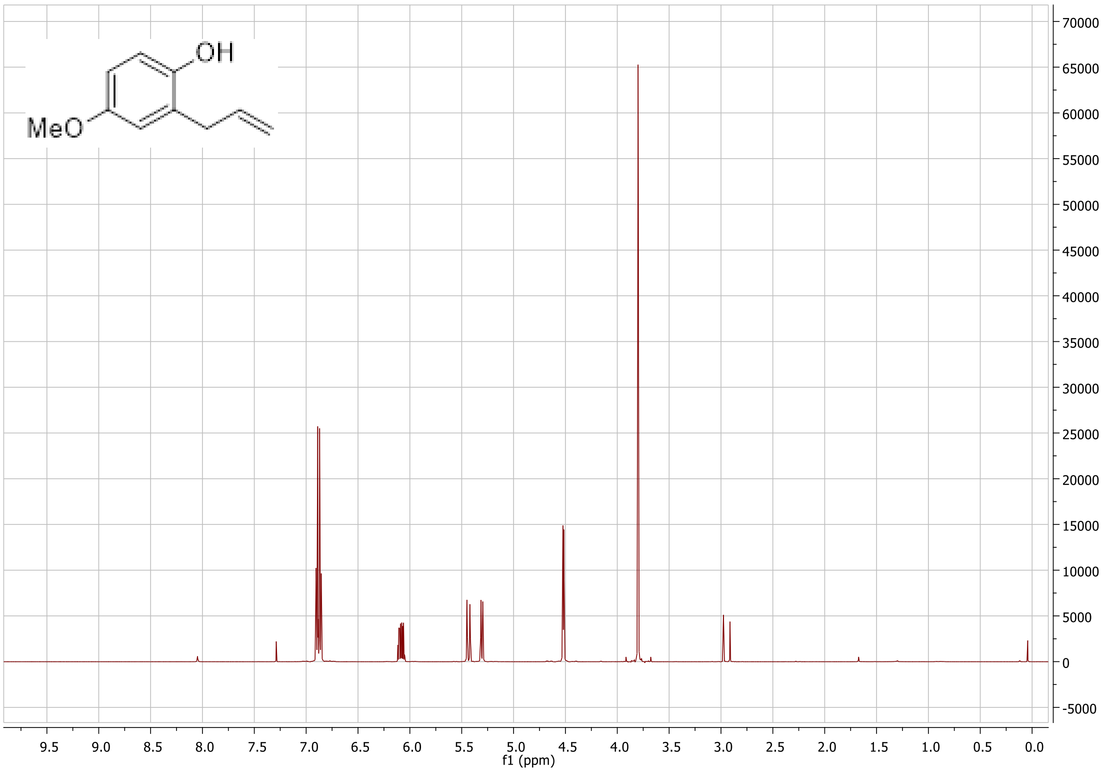

^{13}C NMR

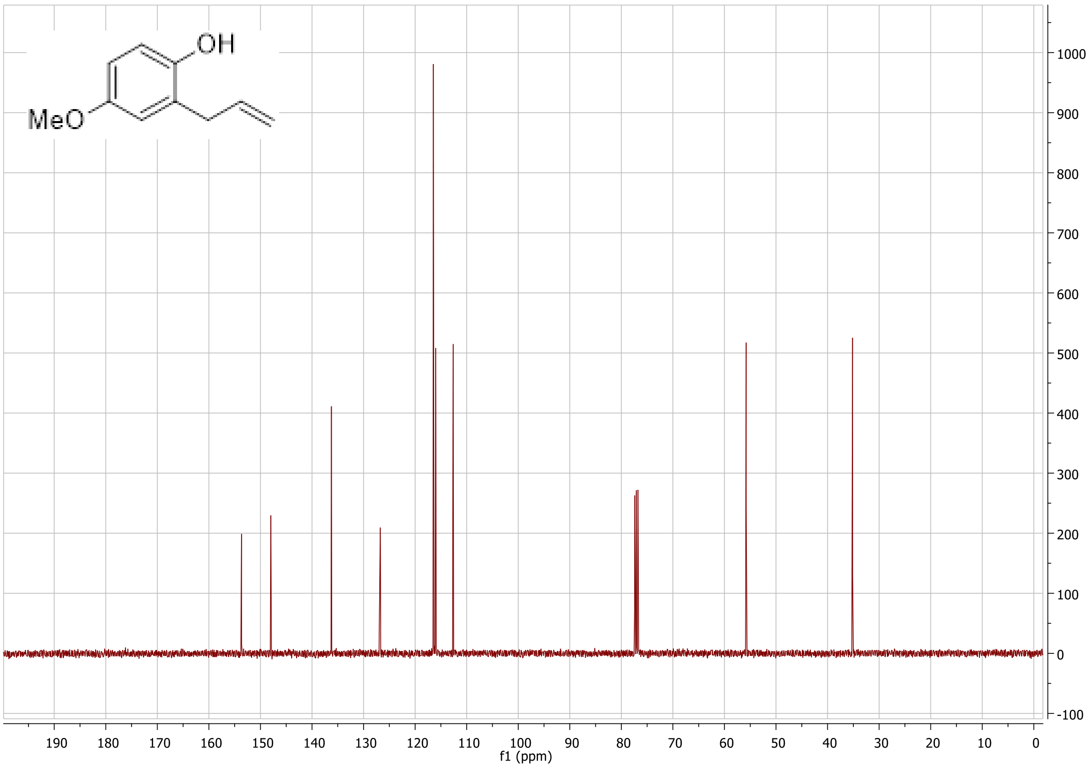

3c

^{1}H NMR

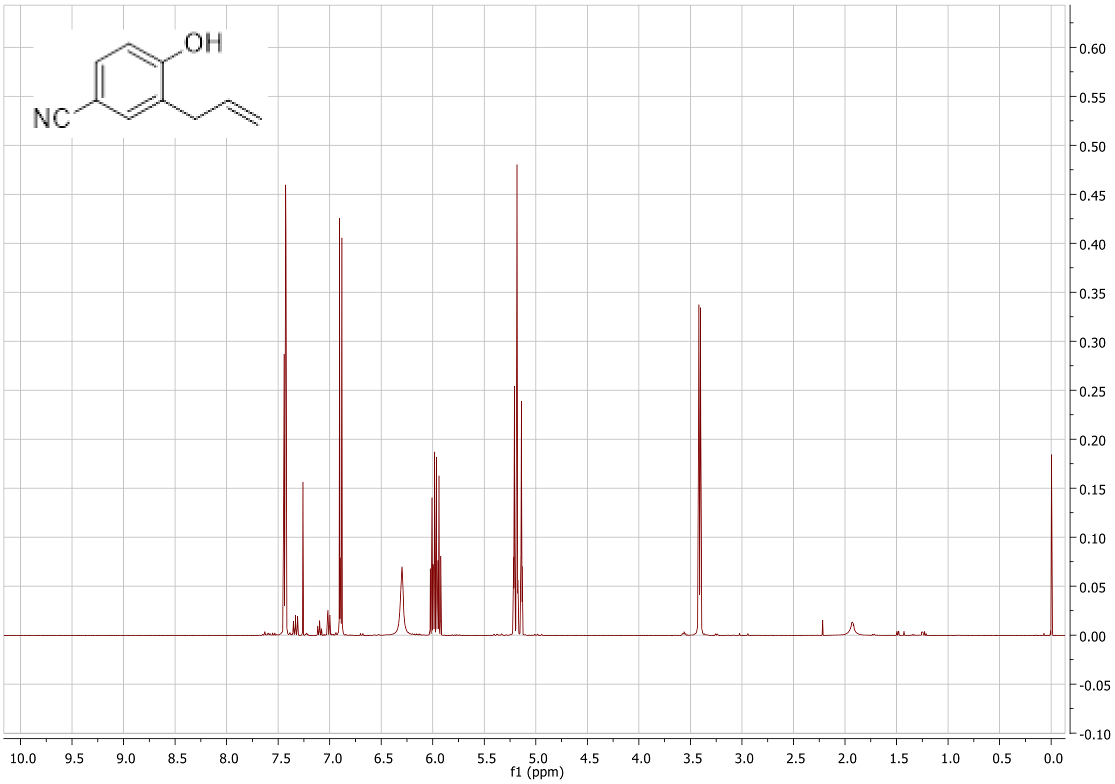

^{13}C NMR

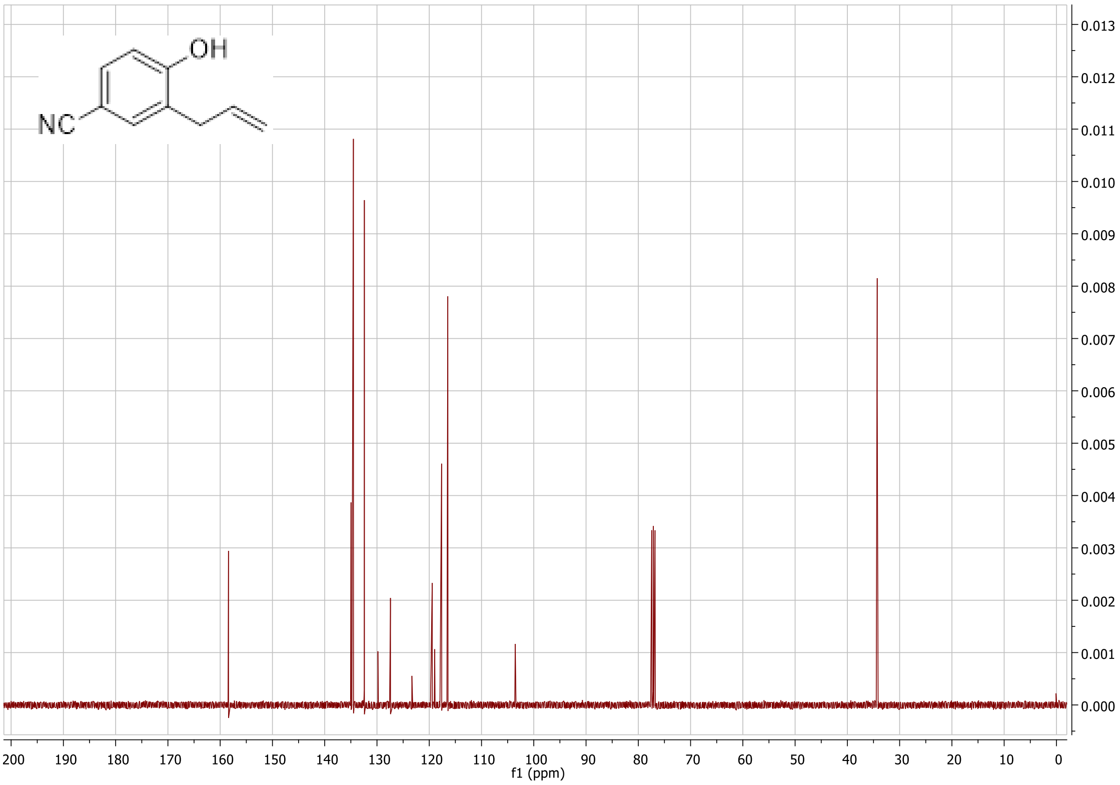

3d

^{1}H NMR

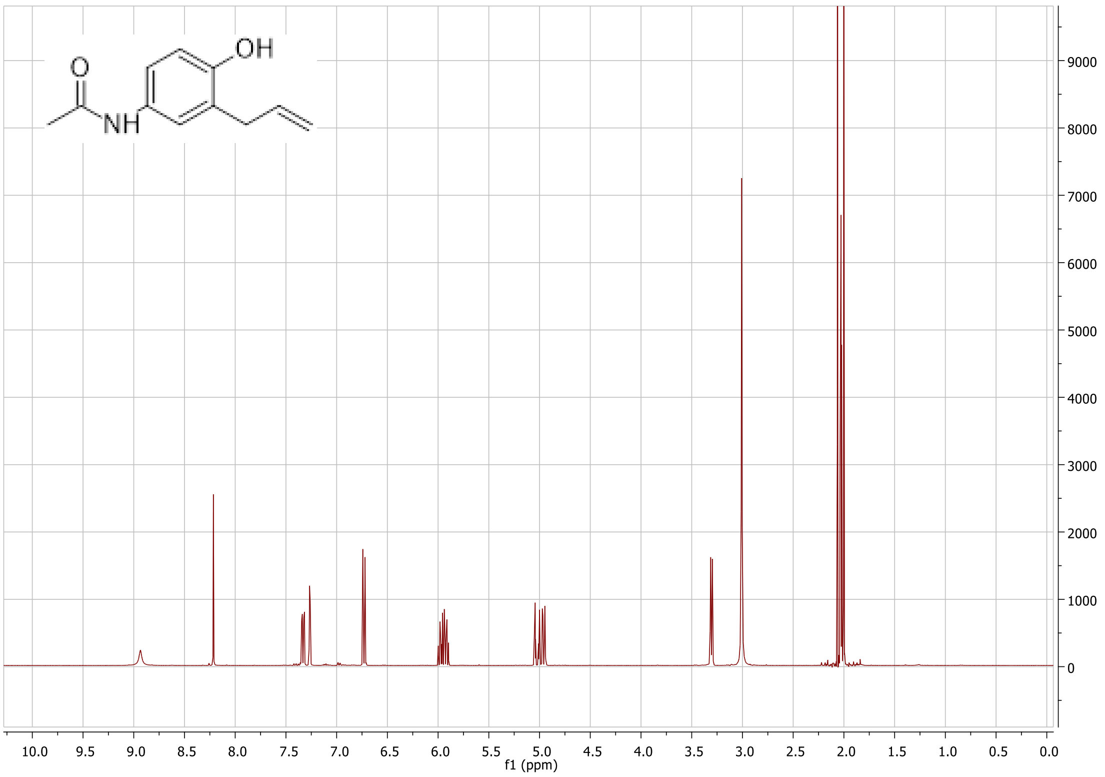

^{13}C NMR

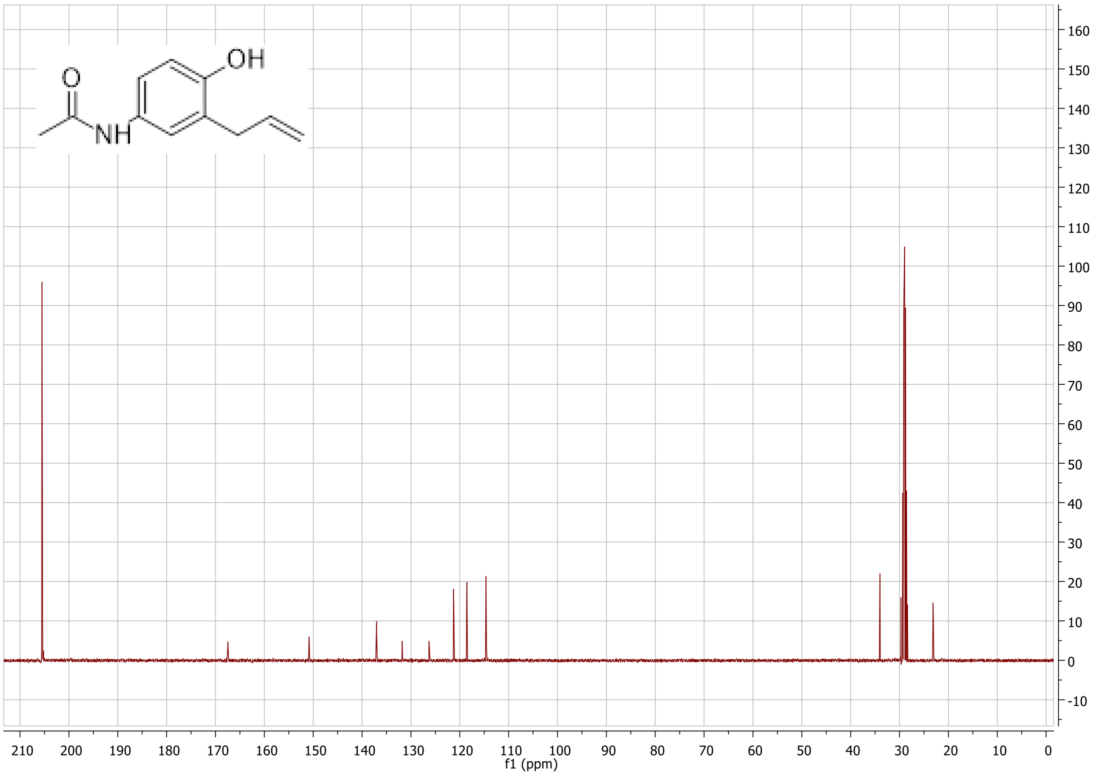

4a

^{1}H NMR

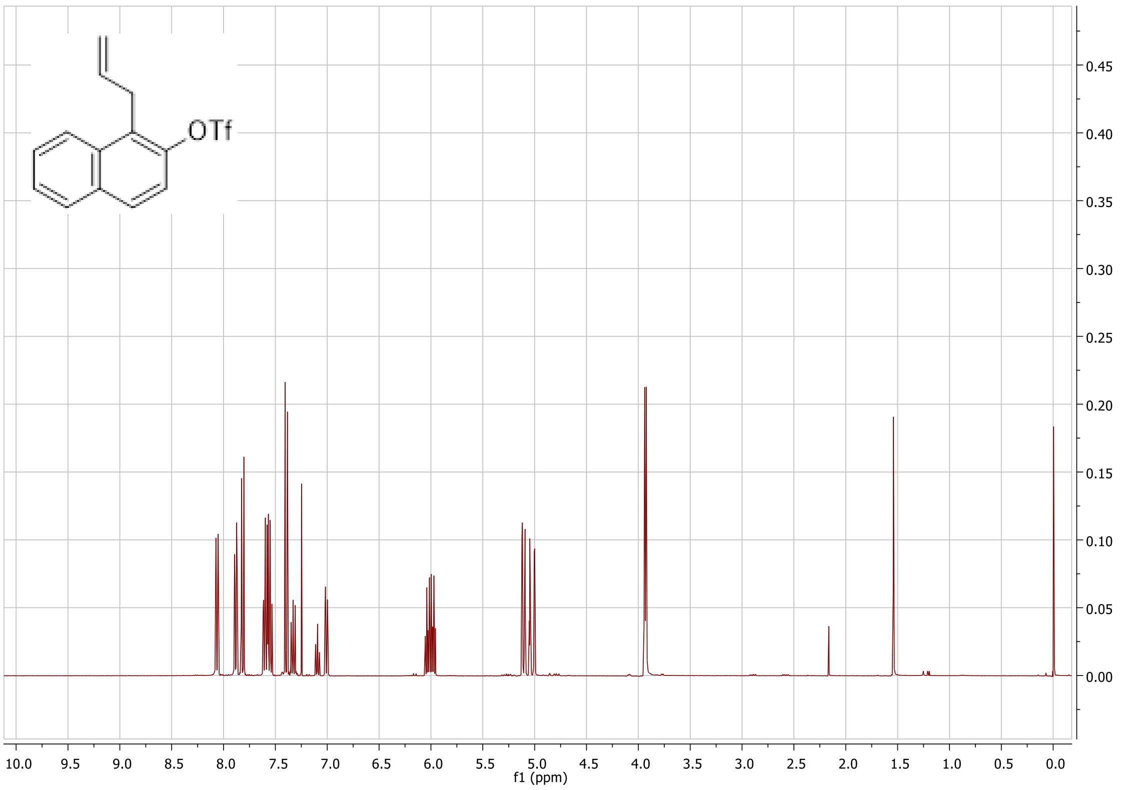

^{13}C NMR

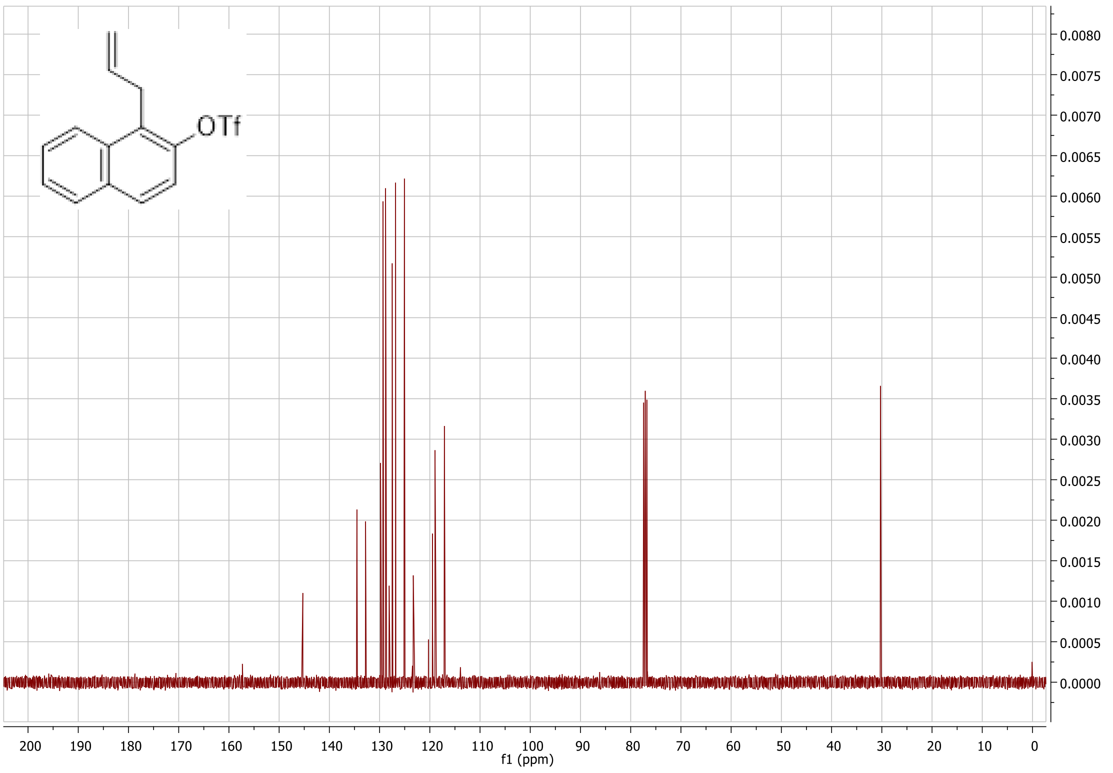

4b

^{1}H NMR

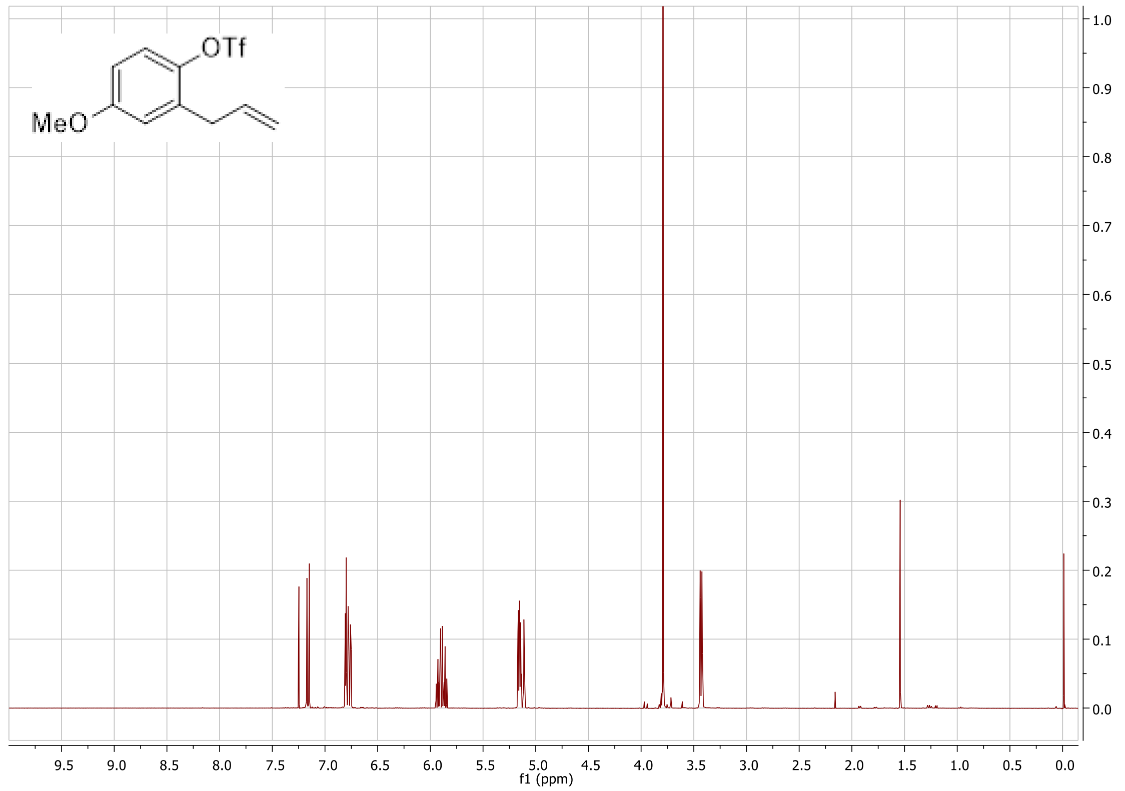

^{13}C NMR

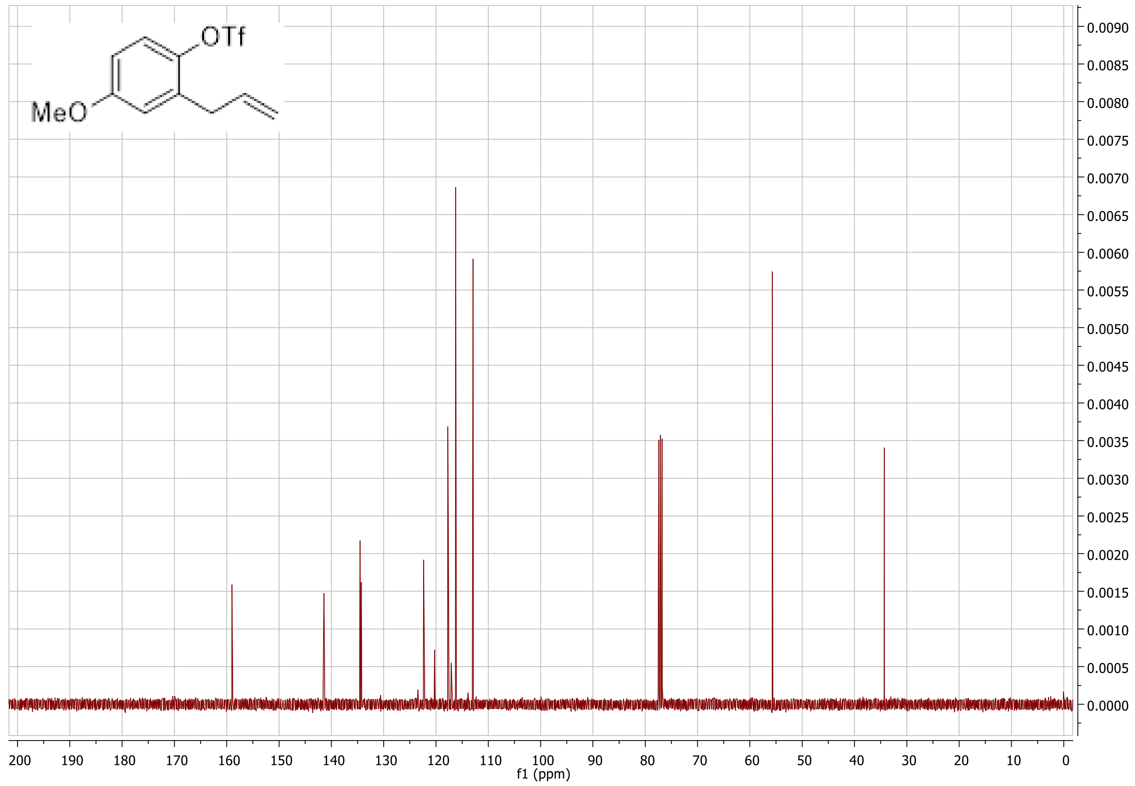

4c

^{1}H NMR

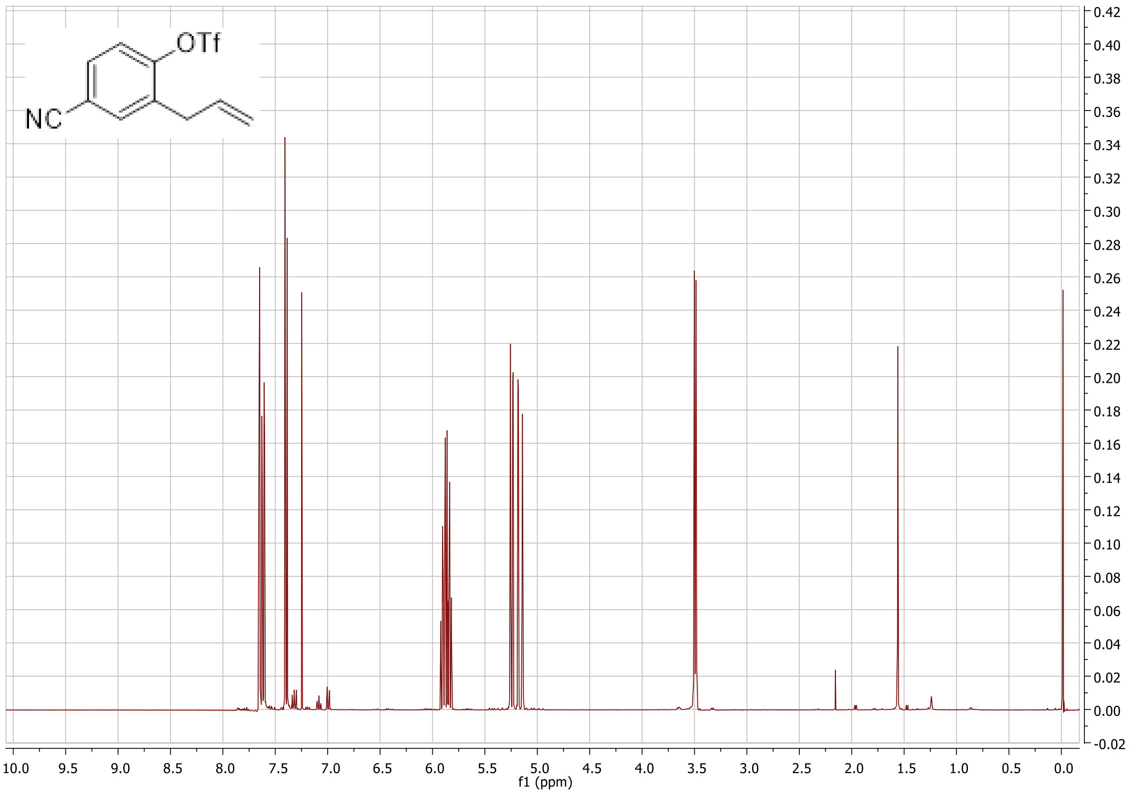

^{13}C NMR

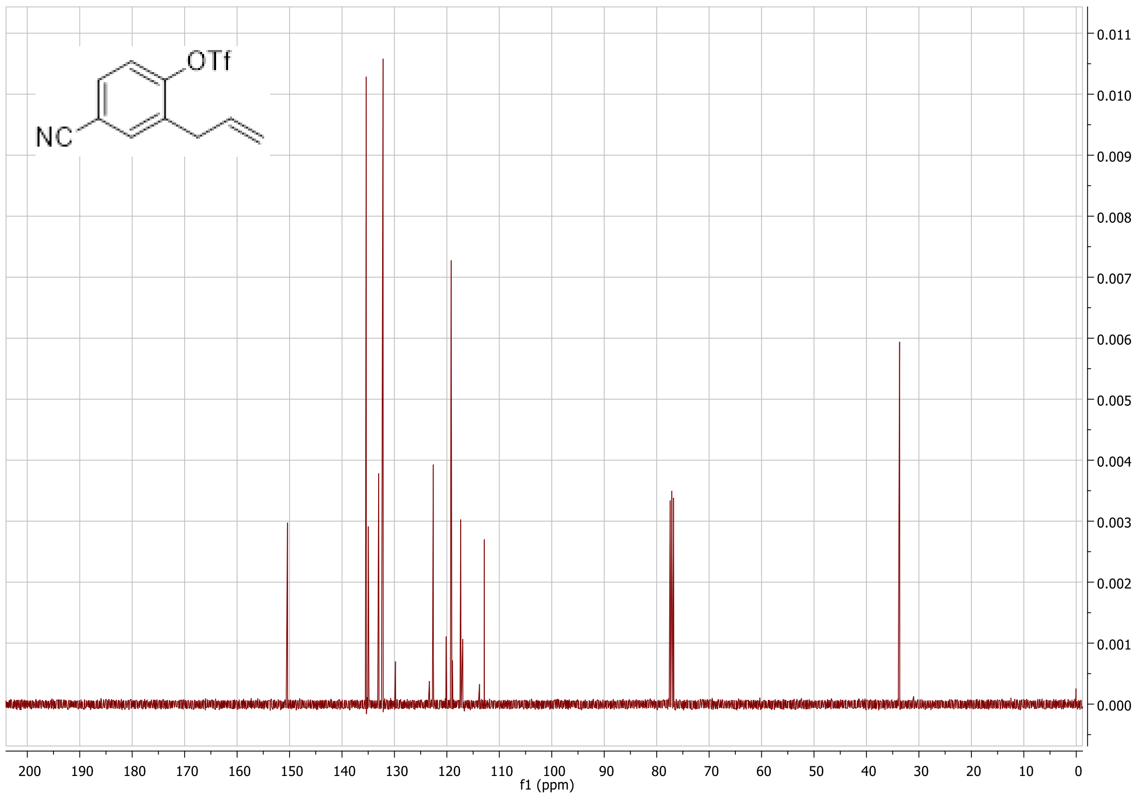

4d

^{1}H NMR

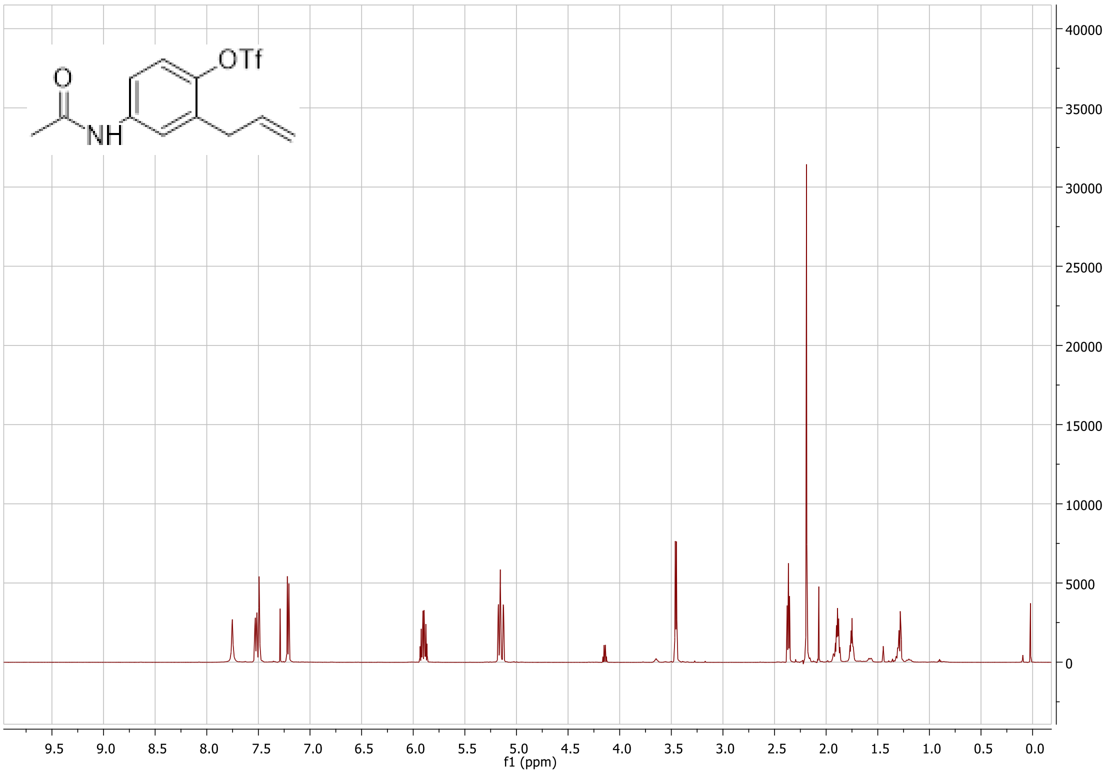

^{13}C NMR

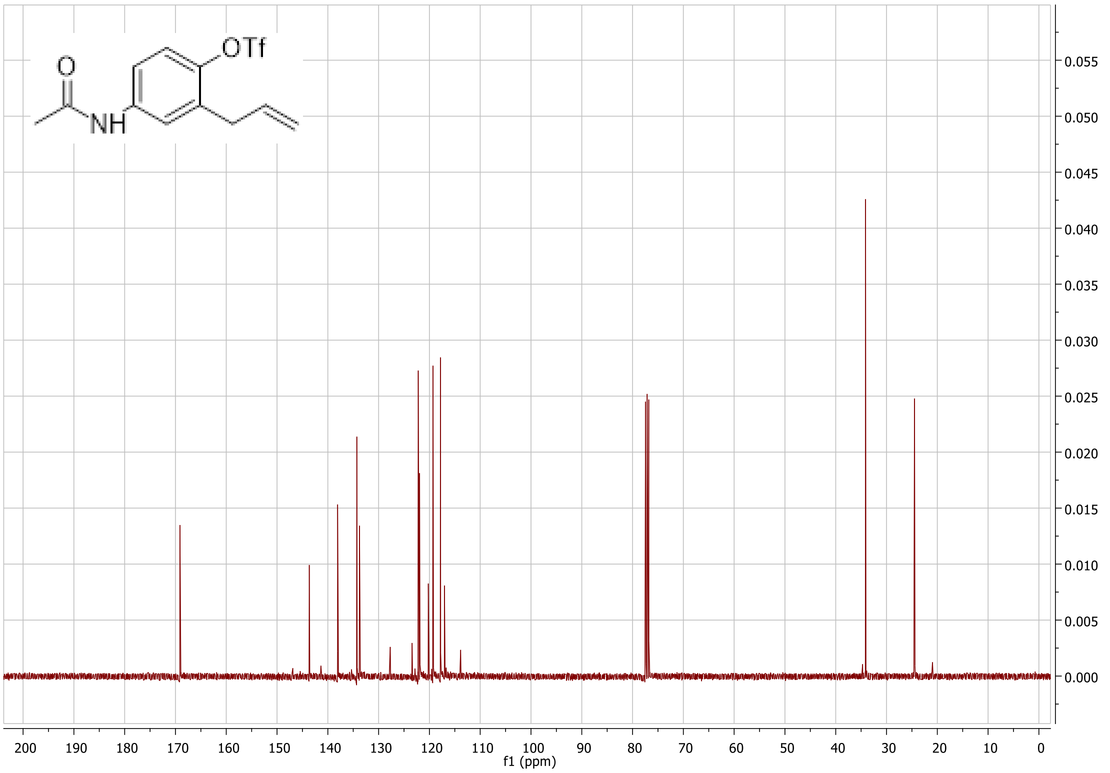

5a

^{1}H NMR

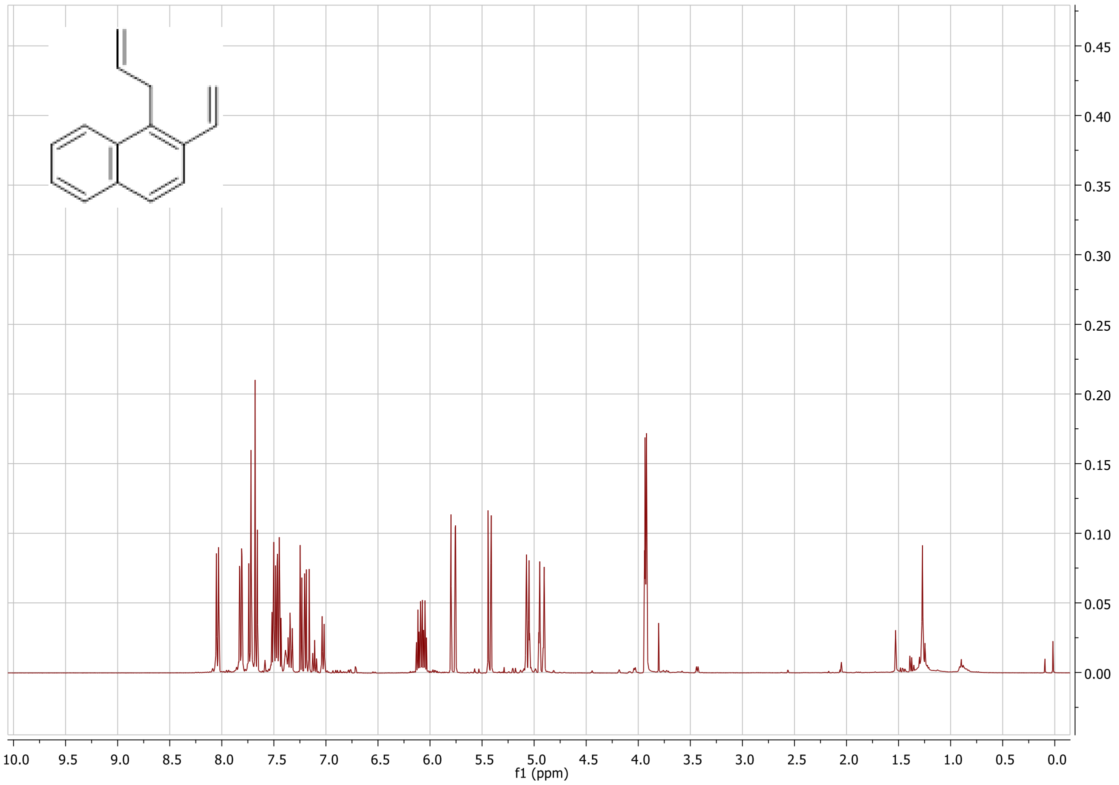

^{13}C NMR

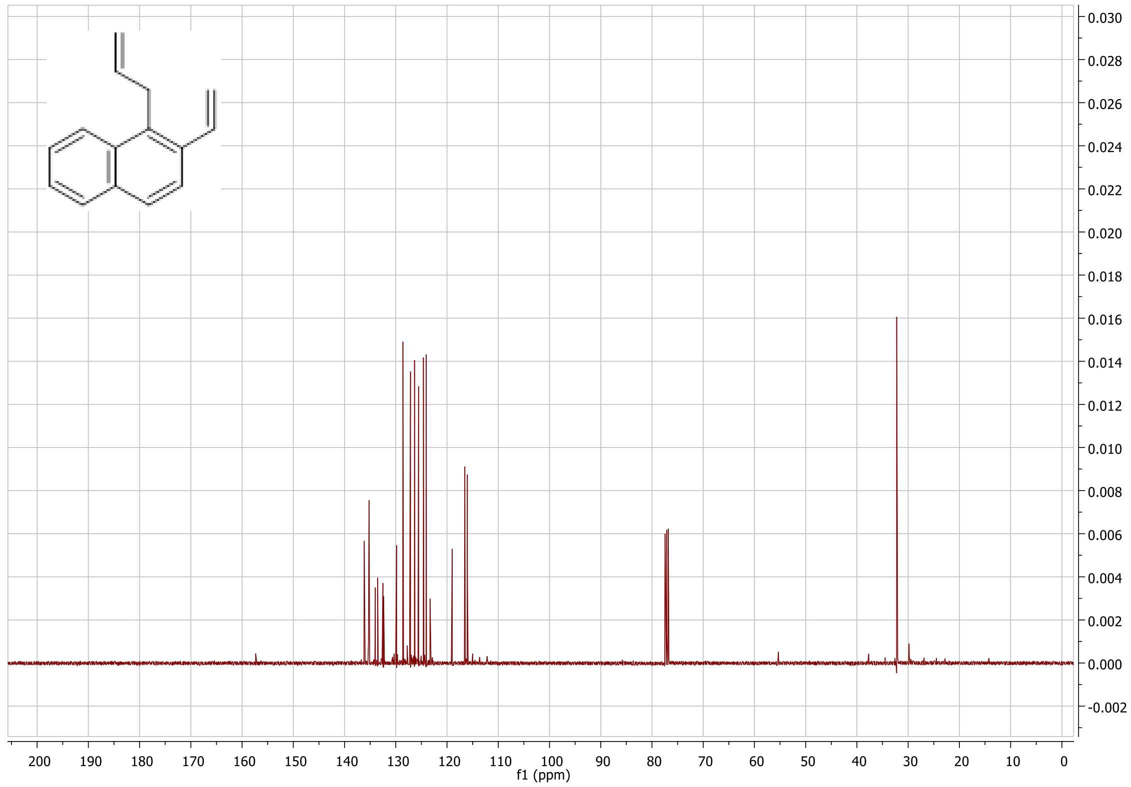

5b

^{1}H NMR

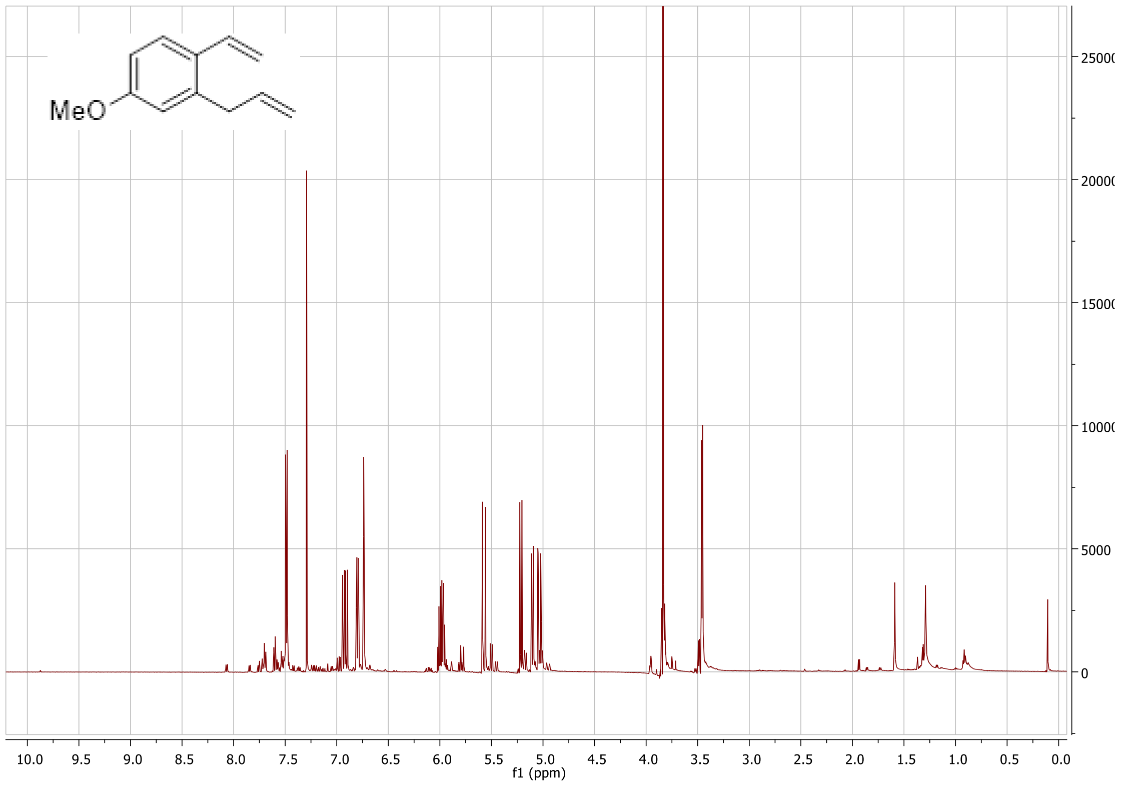

^{13}C NMR

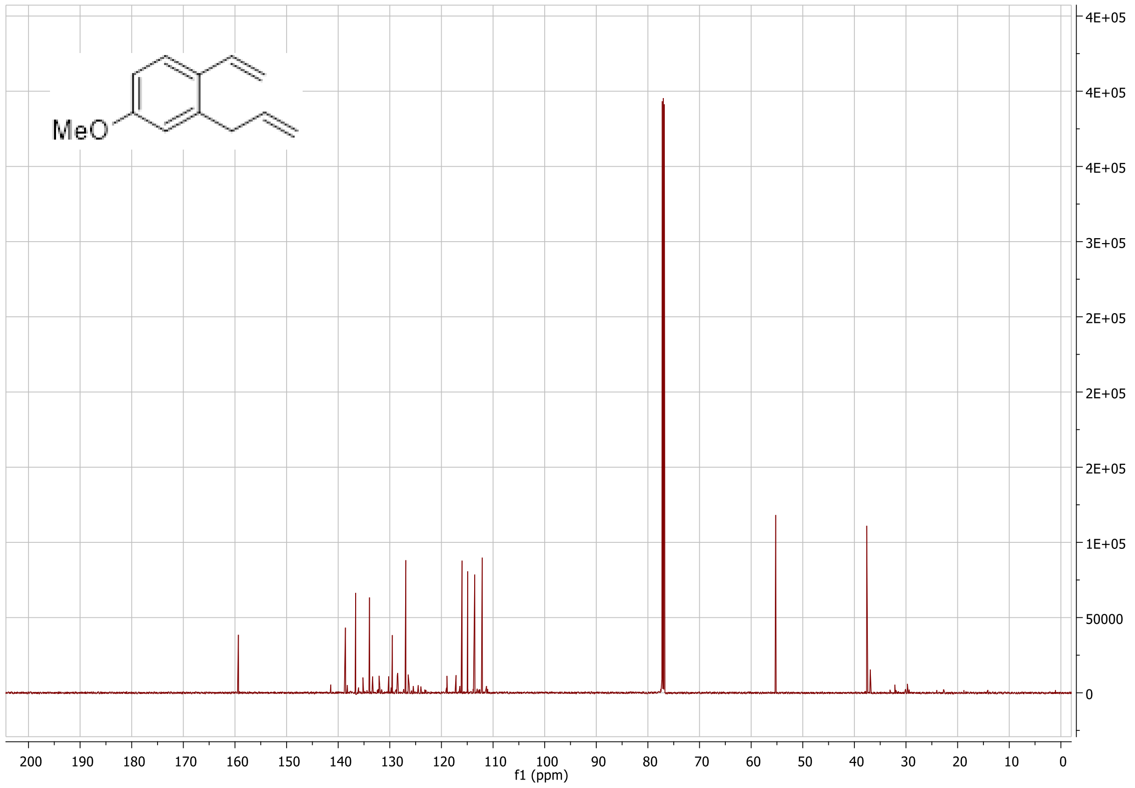

5c

^{1}H NMR

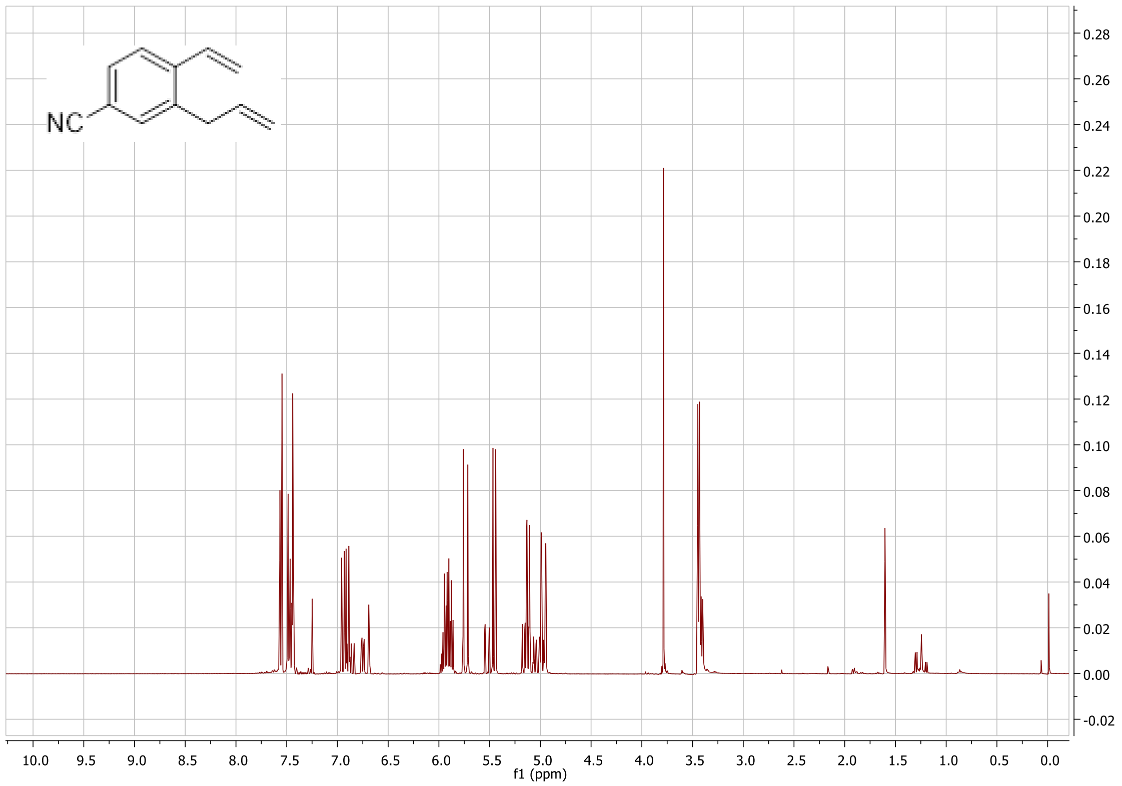

^{13}C NMR

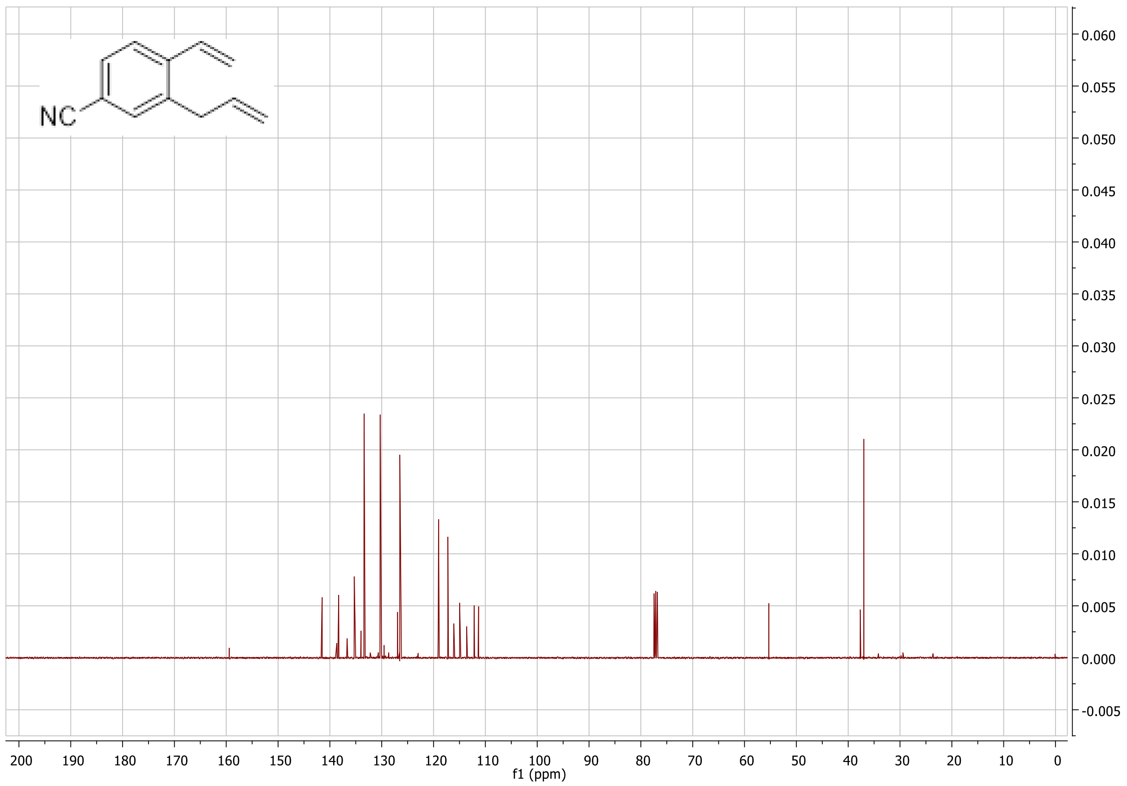

5d

^{1}H NMR

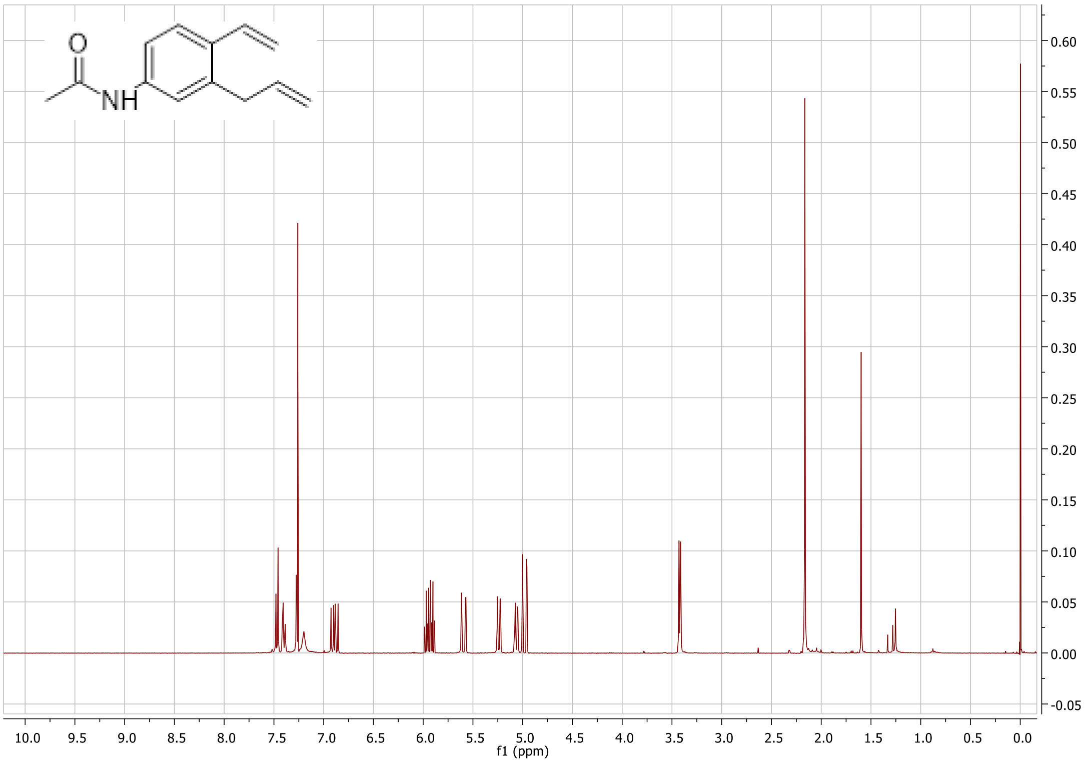

^{13}C NMR

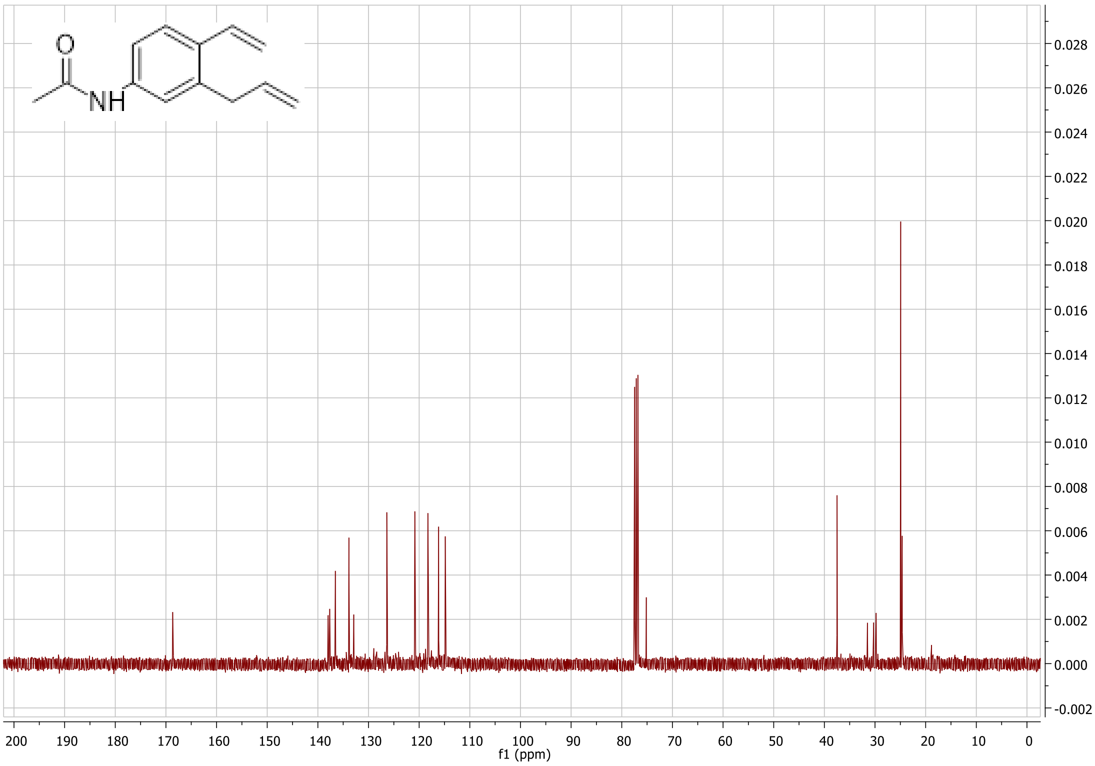

5e

^{1}H NMR

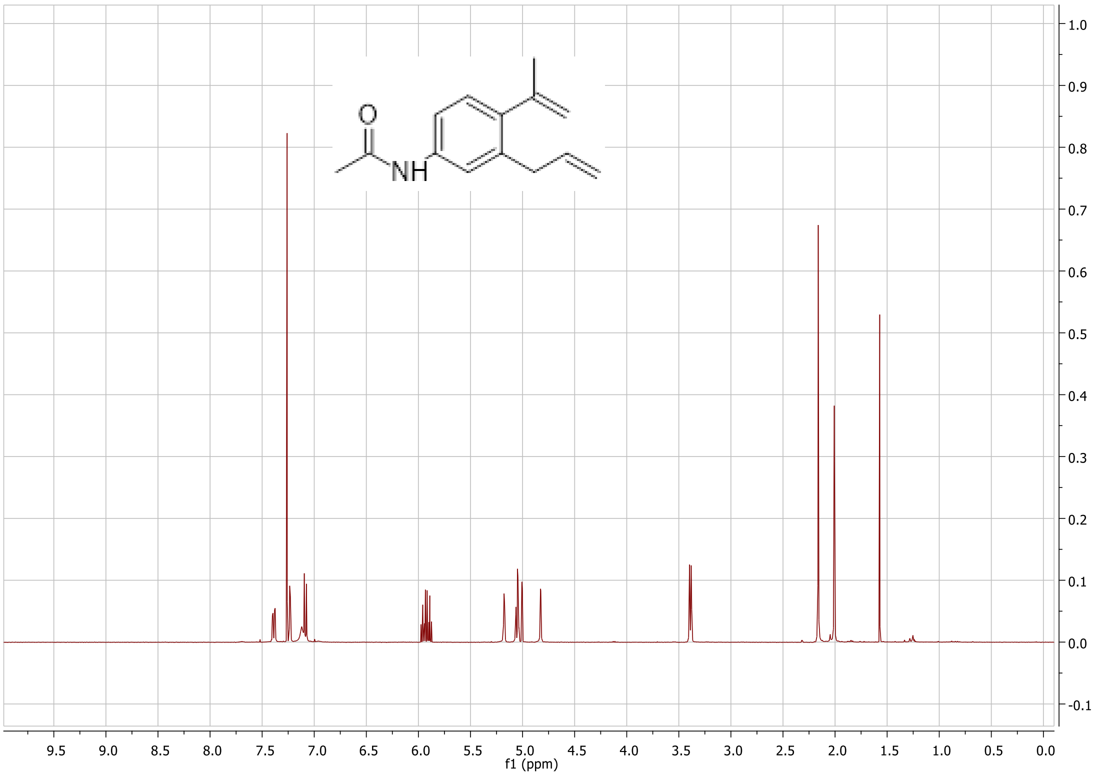

^{13}C NMR

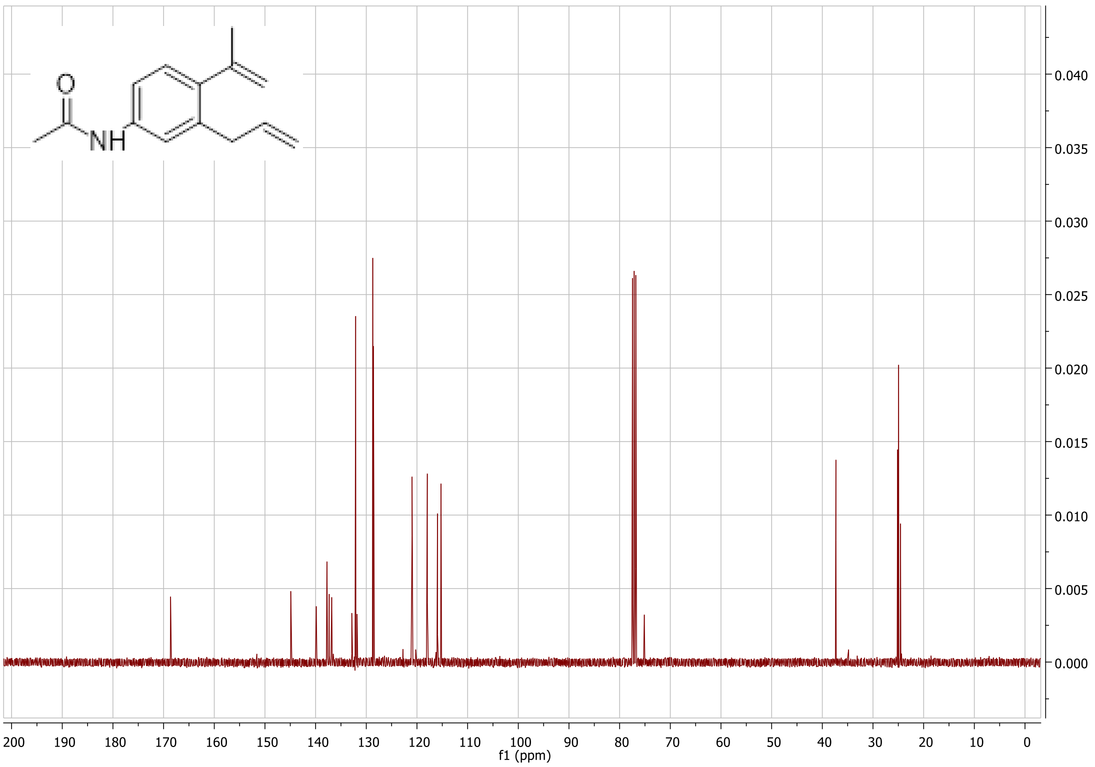

6a

^{1}H NMR

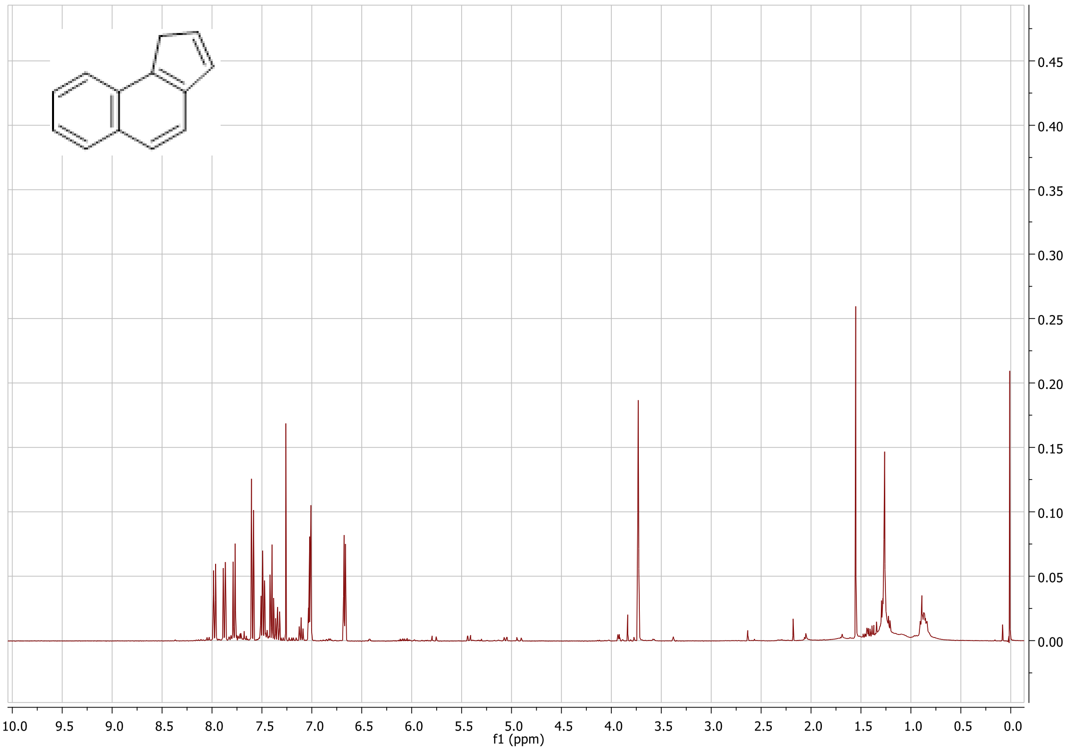

^{13}C NMR

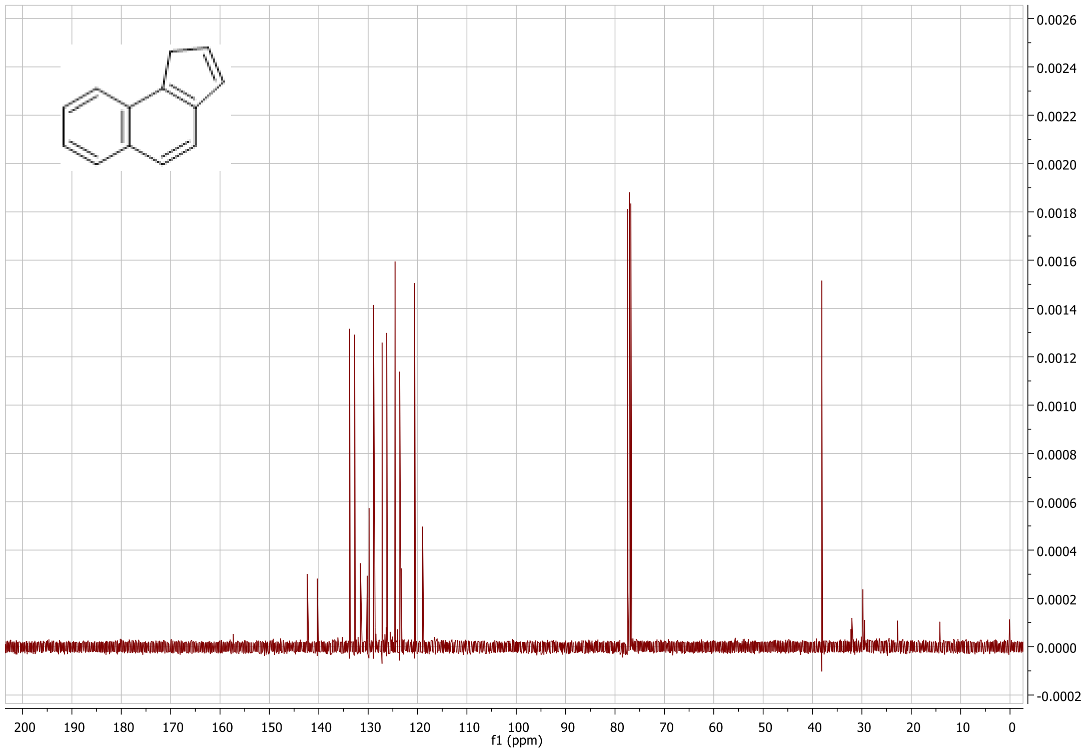

6b

^{1}H NMR

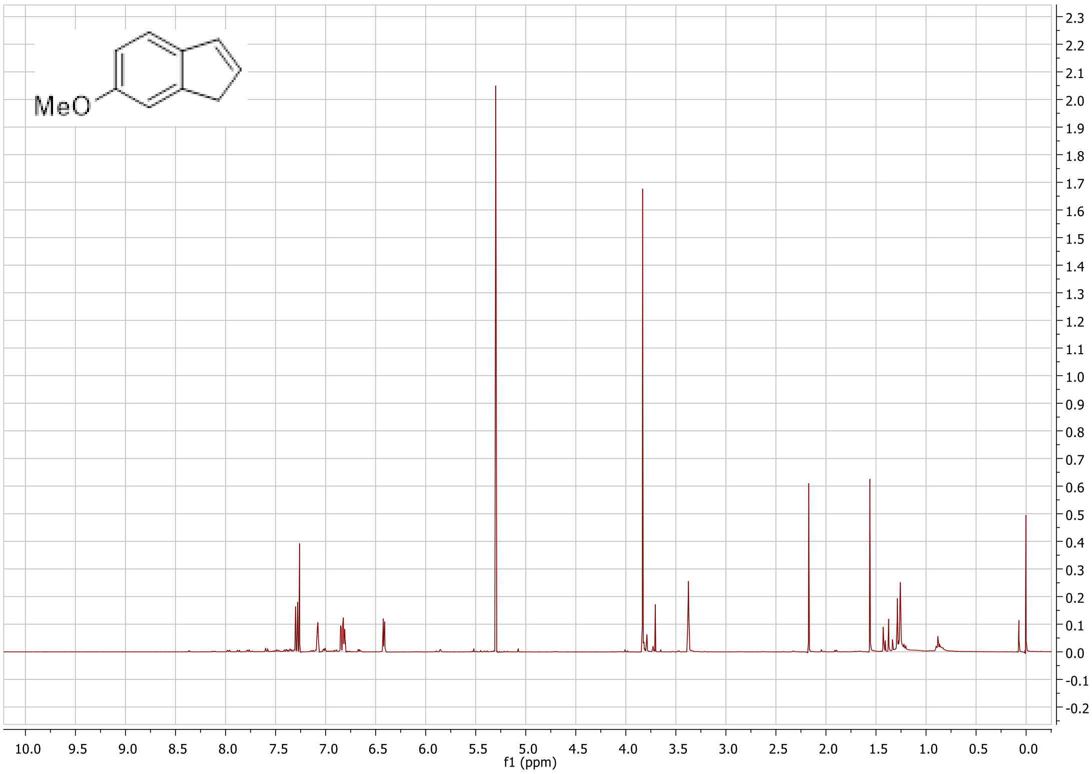

^{13}C NMR

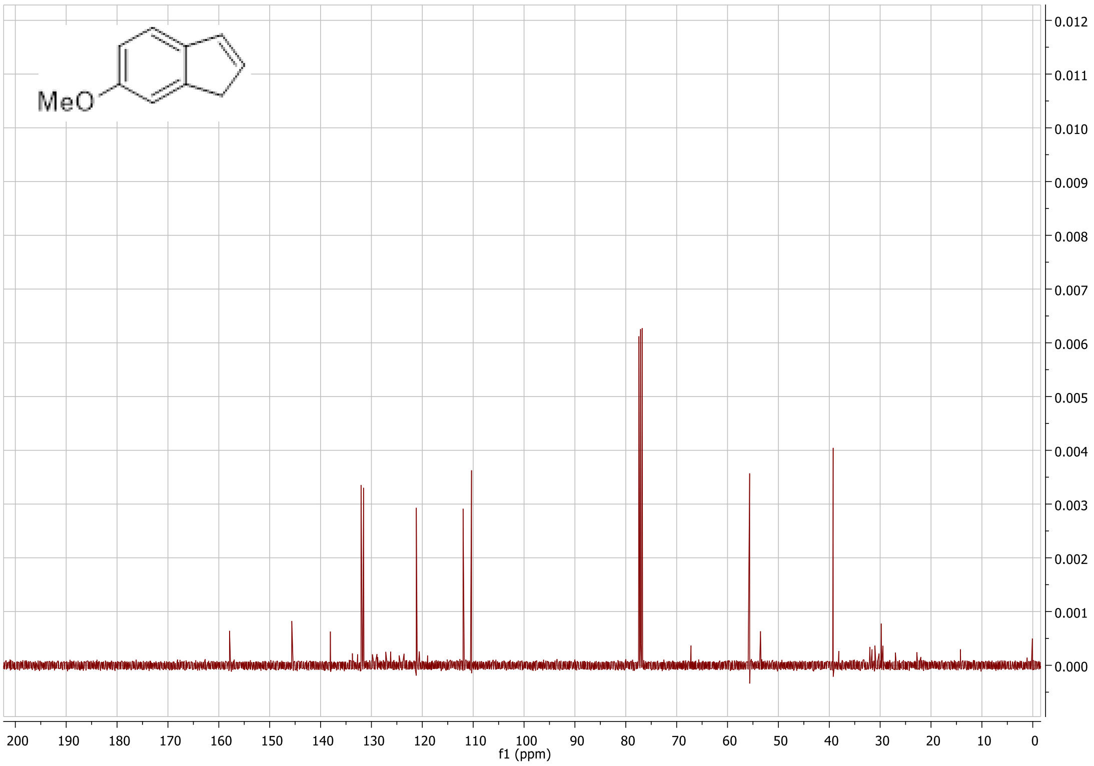

6c

^{1}H NMR

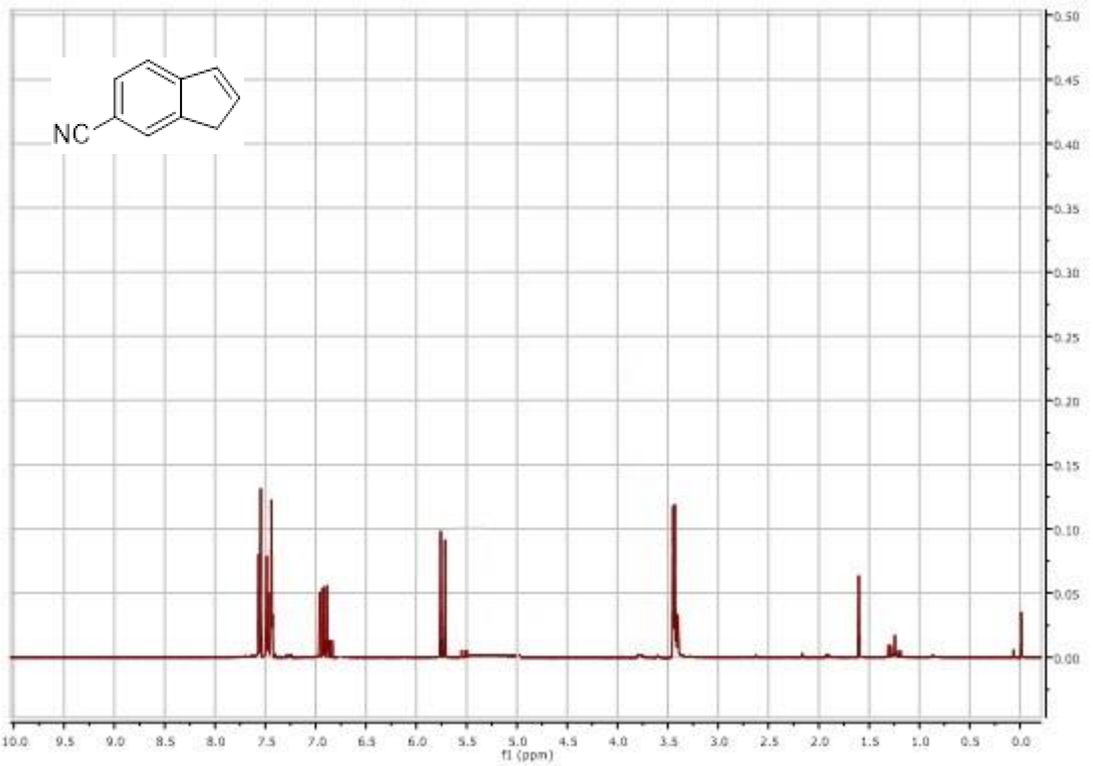

^{13}C NMR

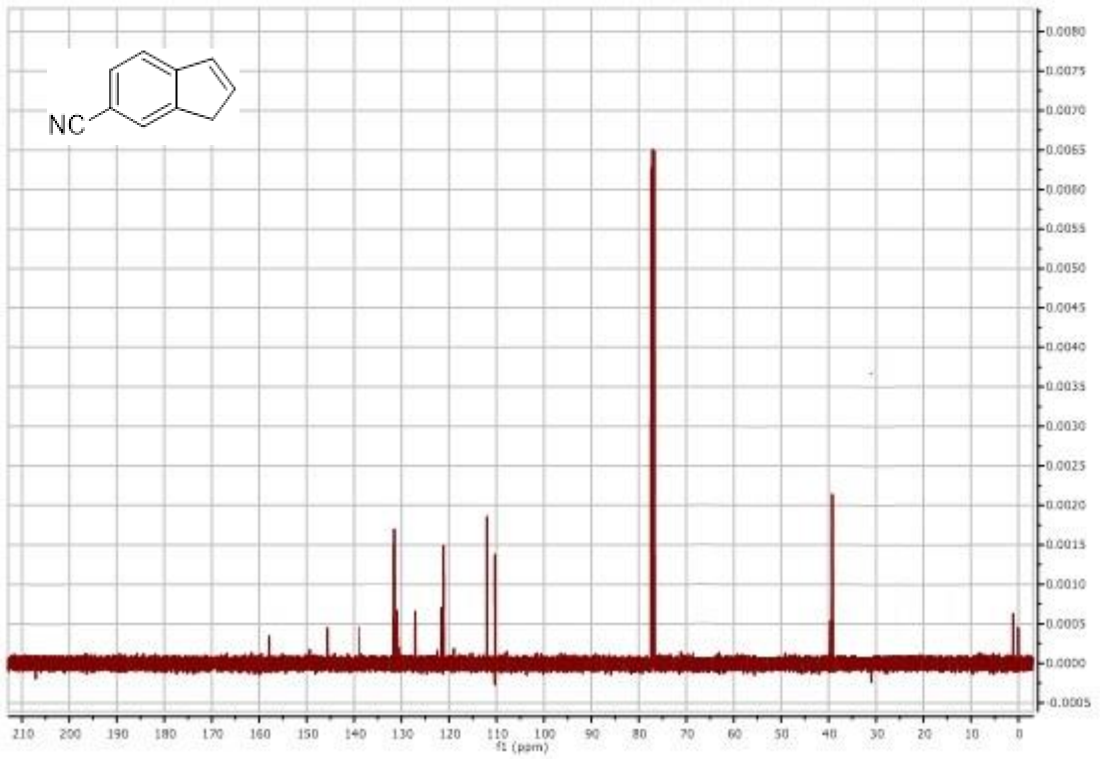

6d

^{1}H NMR

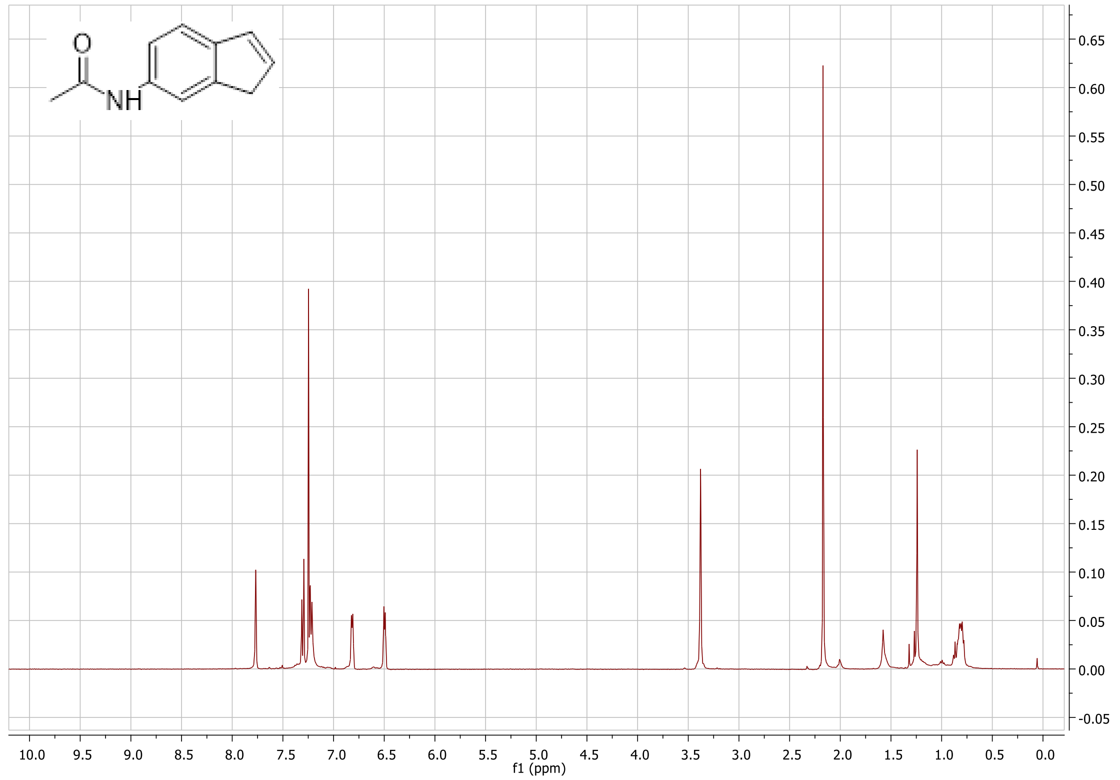

^{13}C NMR

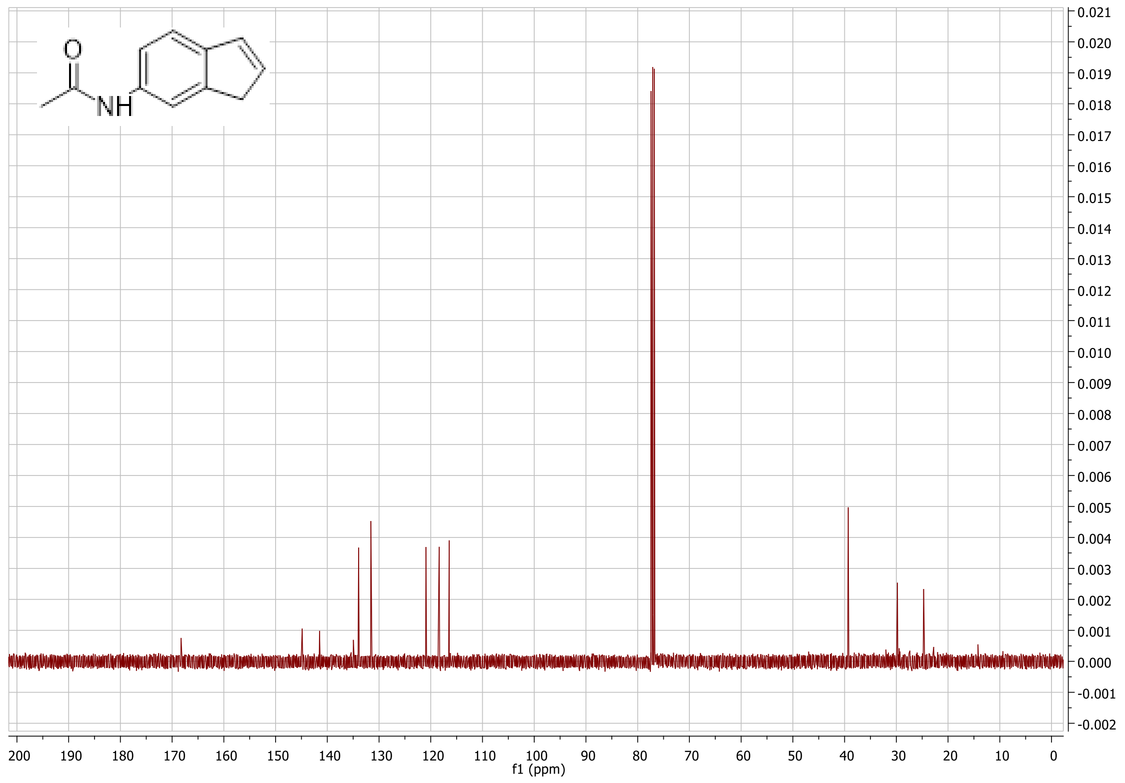

6e

^{1}H NMR

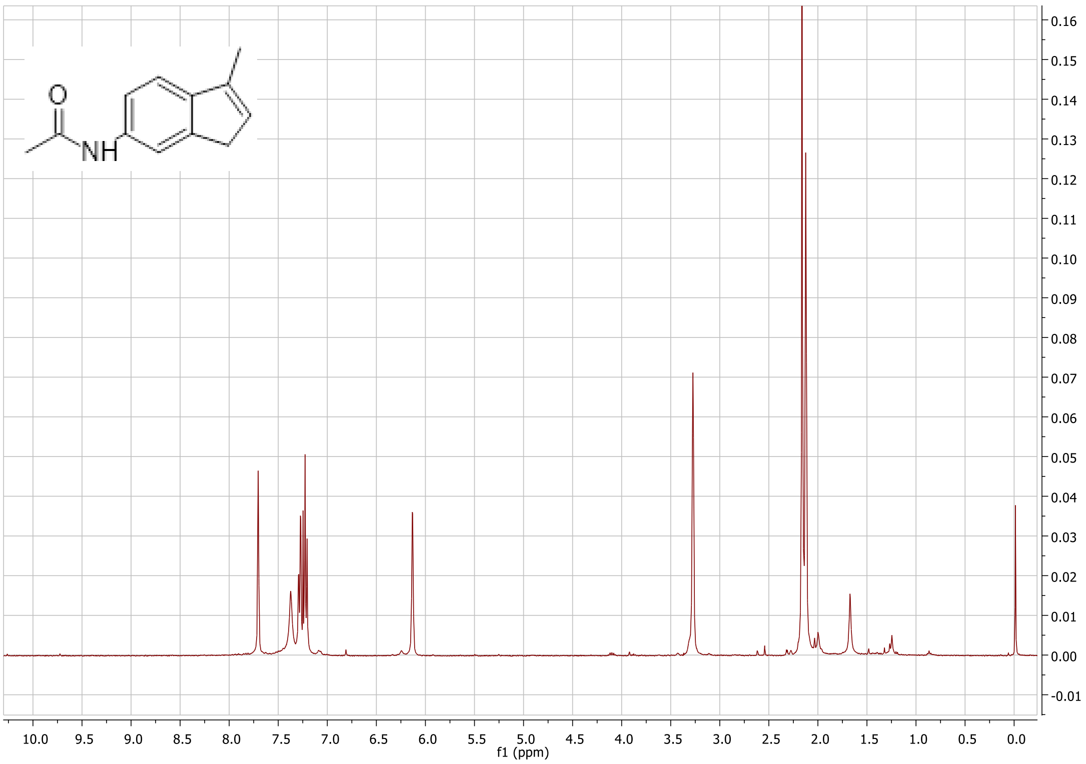

^{13}C NMR

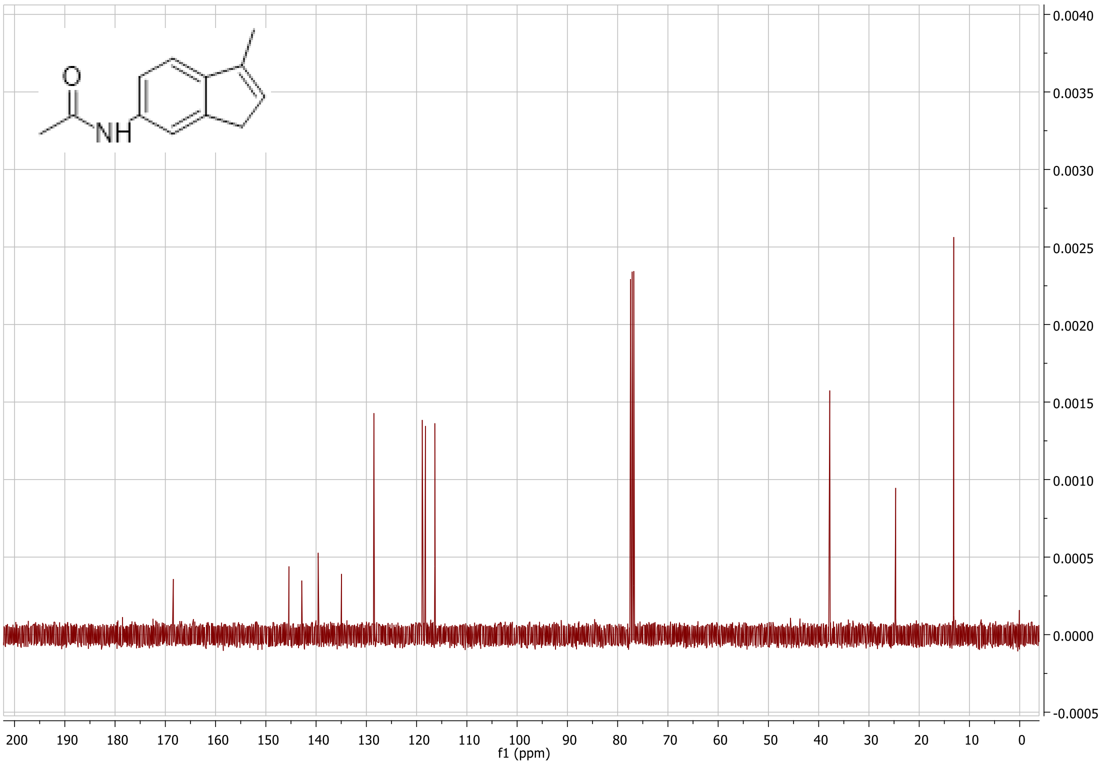

5a, EI-MS *m/z*: $[M]^+$ Calcd for $C_{15}H_{14}$ 194.28; Found: 194.1

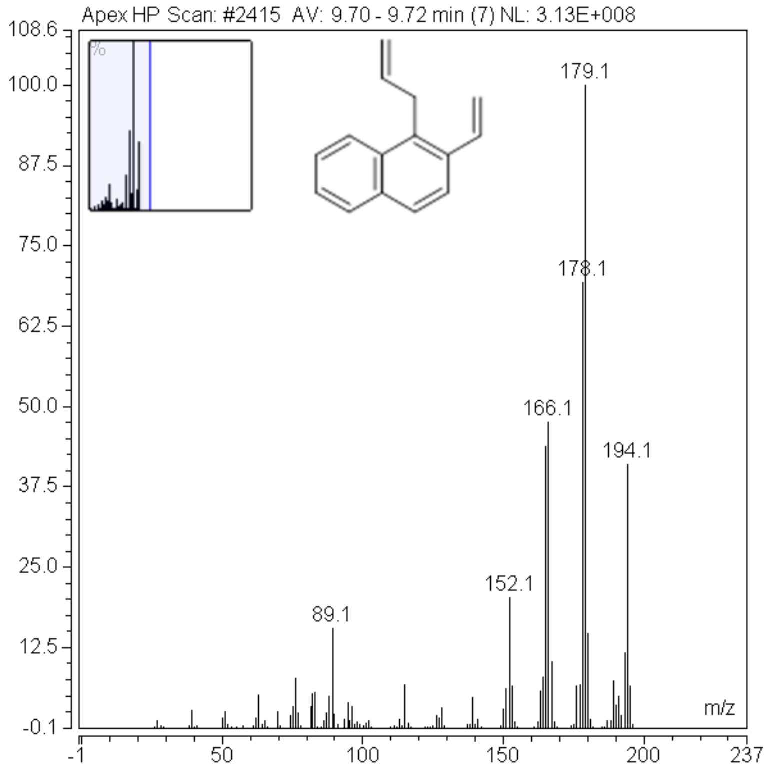

5b, EI-MS *m/z*: $[M]^+$ Calcd for $C_{12}H_{14}O$ 174.24; Found: 174.1

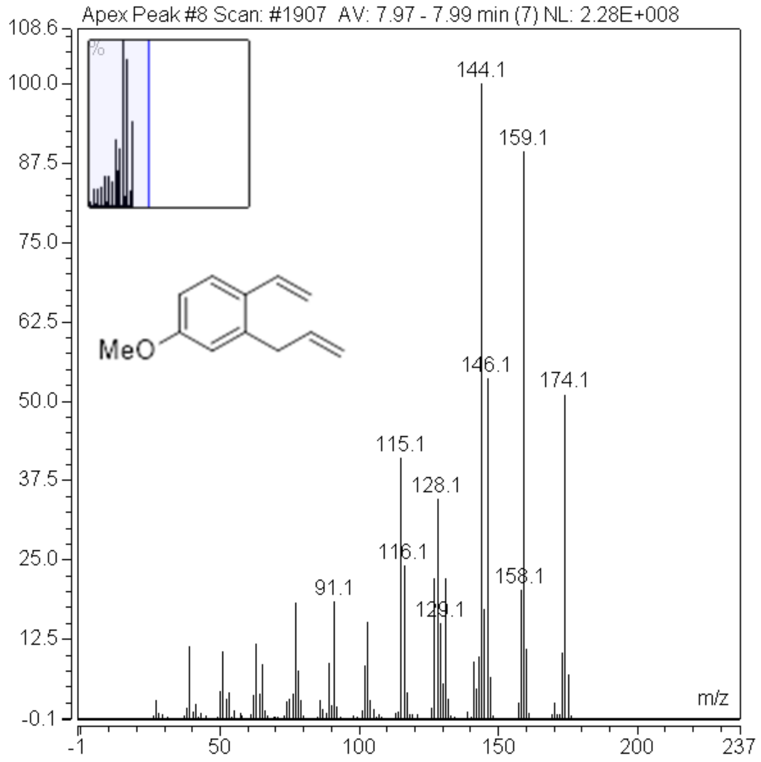

5c, EI-MS *m/z*: $[M]^+$ Calcd for $C_{12}H_{11}N$ 169.23; Found: 169.1

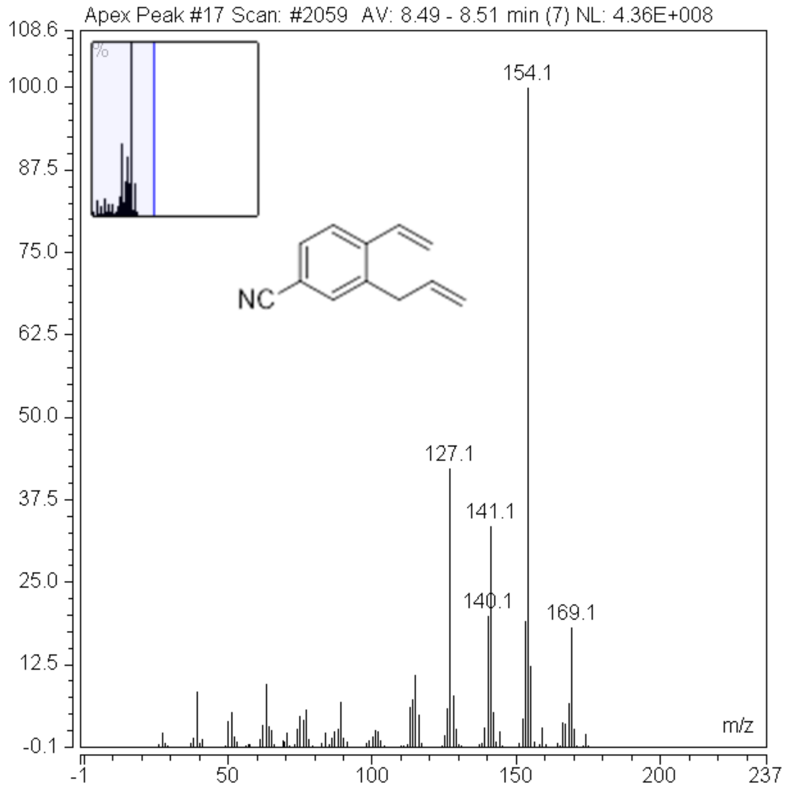

5d, EI-MS *m/z*: $[M]^+$ Calcd for $C_{13}H_{15}NO$ 201.27; Found: 201.1

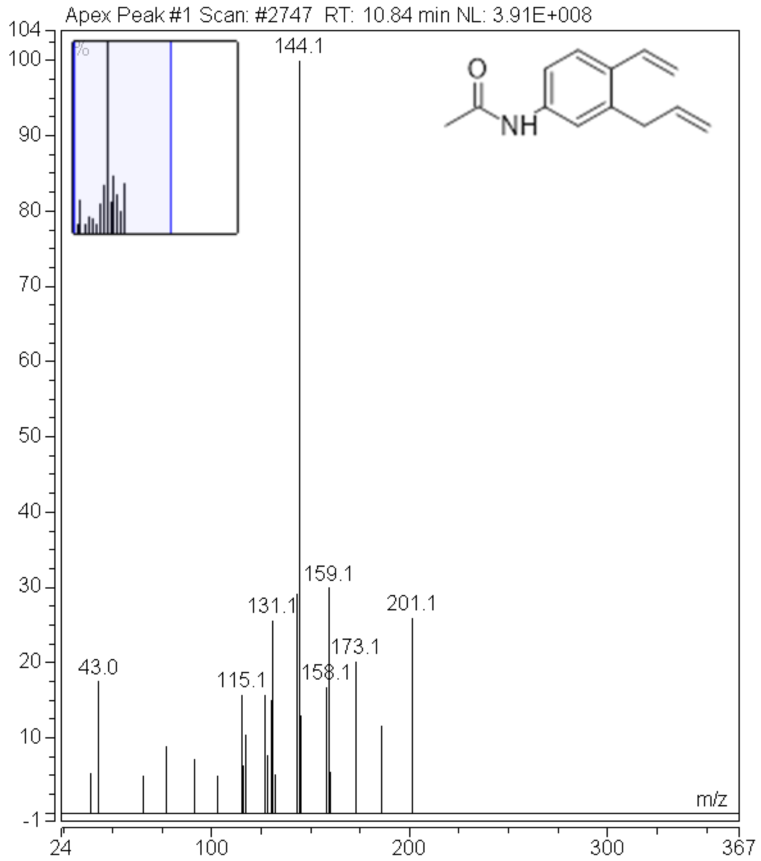

5e, EI-MS *m/z*: $[M]^+$ Calcd for $C_{14}H_{17}NO$ 215.30; Found: 215.1

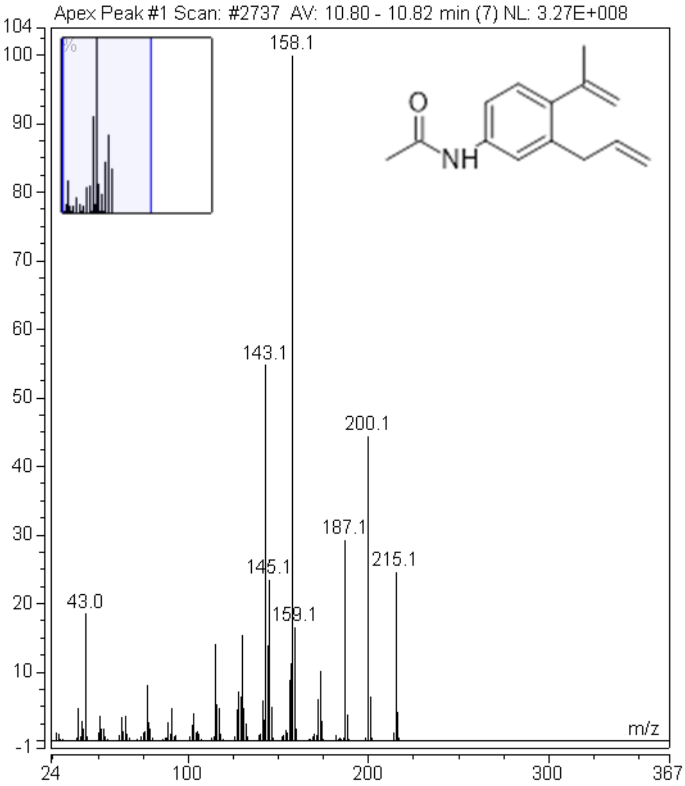

6a, EI-MS *m/z*: $[M]^+$ Calcd for $C_{13}H_{10}$ 166.22; Found: 166.1

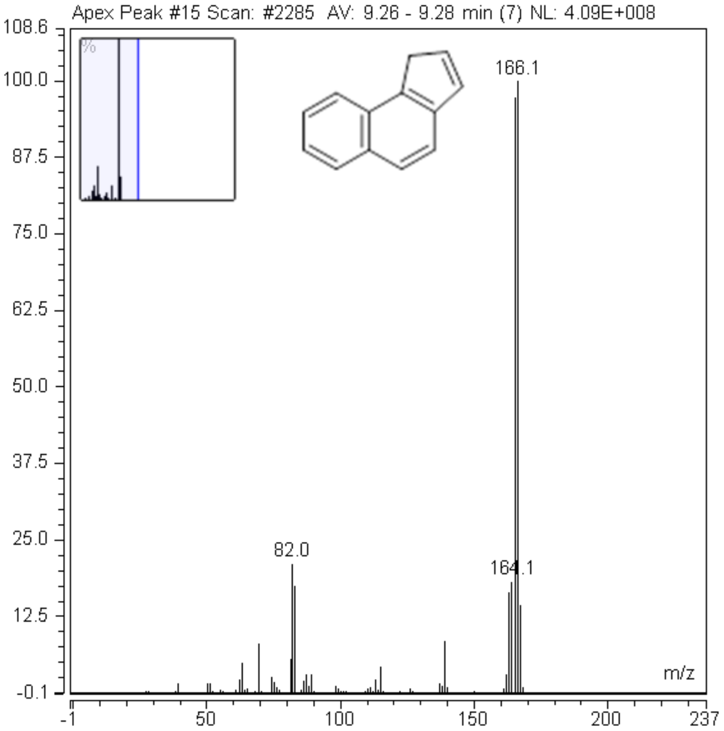

6b, EI-MS *m/z*: $[M]^+$ Calcd for $C_{10}H_{10}O$ 146.19; Found: 146.1

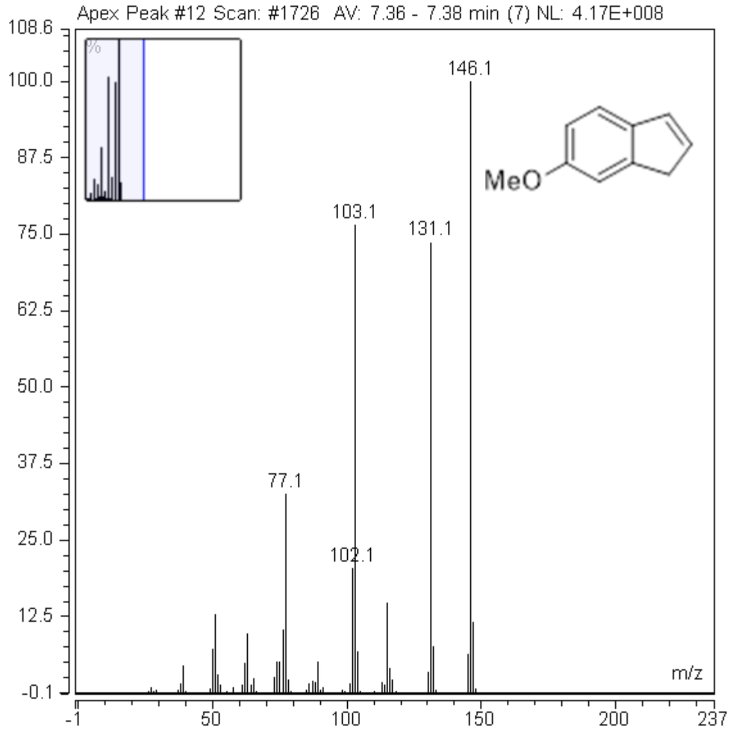

6c, EI-MS *m/z*: $[M]^+$ Calcd for $C_{10}H_7N$ 141.17; Found: 141.1

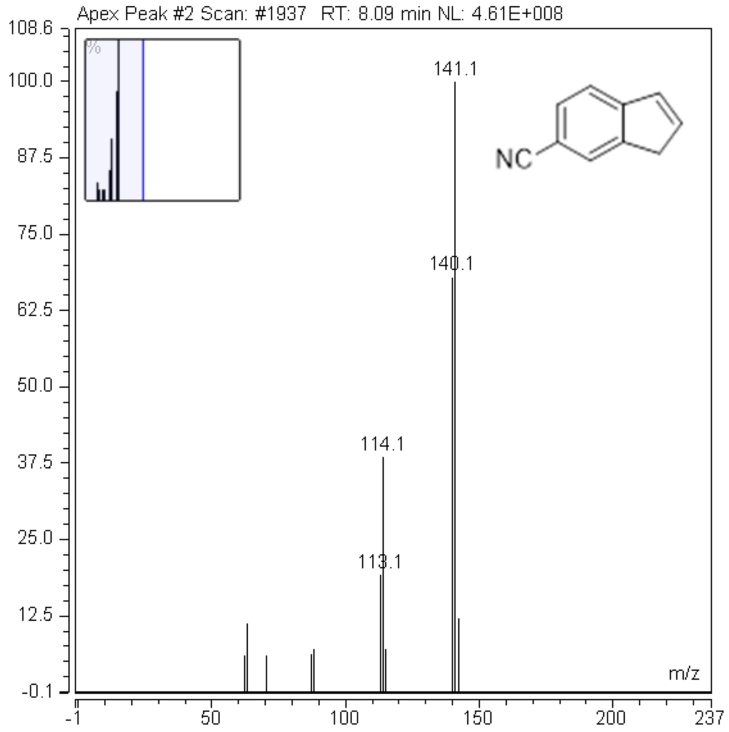

6d, EI-MS *m/z*: $[M]^+$ Calcd for $C_{11}H_{11}NO$ 173.22; Found: 173.1

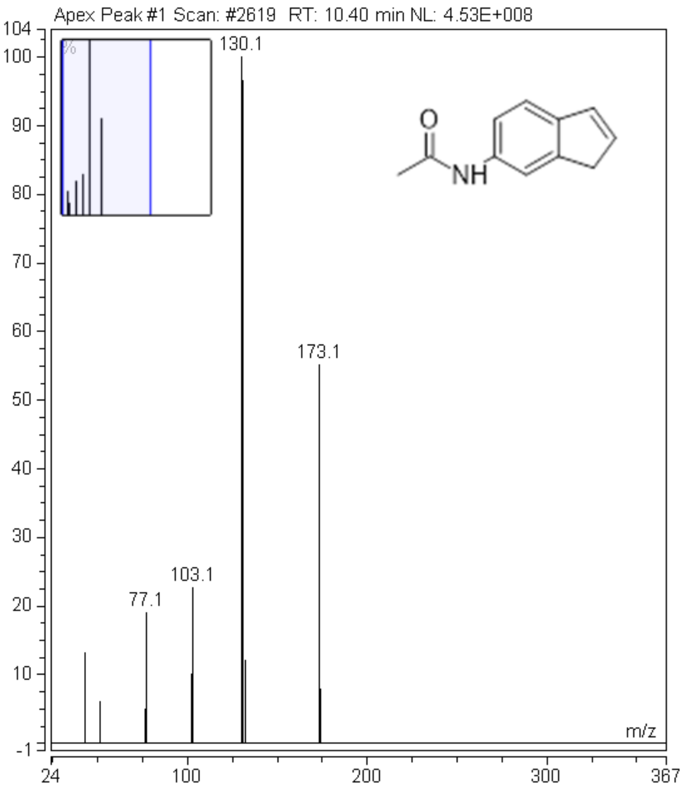

6e, EI-MS *m/z*: $[M]^+$ Calcd for $C_{12}H_{13}NO$ 187.24; Found: 187.1

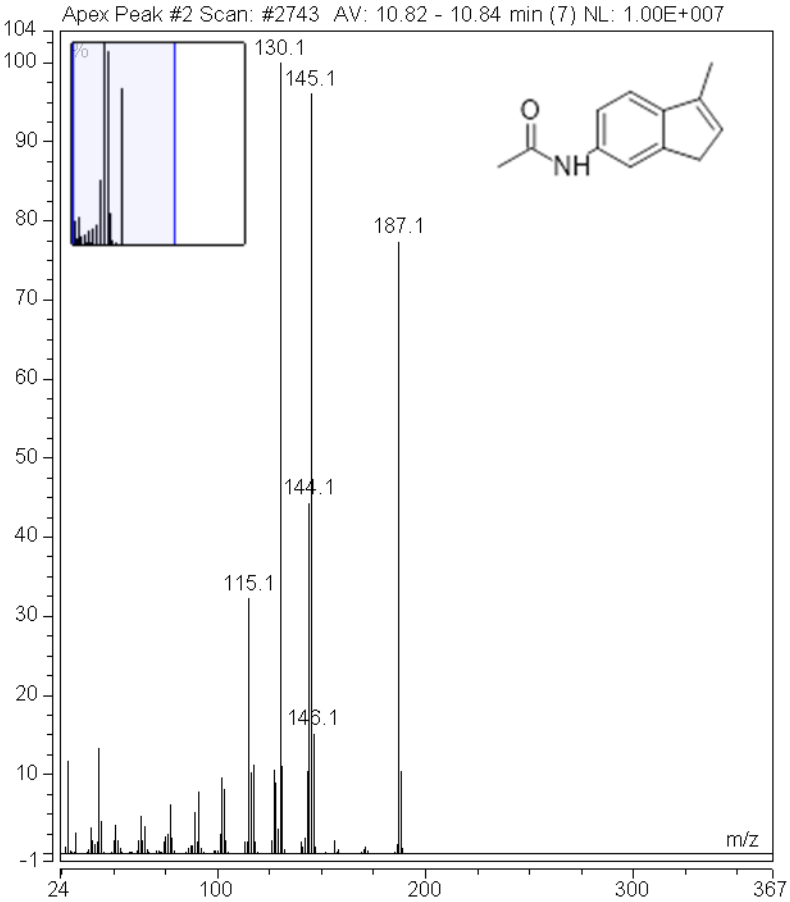

NMR and HR-MS Spectra for indene-fullerene adducts

Benzo-ICMA

^{1}H NMR

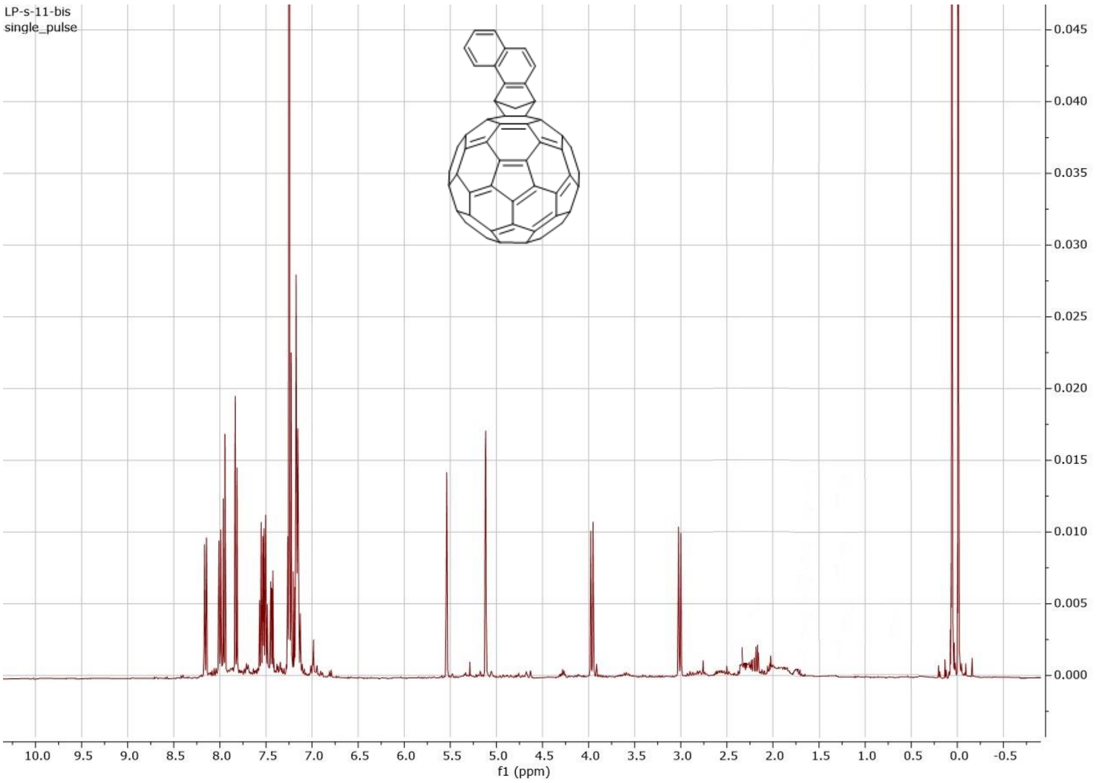

^{13}C NMR

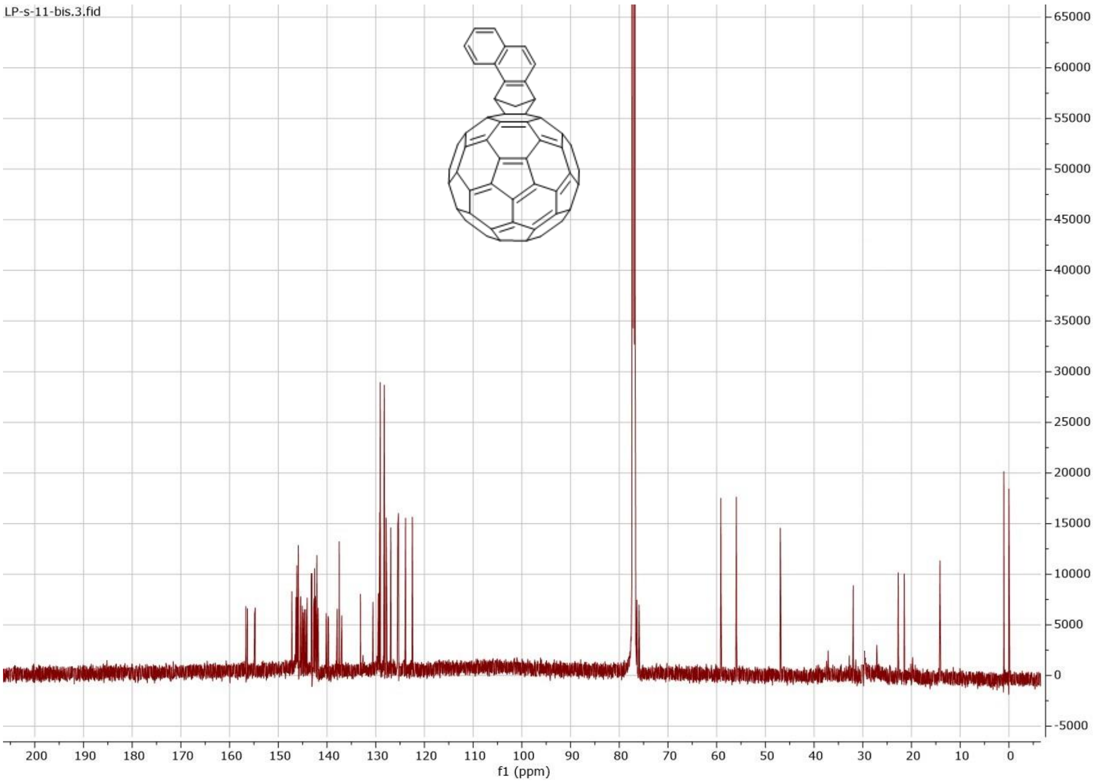

CN-ICMA

^{1}H NMR

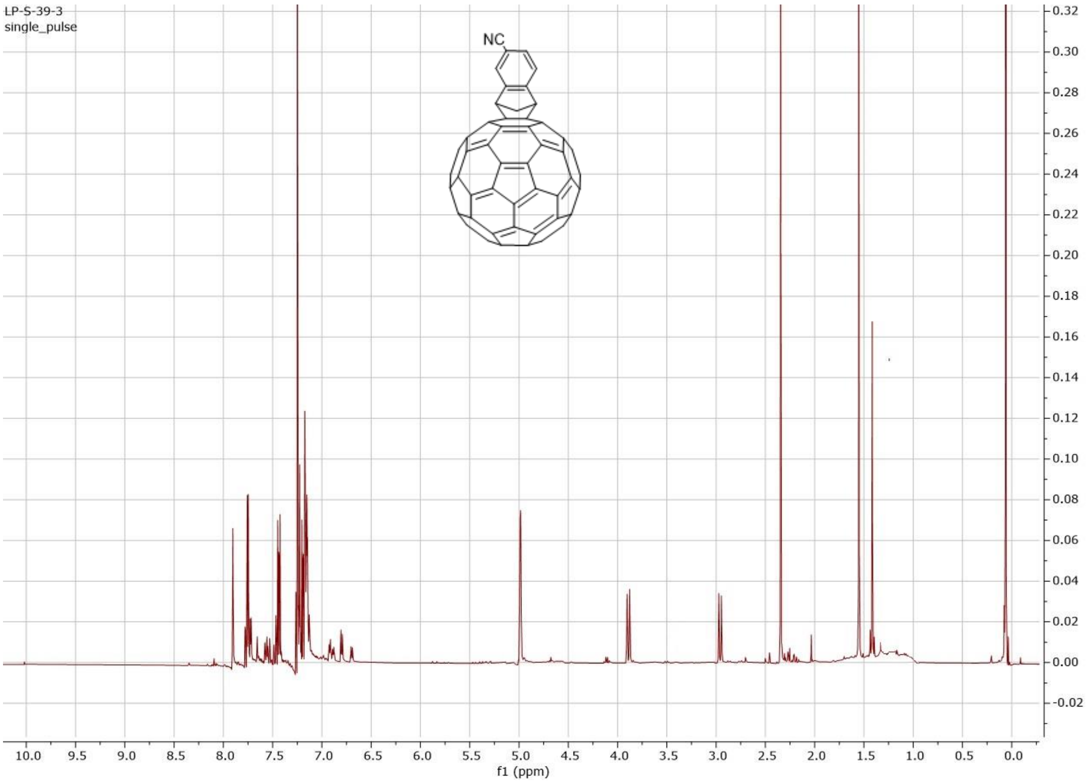

^{13}C NMR

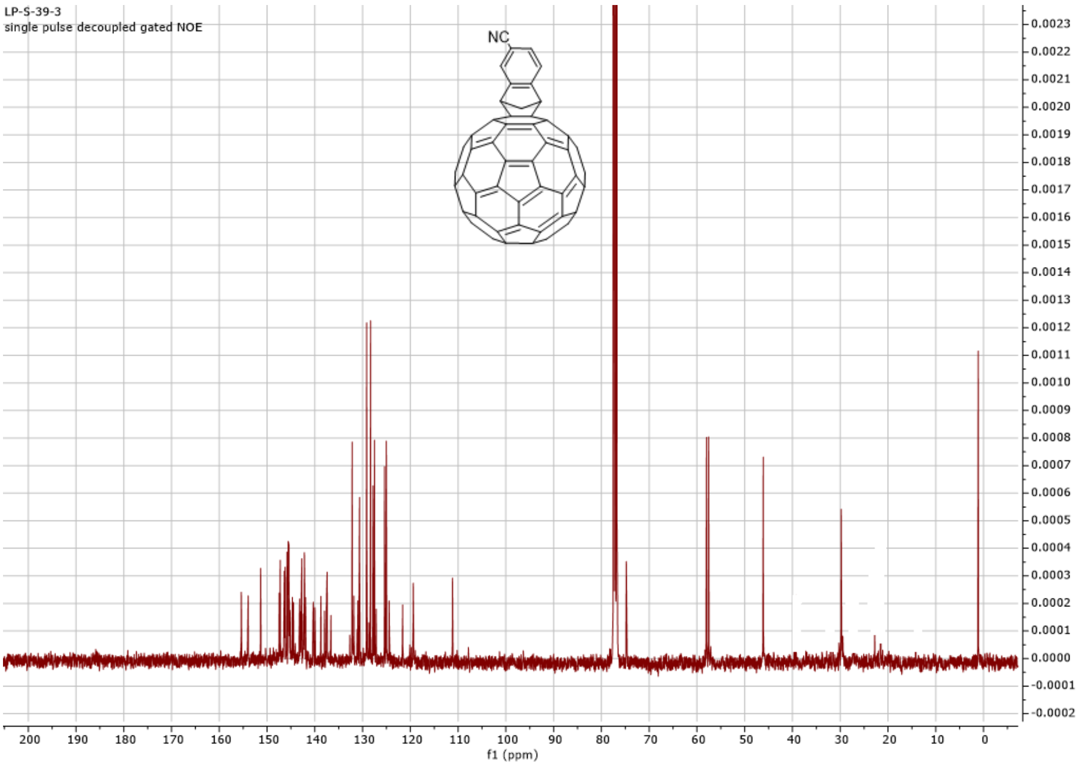

NH-Ac-ICMA

^{1}H NMR

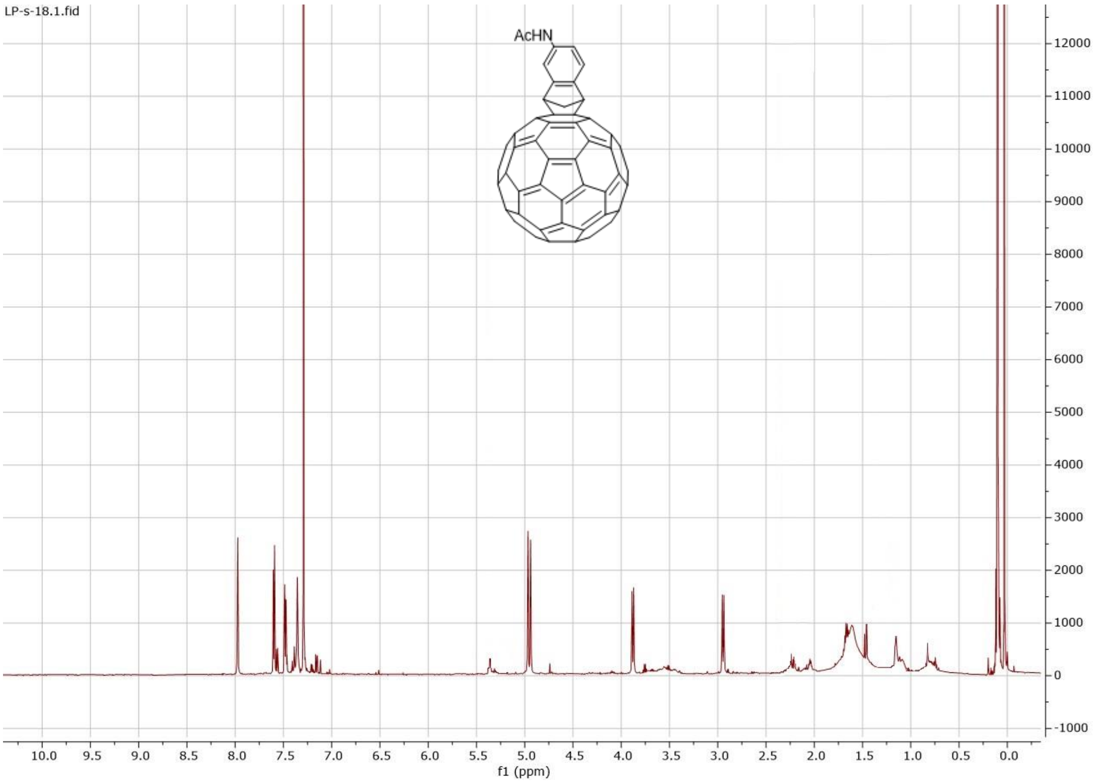

^{13}C NMR

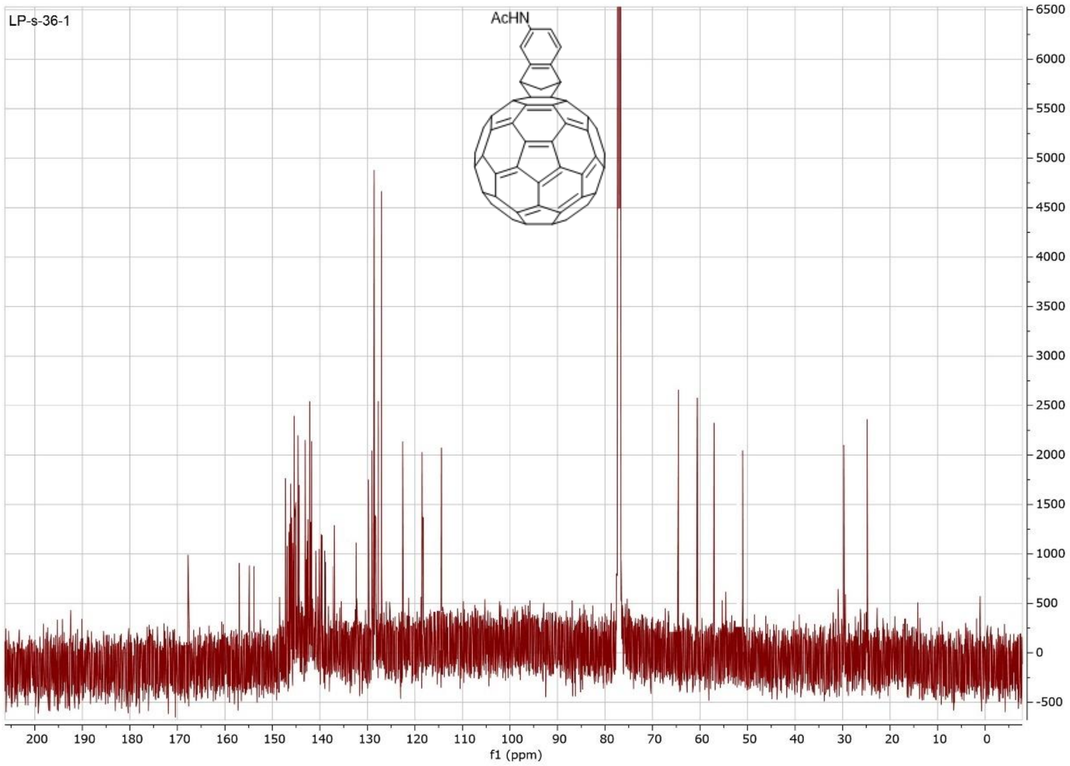

NH-Ac-Me-ICMA

^{1}H NMR

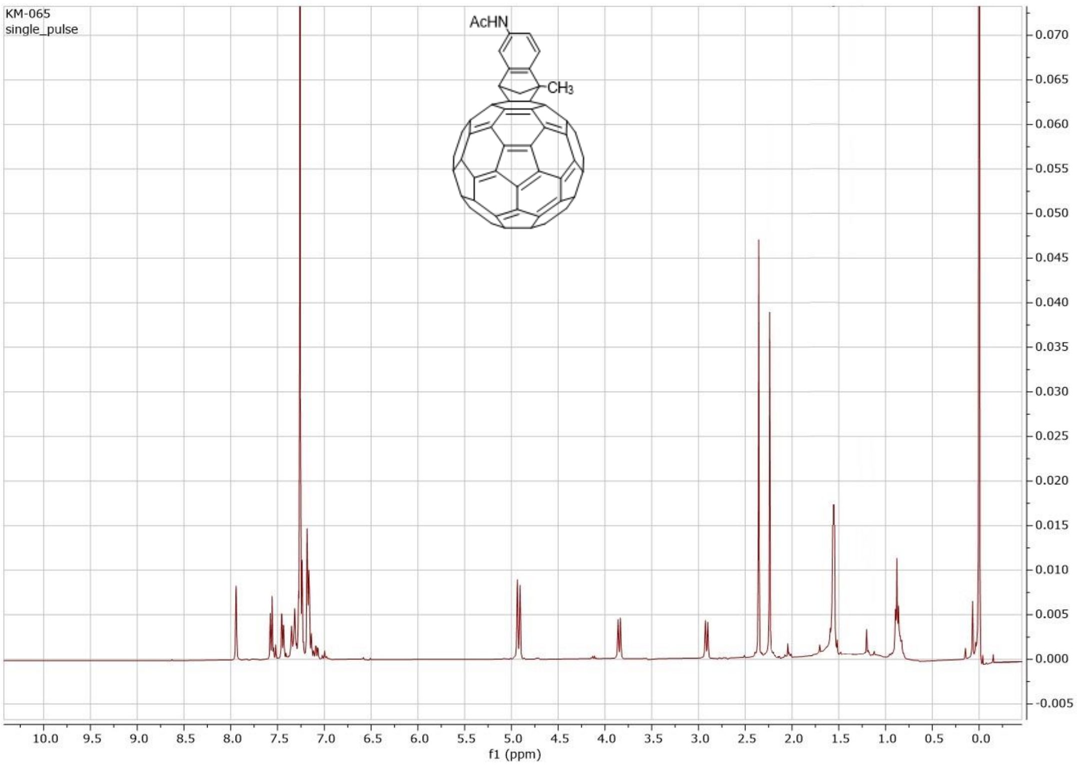

^{13}C NMR

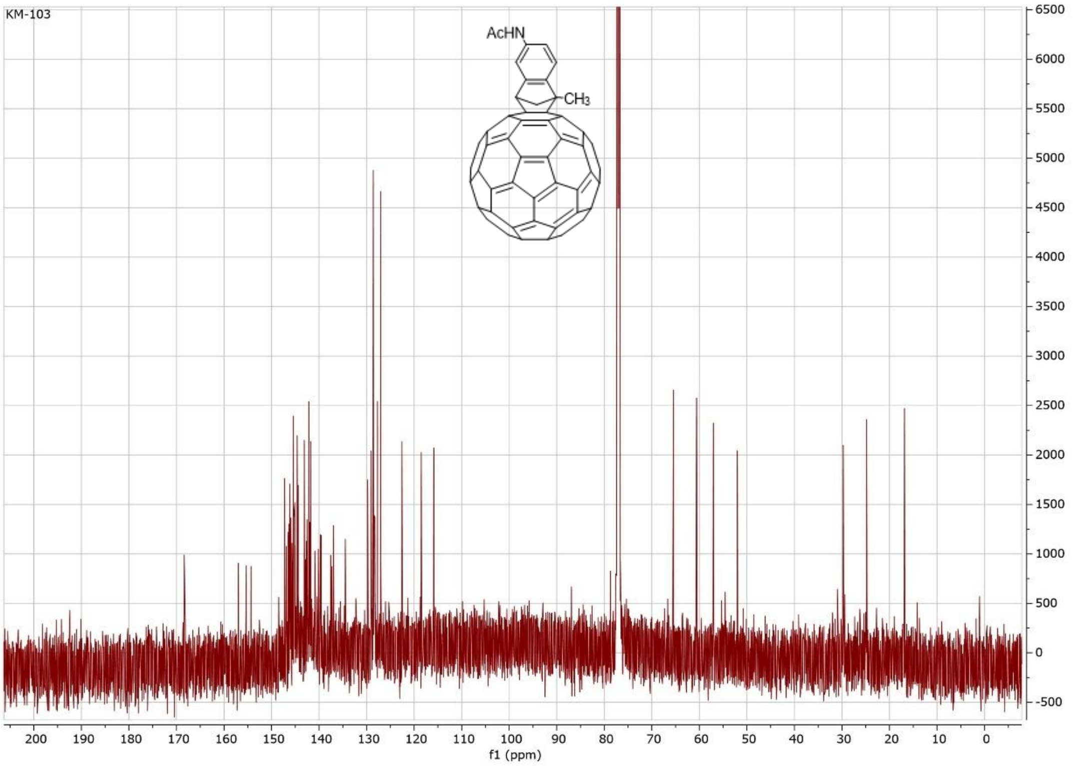

NH$_2$-ICMA

^{1}H NMR

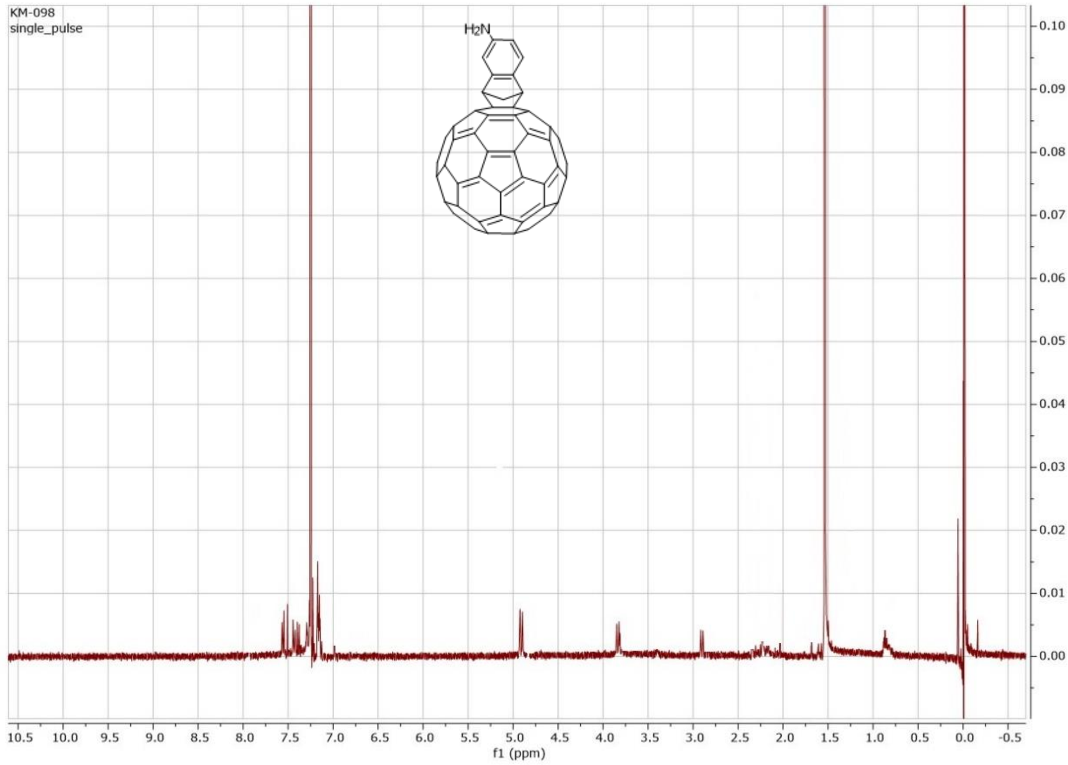

^{13}C NMR

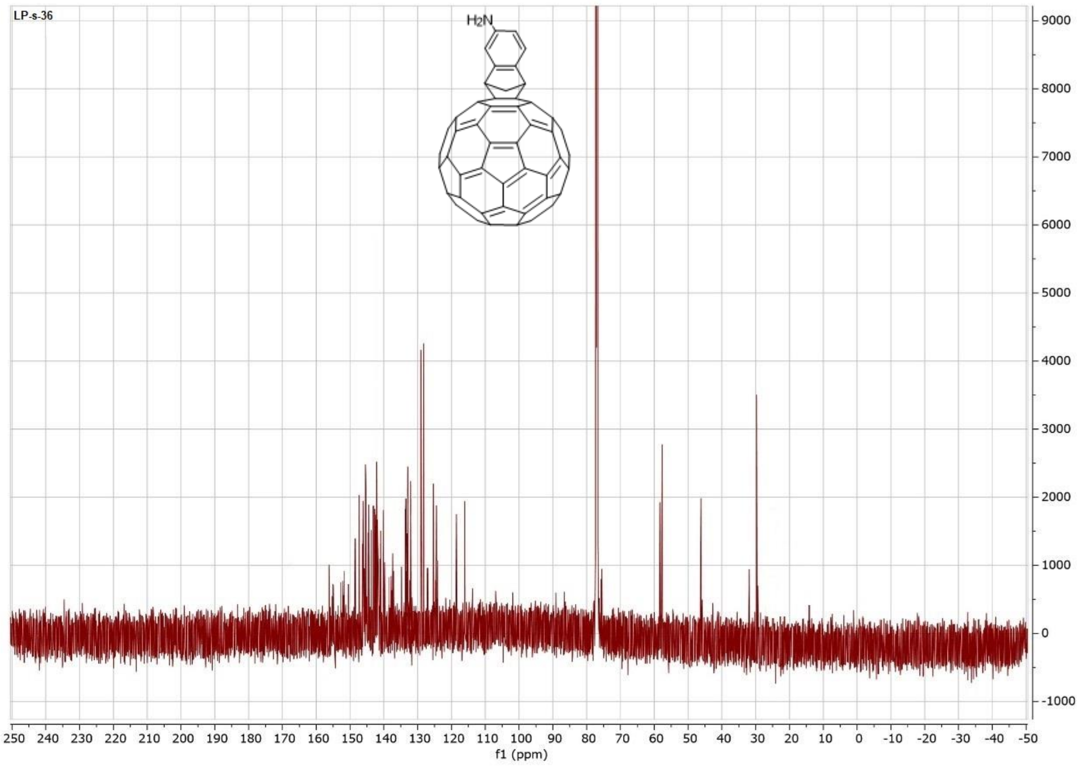

HR-MS

Benzo-ICMA HRMS (ESI) *m/z*: $[M]^+$ Calcd for $C_{73}H_{11}$ 887.0861; Found: 887.0856

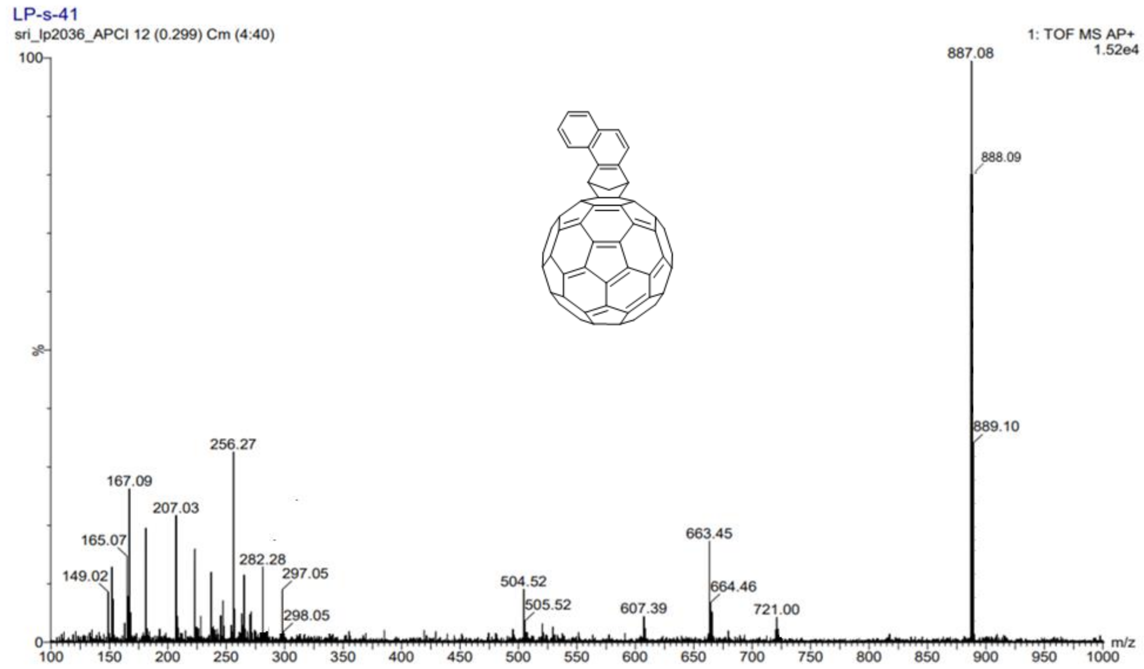

CN-ICMA HRMS (ESI) *m/z*: $[M]^+$ Calcd for $C_{70}H_8N$ 862.0657; Found: 862.0648

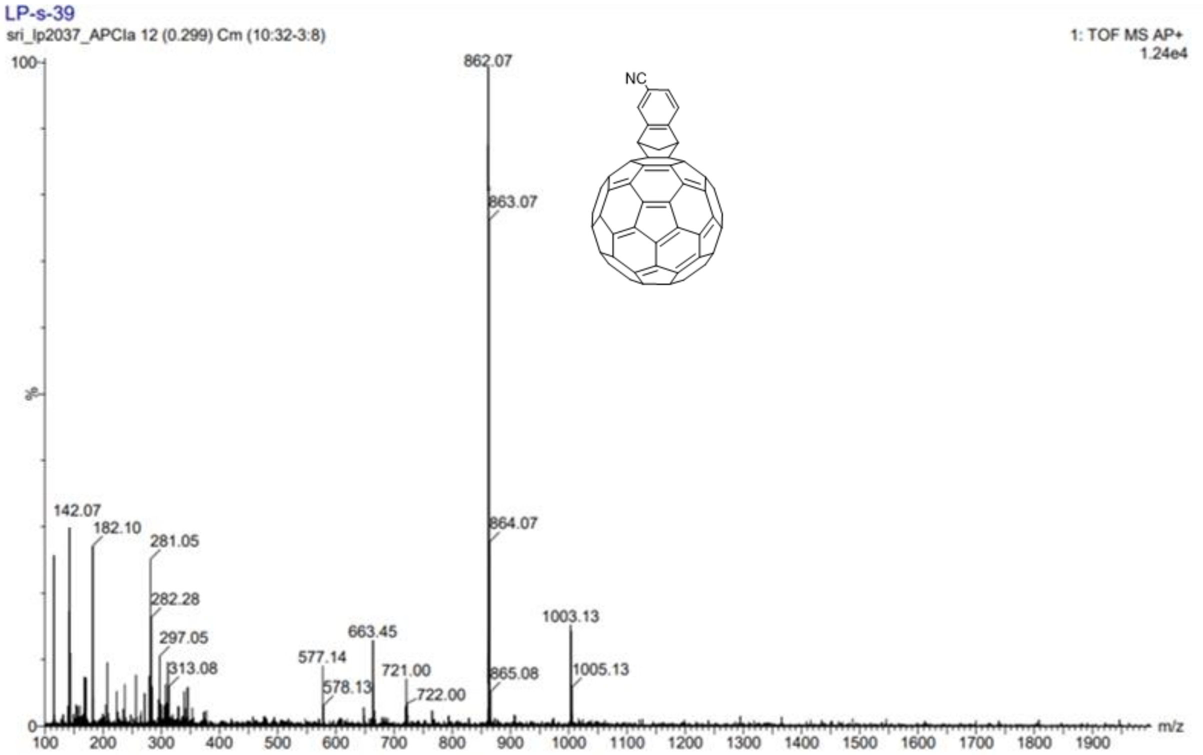

NH-Ac-ICMA HRMS (ESI) *m/z*: $[M + Na]^+$ Calcd for $C_{71}H_{11}NONa$ 916.0738; Found: 916.0728

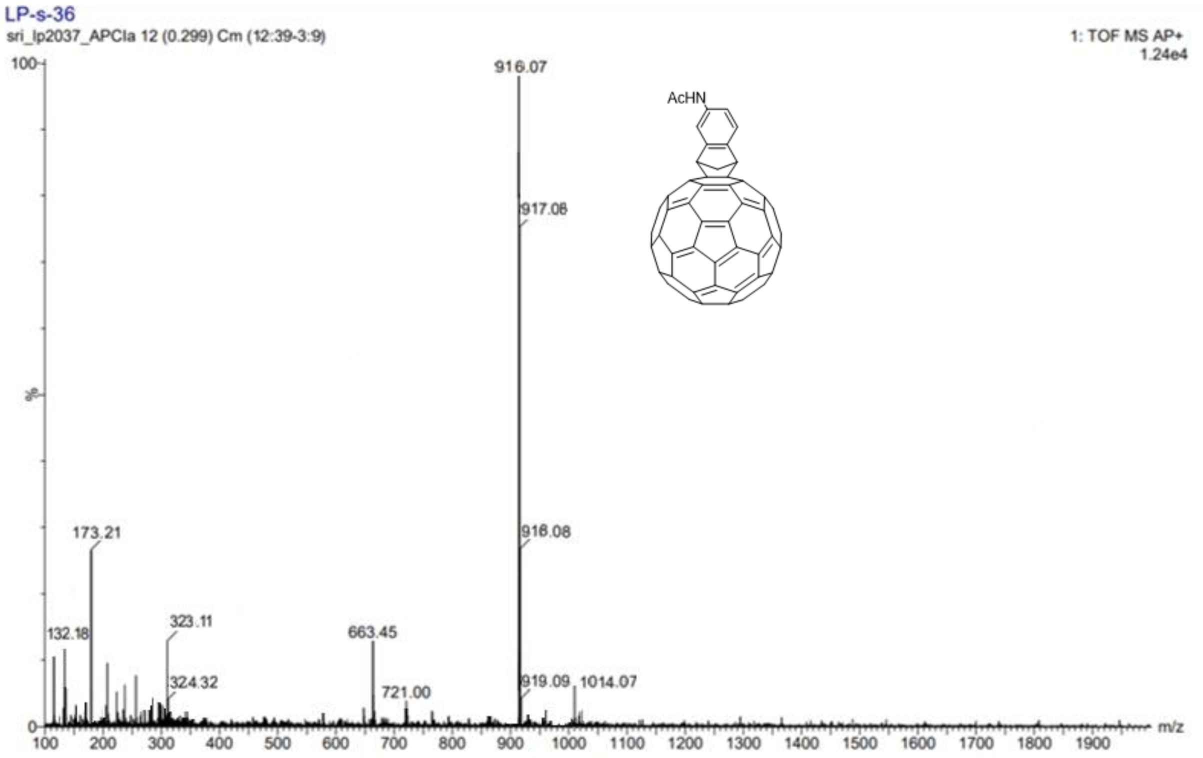

NH-Ac-Me-ICMA HRMS (ESI) *m/z*: $[M + Na]^+$ Calcd for $C_{72}H_{13}NONa$ 930.0895; Found: 930.0868

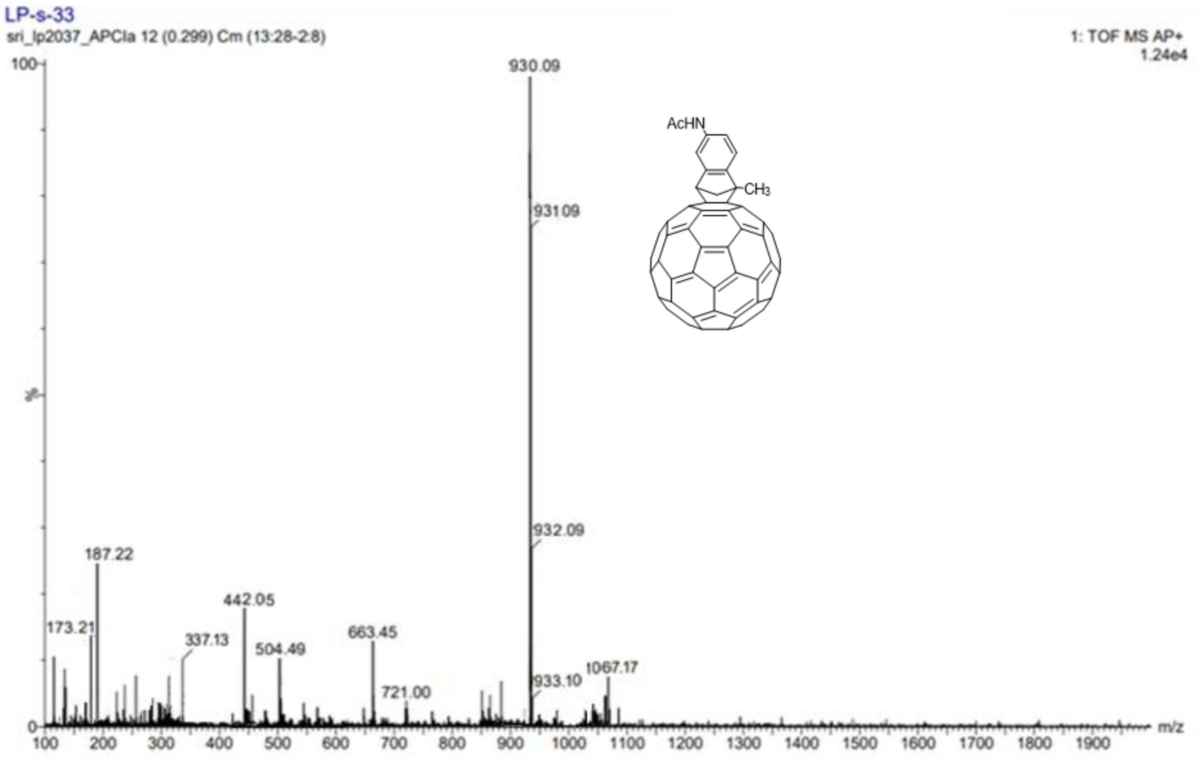

NH_2-ICMA HRMS (ESI) *m/z*: $[M]^+$ Calcd for $C_{69}H_9N$ 852.0813; Found: 852.0794

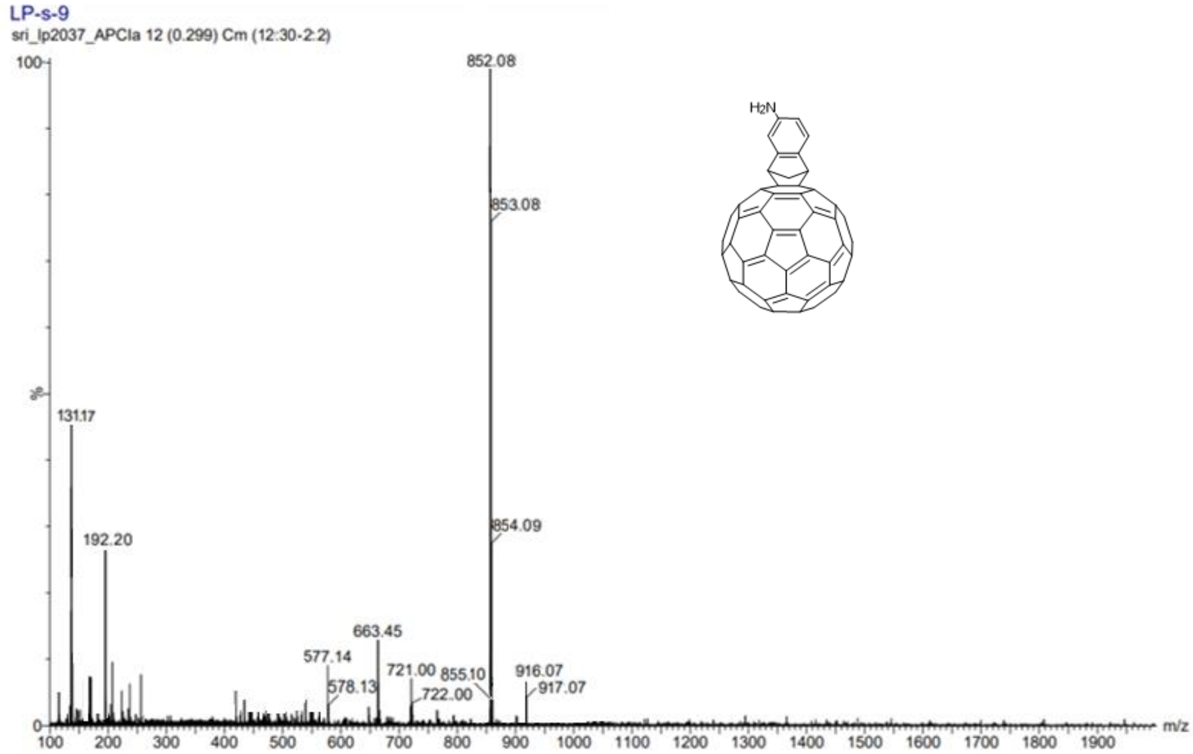

1.5. REFERENCES

(1) Ahmad, T.; Wilk, B.; Radicchi, E.; Fuentes Pineda, R.; Spinelli, P.; Herterich, J.; Castriotta, L. A.; Dasgupta, S.; Mosconi, E.; de Angelis, F.; Kohlstädt, M.; Würfel, U.; di Carlo, A.; Wojciechowski, K. New Fullerene Derivative as an N-Type Material for Highly Efficient, Flexible Perovskite Solar Cells of a p-i-n Configuration. *Advanced Functional Materials* **2020**, *30* (45), 1–11. https://doi.org/10.1002/adfm.202004357.

(2) Caprioglio, P.; Stolterfoht, M.; Wolff, C. M.; Unold, T.; Rech, B.; Albrecht, S.; Neher, D. On the Relation between the Open-Circuit Voltage and Quasi-Fermi Level Splitting in Efficient Perovskite Solar Cells. *Advanced Energy Materials* **2019**, *9* (33), 1901631. https://doi.org/10.1002/aenm.201901631.

(3) Wolff, C. M.; Caprioglio, P.; Stolterfoht, M.; Neher, D. Nonradiative Recombination in Perovskite Solar Cells: The Role of Interfaces. *Advanced Materials* **2019**, *31* (52), 1902762. https://doi.org/10.1002/adma.201902762.

(4) Caprioglio, P.; Wolff, C. M.; Sandberg, O. J.; Armin, A.; Rech, B.; Albrecht, S.; Neher, D.; Stolterfoht, M. On the Origin of the Ideality Factor in Perovskite Solar Cells. *Advanced Energy Materials* **2020**, *2000502*, 1–9. https://doi.org/10.1002/aenm.202000502.

(5) Sutanto, A. A.; Caprioglio, P.; Drigo, N.; Hofstetter, Y. J.; Garcia-Benito, I.; Queloz, V. I. E.; Neher, D.; Nazeeruddin, M. K.; Stolterfoht, M.; Vaynzof, Y.; Grancini, G. 2D/3D Perovskite

Engineering Eliminates Interfacial Recombination Losses in Hybrid Perovskite Solar Cells. *Chem* **2021**, 1–14. https://doi.org/10.1016/j.chempr.2021.04.002.

(6) Kramarenko, M.; Ferreira, C. G.; Martínez-Denegri, G.; Sansierra, C.; Toudert, J.; Martorell, J. Relation between Fluorescence Quantum Yield and Open-Circuit Voltage in Complete Perovskite Solar Cells. *Solar RRL* **2020**, *4* (4). https://doi.org/10.1002/solr.201900554.

(7) Murgatroyd, P. N. Theory of Space-Charge-Limited Current Enhanced by Frenkel Effect. *Journal of Physics D: Applied Physics* **1970**, *3* (2), 151–156. https://doi.org/10.1088/0022-3727/3/2/308.

(8) Gaussian 16, Revision C.01, M. J. Frisch, et al., Gaussian, Inc., Wallingford CT. Inc., Wallingford CT 2016.

(9) Becke, A. D. Density-Functional Thermochemistry. III. The Role of Exact Exchange. *The Journal of Chemical Physics* **1993**, *98* (7), 5648–5652. https://doi.org/10.1063/1.464913.

(10) Stephens, P. J.; Devlin, F. J.; Chabalowski, C. F.; Frisch, M. J. Ab Initio Calculation of Vibrational Absorption and Circular Dichroism Spectra Using Density Functional Force Fields. *Journal of Physical Chemistry®* **1994**, *98* (45), 11623–11627. https://doi.org/10.1021/j100096a001.

(11) Cossi, M.; Rega, N.; Scalmani, G.; Barone, V. Energies, Structures, and Electronic Properties of Molecules in Solution with the C-PCM Solvation Model. *Journal of Computational Chemistry* **2003**, *24* (6), 669–681. https://doi.org/10.1002/jcc.10189.

(12) Grimme, S.; Antony, J.; Ehrlich, S.; Krieg, H. A Consistent and Accurate Ab Initio Parametrization of Density Functional Dispersion Correction (DFT-D) for the 94 Elements H-Pu. *Journal of Chemical Physics* **2010**, *132* (15), 154104. https://doi.org/10.1063/1.3382344.
